# Dissection of Pharmacological Mechanism of Chinese Herbal Medicine Yihuo Huatan Formula on Chronic Obstructive Pulmonary Disease: A Systems Pharmacology-Based Study

**DOI:** 10.1038/s41598-019-50064-9

**Published:** 2019-09-17

**Authors:** Xia-Wei Zhang, Wei Liu, Hong-Li Jiang, Bing Mao

**Affiliations:** 1 Division of Respiratory Medicine, Department of Integrated Traditional Chinese and Western Medicine, West China Hospital, Sichuan University, 37 Guoxue Lane, Chengdu, Sichuan 610041 P. R. China; 2 Division of Pulmonary Diseases, State Key Laboratory of Biotherapy, West China Hospital, West China School of Medicine, Sichuan University, 1 Keyuansi Road, Chengdu, Sichuan 610041 P. R. China

**Keywords:** Clinical pharmacology, Chronic obstructive pulmonary disease

## Abstract

Chronic obstructive pulmonary disease (COPD) is one of the most common respiratory diseases. Yihuo Huatan Formula (YHF), as a proven Chinese Herbal Medicine (CHM), has been verified to be effective in the treatment of stable COPD through years’ of practice. Nevertheless, its working mechanism is still unclear. We sought to systematically decipher the mechanism of YHF for treating stable COPD using systems pharmacology-based method that integrates pharmacokinetic screening, target prediction, network analyses, GO and KEGG enrichment analyses. Firstly, a total of 1267 chemicals out of 15 herbal components were included in YHF chemical database. Among them, 180 potential active molecules were screened out through pharmacokinetic evaluation. Then 258 targets of the active molecules were predicted, of which 84 were chosen for further analyses. Finally, the network analyses and GO and KEGG enrichment methods suggested a therapeutic effect of YHF on the alleviation of airway inflammation, decrease of mucus secretion, maintenance of immune homeostasis and benefit of COPD comorbidities, by regulating multiple targets and pathways. The systems pharmacology-based approach helps to understand the underlying working mechanism of YHF in stable COPD from a holistic perspective, and offers an exemplification for systematically uncovering the action mechanisms of CHM.

## Introduction

Chronic obstructive pulmonary disease (COPD) is a common preventable and treatable disease characterized by persistent respiratory symptoms and airflow limitation^1^. It is estimated to rank as the third leading cause of death, and account for 8.6% of total deaths in the world by 2030^2^. Particularly in China, the prevalence of COPD among people over 40 years old rose from 8.2%^3^ to 13.7%^4^ during the past decade, accompanied by increasing hospitalization rate and mortality risk.

To date, conventional pharmacological therapies for stable COPD mainly include inhaled long-acting beta-agonists (LABA), long-acting muscarinic antagonists (LAMA) and corticosteroids. However, some side-effects of these therapies can’t be neglected. Both LABA and LAMA have considerable influence on cardiac function and were associated with increased risk of cardiovascular disease-related adverse events in COPD patients^5–7^. Besides, long-term corticosteroid therapy might result in immune disorders and increased risk of pneumonia and other respiratory infections^8^. Moreover, these conventional therapies also have limitations and have been insufficient to reliably ease mucus hypersecretion^9^, improve immune disorders, or benefit comorbid conditions during COPD in a meaningful way.

Chinese Herbal Medicine (CHM) has been widely used for stable COPD in light of their reliable therapeutic efficacy and good safety^10^. With a mixture of complex compounds, CHM is considered to aim at various biological targets and to exert multiple therapeutic efficacies. In the theoretical system of traditional Chinese medicine (TCM), patients with stable COPD mainly suffer from “vital Qi deficiency, blood stagnation and phlegm overproduction”, and the therapeutic principle of “tonifying Qi, dispersing blood stasis and dissolving phlegm” has been generally acceptable and extensively applied in the treatment of stable COPD^11,12^. Yihuo Huatan Formula (YHF) is an agreement CHM prescription prescribed based on the aforementioned therapeutic principle. It is composed of fifteen Chinese medicinal herbs (Table 1). Our previous clinical trial (funded by Sichuan Provincial Administration of Traditional Chinese Medicine, project number: 2016Z007; Clinical trial registration number: ChiCTR-INR-16010038) recruited 80 stable COPD patients and showed that YHF significantly decreased TCM syndrome scores (*P* = 0.001) and COPD Assessment Test scores (*P* = 0.040), increased curative effect rate of TCM syndromes (*P* = 0.001). Besides, annual exacerbations (*P* = 0.032) and hospitalizations (*P* = 0.043), as well as length of hospital stay (*P* = 0.027) were significantly reduced in YHF treatment group. We also found an improvement in some lung function indexes (forced vital capacity, forced expiratory volume in one second and % predicted) and six-minute walk distance in YHF treatment group. We did not observe any obvious side effects in one-year follow up. Additionally, we measured inflammatory cytokines in peripheral blood and induced sputum, and found a decrease in tumor necrosis factor (TNF)-α, interleukin (IL)-6, and IL-1β level, and an increase in IL-10 level after YHF treatment. The results suggested that YHF is effective and safe in the treatment of stable COPD, and might alleviate chronic airway inflammations. However, the active substances and pharmacological mechanism of YHF are not yet to be studied comprehensively.

**Table 1:** Herbal components of Yihuo Huatan Formula.

No.	Chinese name	Chinese Pinyin name	Latin name	English name
1	黄芪	Huangqi	*Hedysarum Multijugum Maxim*.	Milkvetch Root
2	党参	Dangshen	*Codonopsis Radix*	Tangshen
3	白术	Baizhu	*Atractylodes Macrocephala Koidz*.	Largehead Atractylodes Rhizome
4	山药	Shanyao	*Rhizoma Dioscoreae*	Common Yan Rhizome
5	茯苓	Fuling	*Poria Cocos(Schw*.*) Wolf*.	Poria
6	法半夏	Fabanxia	*Arum Ternatum Thunb*.	Pinellia Tuber
7	陈皮	Chenpi	*Citrus Reticulata*	Dried Tangerine peel
8	瓜蒌	Gualou	*Trichosanthes Kirilowii Maxim*	Snakegourd Fruit
9	赤芍	Chishao	*Radix Paeoniae Rubra*	Red Peony Root
10	川芎	Chuanxiong	*Chuanxiong Rhizoma*	Szechwan Lovage Rhizome
11	桃仁	Taoren	*Persicae Semen*	Peach Seed
12	熟地黄	Shudihuang	*Rehmanniae Radix Praeparata*	Rehmannia Root
13	山茱萸	Shanzhuyu	*Cornus Officinalis Sieb*. *Et Zucc*.	Asiatic Cornelian Cherry Fruit
14	淫羊藿	Yinyanghuo	*Epimrdii Herba*	Epimedium Herb
15	防风	Fangfeng	*Saposhnikoviae Radix*	Divaricate Saposhnikovia Root

**Figure 1:**
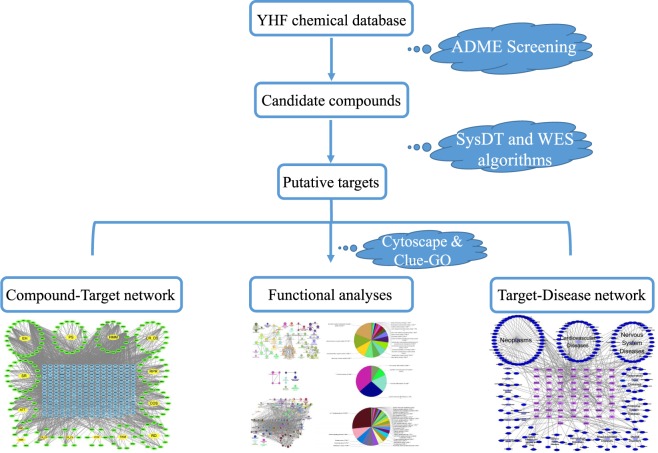
Flowchart of the systems pharmacology-based study. A systems pharmacology-based method was developed to explore action mechanisms of Yihuo Huatan Formula for treating chronic obstructive pulmonary disease. Abbreviations: YHF: Yihuo Huatan Formula; ADME: absorption, distribution, metabolism, excretion; SysDT: Systems Drug Targeting; WES: Weighted Ensemble Similarity.

The complex chemical compounds of YHF make it hard to clarify its pharmacological mechanism. Systems pharmacology, as an emerging field, integrates biological and pharmacological analysis to analyze the synergistic mechanism of CHMs in various diseases^13,14^. It provides insights into active compounds screening and putative targets prediction for CHM^15^. In the present work, we adopted systems pharmacology-based approach to advance the process in discovering and understanding the therapeutic mechanism of YHF. Figure 1 shows the brief flowchart of our study.

## Results

### Chemical database of Yihuo Huatan Formula

Taken together, a total of 1267 chemicals were included in YHF chemical database (see Supplementary Table S1): 87 from *Hedysarum Multijugum Maxim*. (HMM), 134 from *Codonopsis Radix* (CR_DS), 55 from *Atractylodes Macrocephala Koidz*. (AMK), 71 from *Rhizoma Dioscoreae* (RD), 34 from *Poria Cocos (Schw*.*) Wolf*. (PCW), 116 from *Arum Ternatum Thunb*. (ATT), 63 from *Citrus Reticulata* (CR_CP), 80 from *Trichosanthes Kirilowii Maxim* (TKM), 119 from *Radix Paeoniae Rubra* (RPR), 189 from *Chuanxiong Rhizoma* (CR_CX), 66 from *Persicae Semen* (PS), 76 from *Rehmanniae Radix Praeparata* (RRP), 226 from *Cornus Officinalis Sieb*. *Et Zucc*. (COS), 130 from *Epimrdii Herba* (EH) and 173 from *Saposhnikoviae Radix* (SR).

### Candidate active compounds

180 compounds with oral bioavailability (OB) ≥ 30% and drug-likeness (DL) ≥ 0.18 were considered as bioactive compounds (see Supplementary Table S2). The numbers of active substance in CR_DS, AMK, RD, PCW, ATT, CR_CP, TKM, RPR, CR_CX, PS, RRP, COS, EH and SR was 20, 21, 7, 16, 15, 13, 5, 11, 29, 7, 23, 2, 20, 23 and 18, respectively.

### Potential treatment targets

A total of 258 targets (see Supplementary Table S3) were predicted on the basis of Systems Drug Targeting (SysDT) and Weighted Ensemble Similarity (WES) algorithms from 128 candidate compounds, with 52 candidate compounds hitting no corresponding targets. After calculating the degree value, 84 important targets were selected for further study eventually. Among them, representative respiratory diseases-related targets included IL-6, IL-10, IL-1β, TNF-α, interferon (IFN)-γ, epidermal growth factor (EGF) and its receptor (EGFR), TNF-α, transforming growth factor (TGF)-β1, etc.

### Compound-Target and Target-Disease networks

#### Compound-Target network

**Figure 2:**
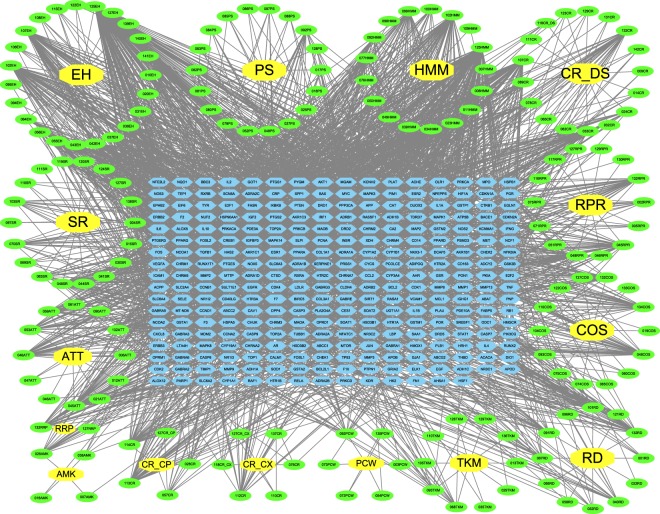
Compound-Target Network. The compound-target network was constructed by linking Yihuo Huatan Formula’s candidate compounds and their potential targets. The nodes represent herbs (yellow octagon), candidate compounds (green ellipse) and targets genes (blue hexagon). Abbreviations: HMM: *Hedysarum Multijugum Maxim*.; CR_DS: *Codonopsis Radix*; AMK: *Atractylodes Macrocephala Koidz*.; RD: *Rhizoma Dioscoreae*; PCW: *Poria Cocos (Schw*.*) Wolf*.; ATT: *Arum Ternatum Thunb*.; CR_CP: *Citrus Reticulata*; TKM: *Trichosanthes Kirilowii Maxim*; RPR: *Radix Paeoniae Rubra*; CR_CX: *Chuanxiong Rhizoma*; PS: *Persicae Semen*; RRP: *Rehmanniae Radix Praeparata*; COS: *Cornus Officinalis Sieb*. *Et Zucc*.; EH: *Epimrdii Herba*; SR: *Saposhnikoviae Radix*.

We constructed a Compound-Target (C-T) network based on the candidate active compounds of YHF and potential targets. As shown in Fig. 2, the C-T network embodied 401 nodes (15 herbs, 128 candidate compounds and 258 potential targets) and 2562 compound-target interactions. The mean degree of candidate compounds was 13.39. Among them, 40 compounds possessed degree value higher than 13.39. Specially, quercetin, kaempferol and luteolin acted on 149, 63 and 57 targets, respectively, which made them the representative crucial active compounds of YHF due to their important roles in C-T network. As to the targets, prostaglandin G/H synthase 2 (PTGS2), nuclear receptor coactivator (NCOA), prostaglandin G/H synthase 1 (PTGS1), heat shock protein HSP 90 (HSP90AA1) and progesterone receptor (PGR) were targeted by 108, 91, 70, 58 and 54 compounds, respectively, which indicated that these protein might be largely involved in the underlying action mechanisms of YHF.

#### Target-Disease network

**Figure 3:**
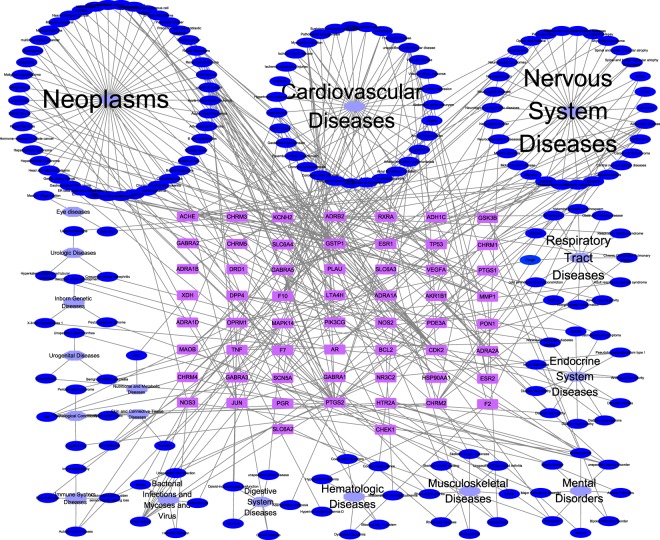
Target-Disease Network. The target-disease network was built by linking the potential targets and their corresponding diseases. 58 target genes (purple rectangle) were connected to 183 diseases (dark blue ellipse), which were divided into 18 categories (light blue octagon) according to Medical Subject Headings.

Target-Disease network (T-D) network was constructed on the basis of potential targets and corresponding diseases (see Supplementary Table S4). As shown in Fig. 3, the T-D network included 480 nodes (58 targets, 183 target-related diseases and 18 disease categories) and 467 target-disease interactions. 183 diseases were classified into 18 groups according to the MeSH Browser. Most of the collected diseases belonged to neoplasms (45/183), followed by cardiovascular diseases (31/183), nervous system diseases (30/183) and respiratory tract diseases (12/183).

#### Respiratory tract-related compound-target-disease network

**Figure 4:**
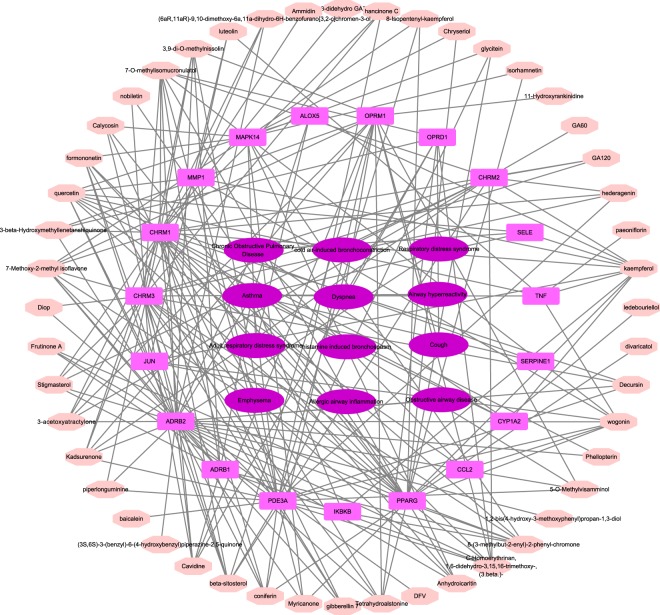
Respiratory Tract-Related Compound-Target-Disease Network. The respiratory tract-related compound-target-disease network was built by linking the respiratory tract-related compounds, targets and diseases. 48 compounds (pink octagon), 19 targets (peach round rectangle) and 12 respiratory tract diseases (purple ellipse) were linked together.

Respiratory tract-related compound-target-disease (C-T-D) network was constructed based on respiratory tract-related diseases, their corresponding targets and candidate compounds (Fig. 4). Respiratory tract-related C-T-D network included 79 nodes (48 compounds, 19 targets and 12 respiratory tract diseases) and 219 C-T-D interactions. Specially, quercetin, kaempferol and 7-O-methylisomucronulatol acted on 10, 10 and 9 targets, respectively. Therefore they were representative respiratory disease-related active compounds in YHF. As regards the targets, beta-2 adrenergic receptor (ADRB2), muscarinic acetylcholine receptor M1 (CHRM1), CGMP-inhibited 3′,5′-cyclic phosphodiesterase A (PDE3A) possessed the highest degree (34, 28 and 23, respectively). The 12 respiratory tract diseases consisted of COPD, asthma, emphysema, cough, dyspnea, airway hyperreactivity, allergic airway inflammation, cold air-induced bronchoconstriction, histamine induced bronchospasm, respiratory distress syndrome and so on. Among them, asthma and obstructive pulmonary disease were hit by the most target proteins, 10 and 6, respectively. Thus, the network analyses showed that YHF was well designed to treat chronic airway inflammatory disorders.

### Functional analyses

#### Gene ontology analysis

**Figure 5:**
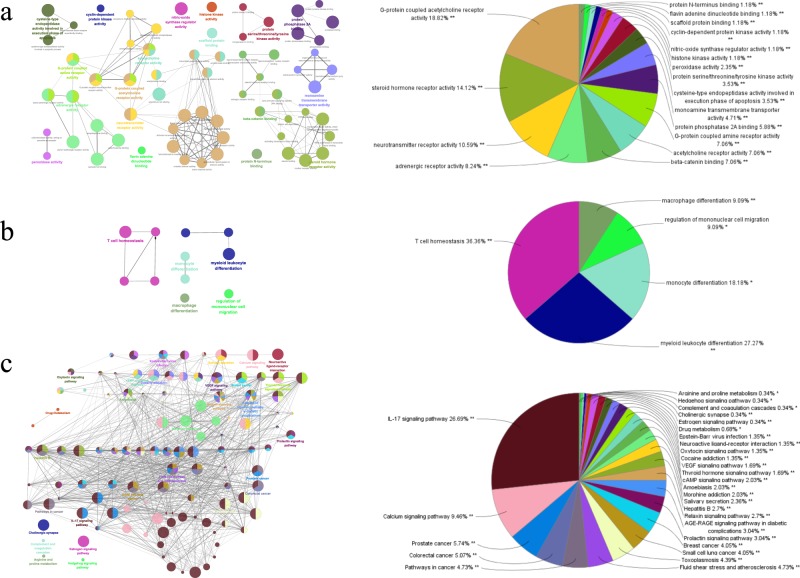
GO analyses and KEGG pathway enrichment analyses of predicted targets. Functionally grouped network of enriched clusters was generated from the target genes, representing the molecular functions (**a**), immune system processes (**b**) and KEGG pathway enrichment analyses (**c**) of predicted targets. Nodes represent GO terms, and the size of nodes shows the enrichment significance of GO terms. Only those significant GO terms were labeled. The node pie charts in the right are percentage of GO terms per group, in accordance with the grouped networks in the left. (**a**) Molecular functions of predicted targets. (**b**) Immune system process of predicted targets. (**c**) KEGG pathway enrichment analyses of predicted targets.

As shown in Fig. 5a,b, results of Gene Ontology (GO) analysis consisted of two stratums: molecular functions and immune system processes. To be specific, for the molecular functions, most potential targets were related to G-protein coupled acetylcholine receptor activity (18.82%), steroid hormone receptor activity (14.12%), neurotransmitter receptor activity (10.59%) and adrenergic receptor activity (8.24%). As regards the immune system processes, T cell homeostasis constituted the largest proportion (36.36%) of all immunity-related mechanisms, followed by myeloid leukocyte differentiation (27.27%) and monocyte differentiation (18.18%).

#### Kyoto encyclopedia of genes and genomes analysis

As shown in Fig. 5c, IL-17 signaling pathway (26.69%) contributed to the major clusters that were enriched in Kyoto Encyclopedia of Genes and Genomes (KEGG) pathway analysis, with calcium signaling pathway (9.46%) ranking the second and cancer-related pathways (15.54%) ranking the third to fifth.

## Discussion

**Figure 6:**
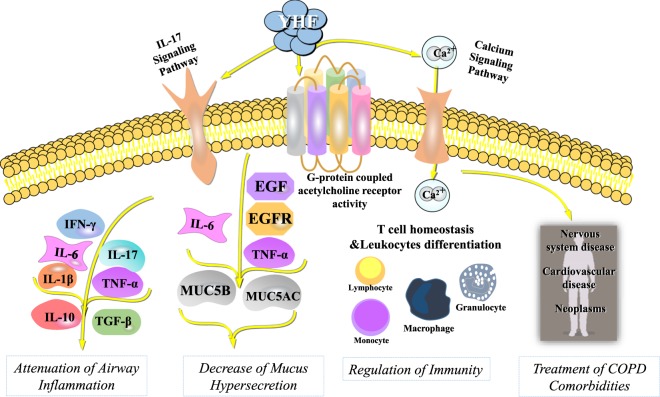
Illustration of the mechanisms of Yihuo Huatan Formula for treating chronic obstructive pulmonary disease. Abbreviations: YHF: Yihuo Huatan Formula; IFN-γ: interferon-γ; IL: interleukin; TNF-α: tumor necrosis factor-α; TGF-β1: transforming growth factor-β1; EGF: epidermal growth factor; EGFR: epidermal growth factor receptor; COPD: chronic obstructive pulmonary disease.

Adopting the systems pharmacology-based methods, we generally uncovered the working mechanism of YHF for stable COPD by exploring the crucial active compounds, targets and pathways (Fig. 6).

YHF was designed under a classical therapeutic principle of “tonifying Qi, dispersing blood stasis and dissolving phlegm”. Herbal components in YHF possess different efficacies and work cooperatively, with HMM, CR_DS, AMK and RD tonifying Qi, RPR, CR_CX and PS dispersing blood stasis, PCW, ATT, CR_CP and TKM dissolving phlegm. The prescription of YHF followed a unique and vital theoretical guidance of “Emperor-Minister-Assistant-Messenger”, also known as TCM composition theory. The emperor herbs are HMM, CR_DS, ATT and CR_CX, which are principal for treating the main symptoms and disorders. Major active compounds of YHF, such as quercetin, kaempferol, luteolin, stigmasterol and baicalein, occurred widely in these emperor herbs; The minister herbs include AMK, RD, CR_CP, RPR, PS, RRP and COS, aiming to assist the emperor in promoting a curative effect; The assistant herbs are PCW, TKM and EH, which are applied to modulate the effects of emperor and minister, alleviate toxicity and improve drug efficacy; Finally, the messenger herb SR plays an supporting role in harmonizing the actions and enhancing the functions of the other herbs. This combinatorial principle allows various components of a formula to work in a “network way”, where complex compounds-targets-diseases interactions present. The nature of the prescription principle is highly explained by the modern systems pharmacological approach. In our findings, there were 40 active substances with degree value more than 13.39 (mean value) in total. The emperor herbs contained 21 kinds of active substances, followed by 17 in minister herbs, 9 in assistant herbs and 8 in the messenger herb. Therefore, the emperor herbs contained more active compounds and played the most pivotal role in the therapeutic effect of YHF, compared with non-emperor herbs, which complied with the TCM composition theory. In the C-T network, emperor herbs mainly hit cytokines related to COPD airway inflammation and mucus hypersecretion such as IL-6, IL-10, IL-1β, TNF-α, IFN -γ, TGF-β1, EGF and EGFR, whilst messenger herbs hit more targets that were associated with airway damage and remodeling (eg. matrix metalloproteinase (MMP)-9)^16^, or hypercoagulability state of COPD (coagulation factors)^17^, which suggested that emperor herbs have main actions and the messenger herb has supporting role in treating COPD.

YHF might possess extensive anti-inflammatory activity in stable COPD through the function of bioactive compounds and treatment targets. Crucial active compounds of YHF such as quercetin and kaempferol have been strongly suggested to be effective in attenuating airway inflammation^18,19^. A lot of treatment targets screened by our study have also been suggested to play important pathological roles in inflammation development, including inflammatory cytokines such as IL-1β, TNF-α, IL-6, IFN -γ, IL-10 and TGF-β1^20^. Additionally, PTGS2 and PTGS1^21,22^, NCOA^23^ and HSP90AA1^24^, as the major targets hit by YHF, have also been widely documented to be closely associated with airway inflammation. Besides, the key pathway of YHF, IL-17 signaling pathway, has long been studied to be closely correlated with lung inflammation, emphysema formation, exacerbation and progression of COPD^25^.

Mucus hypersecretion was closely associated with COPD development, even in stable phase^26^. Our results indicated that YHF might alleviate mucus hypersecretion during COPD pathogenesis. It has been found that MUC5AC and MUC5B were the main mucin glycoproteins involved in excessive mucus formation in airways^27^. The expression of MUC5AC could be regulated by EGF and its receptor (EGFR)^28^ as well as TNF-α^29^, and MUC5B could be mediated by IL-6 and IL-17 pathway^30^. In our study, we found that EGF, EGFR, TNF-α, IL-6 and IL-17 were related to the working mechanisms of YHF. Besides, several compounds of YHF have been recognized as potential novel therapies to inhibit mucus synthesis and secretion in airway hypersecretory diseases. For example, luteolin was proved to help mucociliary clearance by increasing ciliary beat frequency in nasal cilia from COPD subjects^31^, while ellagic acid was found to be able to inhibit goblet cell hyperplasia in a mouse model with allergic airway inflammation^32^.

Recent studies have demonstrated that an extensive impaired T cells immunity resulted from chronic lung inflammation contributed to the exacerbations and progression of COPD^20^. Therapeutic targeting of dysfunctional T cells was considered to be beneficial in COPD management in efforts to reduce the inflammation and minimize tissue destruction. The GO analysis in our present study indicated that the therapeutic mechanism of YHF might be closely linked to the boosting of T cell homeostasis. Moreover, several candidate compounds of YHF have been documented to be immunomodulators with huge therapeutic potential in murine experiments, such as stigmasterol^33^ and baicalin^34^.

Greater understanding of the systemic inflammatory pathophysiology of COPD helped to explain the high frequency of major comorbidities including cancerous, cardiovascular, skeletal, endocrinic and mental disorders in addition to coexisting conditions that one would naturally expect due to the patients’ advanced age and shared risk factors. Comorbidities have significantly harmful influences on COPD patients in terms of quality of life, exacerbation frequency as well as survival, and bring greater challenges to COPD management^35^. Therapies for comorbidities are always suggested as an important part for COPD treatment. In the T-D network of our study, treatment targets of YHF were hit by neoplasms, cardiovascular diseases, nervous systems disease and mental disorders besides respiratory diseases. These suggested that YHF could provide extensive and pleiotropic pharmacological activities on co-existing diseases during COPD course, which was in line with the TCM concept of holism and the TCM principle of “homotherapy for heteropathy”.

Besides the above, targets of YHF also showed various biological functions linked to other aspects of COPD pathogenesis. For instance, EGFR, TGF-β and MMP-9 were important in the regulation of airway damage and airway remodeling^16,36^. Prothrombin, coagulation factor X, coagulation factor VII and tissue factor exhibited vital modulation function in hypercoagulability state and venous thromboembolism of COPD^17,37^.

There are still some limitations in our study. Firstly, our collection of bioactive components and targets from currently available resources may be not comprehensive. It can be improved using new detection technique such as high performance liquid chromatography-mass spectrometry (HPLC-MS). Secondly, the confidence in our results is limited due to a lack of further experimental verification. In the future, we will endeavor to verify the mechanisms of YHF in animal models. Thirdly, as YHF is a complex formula composed of 15 herbs, complicated herb-herb interactions may exist and contribute to the clinical effect. Besides, the “multi-component, multi-target” nature of YHF requires a comprehensive exploration on compound-target interactions. However, it is still difficult to uncover herb-herb interactions and specific action patterns between candidate compounds and target proteins using existing systems pharmacology-based methods. These difficulties present major challenges in the development of TCM.

## Conclusions

In conclusion, we provided systems pharmacology-based means to gain insights into the working mechanism of YHF for COPD by dissecting the bioactive compounds, potential targets and compounds-targets-diseases networks. Based on these findings, YHF contained plenty of bioactive compounds with different pharmacologic properties against multiple targets. The synergistic effects of YHF in COPD may be mainly realized through airway inflammation and mucus hypersecretion inhibition, immune restoration as well as comorbidities benefit.

## Materials and Methods

### Chemical database construction of Yihuo Huatan Formula

All ingredients of YHF were extracted from the Shanghai Institute of Organic Chemistry of CAS, Chemistry Database (http://www.organchem.csdb.cn), Traditional Chinese Medicines for Systems Pharmacology Database and Analysis Platform (http://lsp.nwu.edu.cn/index.php)^38^, NCBI PubChem database (https://pubchem.ncbi.nlm.nih.gov), DrugBank database (https://www.drugbank.ca/) and wide-scale literature mining, then we added them into the compounds database of YHF.

### Active ingredients prediction

In order to make the best use of high cost and time-consuming biological experiments and clinical research, the previous prediction to discover the potential active natural products from CHM formula is very necessary. In drug discovery and development process, absorption, distribution, metabolism and excretion (ADME) evaluations are critical procedures^39^ to predict biological active compounds. We used ADME screening to identify possible active compounds of YHF. Specifically, a combination of OB screening and DL property evaluation was applied to explore the active substance of YHF^38^

#### Oral bioavailability calculation

OB refers to the relative amount of the drug taken into the systemic circulation after extravascular administration^40^. Not only is OB an important index for the objective evaluation of drug absorption, but also a key factor to determine the successful moving of a new drug to late-phase clinical trial^41^. OBioavail 1.1 is a chemostatistical model based on 805 structurally diverse drug and drug-like molecules. It brought the P-glycoprotein (P-gp) and cytochrome P450s into construction and was used to predict the OB value of herbal ingredient^42^. In this study, we chose the ingredients with OB ≥ 30%^13,38^ as the candidate molecules for further analyses. We set this threshold mainly to screen the potential chemical components that could be orally absorbed and had good curative effect.

#### Drug-Likeness calculation

DL index refers to the structural similarity of the herbal ingredients to a known drug. During the process of drug development, DL is widely used to evaluate the potential of compounds to be drugs. Compounds with low DL value are chemically and pharmacologically unlikely to be drugs. In this study, we used the database-dependent DL prediction approach on the basis of Tanimoto similarity^43,44^ defined as follows:

1 $$T(A,B)=\frac{A\cdot B}{{|A|}^{2}+{|B|}^{2}-A\cdot B}$$

In this formula, “*A*” represents the molecular properties of compounds from YHF, and “*B*” represents the average DL value of all compounds in DrugBank database. In our study, we chose the molecules with DL value ≥ 0.18^13,38^ as candidate compounds with drug-like property. This threshold was set according to the average value of all DrugBank compounds.

Finally, OB ≥ 30% and DL value ≥ 0.18 were set as the threshold to select candidate active compounds. Additionally, for compounds that didn’t meet the criteria, we did a literature review to see whether they were supported by experimental evidence. If so, these compounds would also be obtained as candidate active compounds for further analyses.

### Potential treatment targets prediction

The therapeutic effect of a CHM formula relied on the synergies among multiple components, targets and pathways^45^. A systematic identification of potential treatment targets is vital for discovering the underlying working mechanisms of CHMs. Therefore, the ligand-based prediction strategies, SysDT^46^ and WES algorithms^47^ were employed to identify the putative treatment targets of the candidate active compounds. The SysDT, based on two powerful methods of Random Forest (RF) and Support Vector Machine (SVM) with high concordance, sensitivity and specificity, showed impressive performance on systematical prediction for drug-target associations and interactions involving enzymes, ion channels, nuclear receptors and G-protein coupled receptors^46^. Whilst WES model was on the basis of a large dataset involving 98,327 drug-target relationships derived from BindingDB (http://www.bindingdb.org/bind/index.jsp), DrugBank, PDB database (http://www.rcsb.org/pdb/), and National Center for Biotechnology Information Search database (https://www.ncbi.nlm.nih.gov/). And this algorithm mainly contained three phases: (1) identifying the structural, physical and chemical properties of key ligands that are highly-related to the pharmacological features in an ensemble framework by using CDK and Dragon software; (2) determining a drug’s affiliation to a target by assessing the weighted ensemble similarity; (3) integrating the standardized ensemble similarities (Z score) by Bayesian network and predicting the targets via multi-variate kernel approach. We chose the targets with RF score ≥ 0.7 or SVM ≥ 0.8 (SysDT) or Z score ≥ 7 (WES) as the potential targets.

Then, the Retrieve/ID mapping tool in UniProt database (https://www.uniprot.org/uploadlists/) was utilized to standardize the target related genes and to screen the targets from Homo Sapiens.

### Compound-Target and Target-Disease networks construction

CHM formula exerts complicated pharmacological effects through multiple compounds and targets. Thereby, we built C-T and T-D networks to help comprehensively understand the complex interactions of candidate active compounds and their corresponding targets at a systems level. We also built a respiratory tract-related C-T-D network to clearly show the interactions among respiratory tract-related compounds, target proteins and diseases.

All of the relationships between ingredients and targets constituted the C-T network. When it came to the T-D network, firstly, the related disease information on the basis of the potential targets was obtained from PharmGKB (https://www.pharmgkb.org/index.jsp), CTD database (http://ctdbase.org/) and TTD database (http://bidd.nus.edu.sg/group/cjttd/); secondly, these pieces of information were classified from Medical Subject Headings (MeSH) (https://www.nlm.nih.gov/mesh/MBrowser.html) and integrated into T-D network.

We used Cytoscape 3.6.1 software (http://www.cytoscape.org)^48^ to generate these networks and analyze their topological properties. In these networks, the degree of a node, which defined as the number of edges that connect to it (with loops counted twice), showed the importance of the node in the network. Degree was a very common and frequently used property to analyze C-T networks and T-D networks in published articles^13,49,50^, and was also recommended by TCMSP database^38^.

Then we calculated degree value of selected targets to screen the major targets in the network. Degree value was calculated in the vertical connections of C-T-D network. The higher degree a node has, the more significant it is. So only targets with values equal to or above the mean values were identified as major potential therapeutic targets. Furthermore, we chose targets with degree value ≥ 7 as important targets to do further functional analyses.

### Functional analyses

In order to decipher the potential molecular mechanisms of YHF, we did GO and KEGG (https://www.kegg.jp/kegg/pathway.html)^51^ pathway enrichment analyses using KEGG database, Database Visualization and Integrated Discovery system (DAVID, https://david.ncifcrf.gov/)^52^ and ClueGo assay (a Cytoscape plugin)^53^, aiming to find out biological process and molecular interactions of the selected targets and identifying the biological interrelations of functional groups.

## Supplementary information

Supplementary Table S1, S2, S3, S4

## Data Availability

Related materials and data included in our study are from open database resources which are available to public.
