# Supplementary material for: Dissection of Pharmacological Mechanism of Chinese Herbal Medicine Yihuo Huatan Formula on Chronic Obstructive Pulmonary Disease: A Systems Pharmacology-Based Study
Source: Sci Rep. 2019 Sep 17;9:13431. doi: 10.1038/s41598-019-50064-9 (PMC6748909; doi:10.1038/s41598-019-50064-9)
Supplement: Supplementary file 1 — Supplementary Table S1, S2, S3, S4 [file 41598_2019_50064_MOESM1_ESM.pdf]

Supplementary tables

Title: Dissection of Pharmacological Mechanism of Chinese Herbal Medicine Yihuo Huatan Formula on Chronic Obstructive Pulmonary Disease: A Systems Pharmacology-Based Study

Authors: Xia-Wei Zhang, Wei Liu, Hong-Li Jiang, Bing Mao

Supplementary Table S1. Pharmacokinetic property predictions for all compounds of Yihuo Huatan Formula

Molecule ID	Molecule Name	MW	OB (%)	DL	Herb
MOL000114	vanillic acid	168.16	35.47	0.04	*Hedysarum Multijugum Maxim.*
MOL000131	EIC	280.5	41.9	0.14	*Hedysarum Multijugum Maxim.*
MOL000211	Mairin	456.78	55.38	0.78	*Hedysarum Multijugum Maxim.*
MOL001955	Heriguard	354.34	11.93	0.33	*Hedysarum Multijugum Maxim.*
MOL000239	Jaranol	314.31	50.83	0.29	*Hedysarum Multijugum Maxim.*
MOL000251	Rhamnocitrin	300.28	12.9	0.27	*Hedysarum Multijugum Maxim.*
MOL000295	alexandrin	576.95	20.63	0.63	*Hedysarum Multijugum Maxim.*
MOL000296	hederagenin	414.79	36.91	0.75	*Hedysarum Multijugum Maxim.*
MOL000033	(3S,8S,9S,10R,13R,14S,17R)-10,13-dimethyl-17-[(2R,5S)-5-propan-2-yloctan-2-yl]-2,3,4,7,8,9,11,12,14,15,16,17-dodecahydro-1H-cyclopenta[a]phenanthren-3-ol	428.82	36.23	0.78	*Hedysarum Multijugum Maxim.*
MOL000354	isorhamnetin	316.28	49.6	0.31	*Hedysarum Multijugum Maxim.*
MOL000356	lupeol	426.8	12.12	0.78	*Hedysarum Multijugum Maxim.*
MOL000371	3,9-di-O-methylnissolin	314.36	53.74	0.48	*Hedysarum Multijugum Maxim.*
MOL000372	3-Hydroxy-2-picoline	109.14	62.47	0.02	*Hedysarum Multijugum Maxim.*
MOL000373	(2S)-4-methoxy-7-methyl-2-[1-methyl-1-[(2S,3R,4S,5S,6R)-3,4,5-trihydroxy-6-methylol-tetrahydropyran-2-yl]oxy-ethyl]-2,3-dihydrofuro[3,2-g]chromen-5-one	452.5	5.38	0.81	*Hedysarum Multijugum Maxim.*
MOL000374	5'-hydroxyiso-muronulatol-2',5'-di-O-glucoside	642.67	41.72	0.69	*Hedysarum Multijugum Maxim.*
MOL000375	5'-hydroxyiso-muronulatol-2',5'-di-O-glucoside_qt	480.51	3.65	0.8	*Hedysarum Multijugum Maxim.*
MOL000376	7,2'-dihydroxy-3',4'-dimethoxyisoflavone-7-O-β-D-glucoside	476.47	16.16	0.86	*Hedysarum Multijugum Maxim.*
MOL000377	7-hydroxy-3-(2-hydroxy-3,4-dimethoxy-phenyl)chromone	314.31	5.45	0.3	*Hedysarum Multijugum Maxim.*
MOL000378	7-O-methylisomucronulatol	316.38	74.69	0.3	*Hedysarum Multijugum Maxim.*
MOL000379	9,10-dimethoxypterocarpan-3-O-β-D-glucoside	462.49	36.74	0.92	*Hedysarum Multijugum Maxim.*
MOL000380	(6aR,11aR)-9,10-dimethoxy-6a,11a-dihydro-6H-benzofurano[3,2-c]chromen-3-ol	300.33	64.26	0.42	*Hedysarum Multijugum Maxim.*
MOL000381	13-hydroxy-9,11-octadecadienoic acid	296.5	35.6	0.17	*Hedysarum Multijugum Maxim.*
MOL000382	Arabinose,d	150.15	1.87	0.02	*Hedysarum Multijugum Maxim.*
MOL000383	D-Galacturonic acid, homopolymer	194.16	29.75	0.04	*Hedysarum Multijugum Maxim.*
MOL000384	DL-Glucuronic acid	194.16	3.35	0.04	*Hedysarum Multijugum Maxim.*
MOL005928	isoferulic acid	194.2	50.83	0.06	*Hedysarum Multijugum Maxim.*
MOL000386	Fucopyranose, L-	164.18	42.51	0.03	*Hedysarum Multijugum Maxim.*
MOL000387	Bifendate	418.38	31.1	0.67	*Hedysarum Multijugum Maxim.*
MOL000388	gamma-aminobutyric acid	103.14	24.09	0.01	*Hedysarum Multijugum Maxim.*
MOL000389	FERULIC ACID (CIS)	194.2	54.97	0.06	*Hedysarum Multijugum Maxim.*
MOL000390	daidzein	254.25	19.44	0.19	*Hedysarum Multijugum Maxim.*
MOL000391	Ononin	430.44	11.52	0.78	*Hedysarum Multijugum Maxim.*
MOL000392	formononetin	268.28	69.67	0.21	*Hedysarum Multijugum Maxim.*
MOL000393	Soyasaponin I	943.26	2.06	0.05	*Hedysarum Multijugum Maxim.*

MOL000394	choline	104.2	0.47	0.01	*Hedysarum Multijugum Maxim.*
MOL000395	GGB	176.21	54.95	0.03	*Hedysarum Multijugum Maxim.*
MOL000396	(+)-Syringaresinol	418.48	3.29	0.72	*Hedysarum Multijugum Maxim.*
MOL000397	cis-p-Coumarate	164.17	45.98	0.04	*Hedysarum Multijugum Maxim.*
MOL000398	isoflavanone	316.33	109.99	0.3	*Hedysarum Multijugum Maxim.*
MOL000399	Docosanoate	340.66	15.69	0.26	*Hedysarum Multijugum Maxim.*
MOL000400	Flavaxin	376.41	18.18	0.5	*Hedysarum Multijugum Maxim.*
MOL000401	astragalosideI	869.17	46.79	0.11	*Hedysarum Multijugum Maxim.*
MOL000402	astragalosideI_qt	707.01	12.34	0.2	*Hedysarum Multijugum Maxim.*
MOL000403	astragalosideII	827.13	46.06	0.13	*Hedysarum Multijugum Maxim.*
MOL000404	astragalosideII_qt	664.97	11.55	0.25	*Hedysarum Multijugum Maxim.*
MOL000405	astragalosideⅢ	785.09	31.83	0.1	*Hedysarum Multijugum Maxim.*
MOL000406	astragalosideⅢ_qt	622.93	5.35	0.32	*Hedysarum Multijugum Maxim.*
MOL000407	astragalosideⅣ	785.09	22.5	0.15	*Hedysarum Multijugum Maxim.*
MOL000408	astragalosideⅣ_qt	622.93	7.07	0.32	*Hedysarum Multijugum Maxim.*
MOL000409	AstragalosideIV	785.09	17.74	0.15	*Hedysarum Multijugum Maxim.*
MOL000410	AstragalosideIV_qt	622.93	7.07	0.32	*Hedysarum Multijugum Maxim.*
MOL000411	Astraisoflavanin	464.51	18.37	0.86	*Hedysarum Multijugum Maxim.*
MOL000412	Mucronulatol	302.35	4.22	0.26	*Hedysarum Multijugum Maxim.*
MOL000413	astrachrysoside A	769.09	24.55	0.1	*Hedysarum Multijugum Maxim.*
MOL000414	Caffeate	180.17	54.97	0.05	*Hedysarum Multijugum Maxim.*
MOL000415	rutin	610.57	3.2	0.68	*Hedysarum Multijugum Maxim.*
MOL000416	Lariciresinol	360.44	5.53	0.38	*Hedysarum Multijugum Maxim.*
MOL000417	Calycosin	284.28	47.75	0.24	*Hedysarum Multijugum Maxim.*
MOL000418	3'-Hydroxy-4'-methoxyisoflavone-7-O-beta-D-glucoside	446.44	10.05	0.81	*Hedysarum Multijugum Maxim.*
MOL000419	astrasieversianin XV	901.22	11.19	0.07	*Hedysarum Multijugum Maxim.*
MOL000420	XLS	150.15	51.08	0.02	*Hedysarum Multijugum Maxim.*
MOL000421	nicotinic acid	123.12	47.65	0.02	*Hedysarum Multijugum Maxim.*
MOL000422	kaempferol	286.25	41.88	0.24	*Hedysarum Multijugum Maxim.*
MOL000423	rhamnocitrin-3-O-glucoside	462.44	2.87	0.76	*Hedysarum Multijugum Maxim.*
MOL000424	RAM	164.18	50.5	0.04	*Hedysarum Multijugum Maxim.*
MOL000425	asernestioside A	931.25	11.07	0.03	*Hedysarum Multijugum Maxim.*
MOL000426	asernestioside A_qt	769.09	24.55	0.1	*Hedysarum Multijugum Maxim.*
MOL000427	asernestioside B	973.29	12.54	0.03	*Hedysarum Multijugum Maxim.*
MOL000428	asernestioside B_qt	811.13	14.03	0.09	*Hedysarum Multijugum Maxim.*
MOL000429	Crystal VI	132.14	83.96	0.02	*Hedysarum Multijugum Maxim.*
MOL000430	betaine	117.17	40.92	0.01	*Hedysarum Multijugum Maxim.*
MOL000431	coumarin	146.15	29.17	0.04	*Hedysarum Multijugum Maxim.*
MOL000432	linolenic acid	278.48	45.01	0.15	*Hedysarum Multijugum Maxim.*
MOL000433	FA	441.45	68.96	0.71	*Hedysarum Multijugum Maxim.*
MOL000434	acetylastragaloside I	911.21	43.54	0.09	*Hedysarum Multijugum Maxim.*
MOL000435	acetylastragaloside I_qt	749.05	30.75	0.17	*Hedysarum Multijugum Maxim.*
MOL000436	(Z)-1-(2,4-dihydroxyphenyl)-3-(4-hydroxyphenyl)prop-2-en-1-one	256.27	87.51	0.15	*Hedysarum Multijugum Maxim.*
MOL000437	Hirsutrin	464.41	1.86	0.77	*Hedysarum Multijugum Maxim.*
MOL000438	(3R)-3-(2-hydroxy-3,4-dimethoxyphenyl)chroman-7-ol	302.35	67.67	0.26	*Hedysarum Multijugum Maxim.*

MOL000439	isomucronulatol-7,2'-di-O-glucosiole	626.67	49.28	0.62	*Hedysarum Multijugum Maxim.*
MOL000440	isomucronulatol-7,2'-di-O-glucosiole_qt	464.51	23.42	0.79	*Hedysarum Multijugum Maxim.*
MOL000441	LUPENONE	424.78	11.66	0.78	*Hedysarum Multijugum Maxim.*
MOL000442	1,7-Dihydroxy-3,9-dimethoxy pterocarpene	314.31	39.05	0.48	*Hedysarum Multijugum Maxim.*
MOL000054	L-	174.24	47.64	0.03	*Hedysarum Multijugum Maxim.*
MOL000061	Prolinum	115.15	77.57	0.01	*Hedysarum Multijugum Maxim.*
MOL000069	palmitic acid	256.48	19.3	0.1	*Hedysarum Multijugum Maxim.*
MOL000098	quercetin	302.25	46.43	0.28	*Hedysarum Multijugum Maxim.*
MOL001006	poriferasta-7,22E-dien-3beta-ol	412.77	42.98	0.76	*Codonopsis Radix*
MOL001160	2-methoxyfuranodiene	246.38	53.58	0.13	*Codonopsis Radix*
MOL000125	(-)-alpha-Pinene	136.26	46.25	0.05	*Codonopsis Radix*
MOL001309	6-methylolpyridin-3-ol	125.14	47.53	0.02	*Codonopsis Radix*
MOL000131	EIC	280.5	41.9	0.14	*Codonopsis Radix*
MOL001314	Azelex	188.25	16.9	0.04	*Codonopsis Radix*
MOL001392	Methyl myristate	242.45	19.68	0.08	*Codonopsis Radix*
MOL001393	myristic acid	228.42	21.18	0.07	*Codonopsis Radix*
MOL001394	Oktadekan	254.56	9.81	0.09	*Codonopsis Radix*
MOL001396	PENTADECYLIC ACID	242.45	20.18	0.08	*Codonopsis Radix*
MOL001399	TWT	310.68	8.37	0.18	*Codonopsis Radix*
MOL001619	UPL	268.59	8.52	0.11	*Codonopsis Radix*
MOL001620	Pentadecene	210.45	17.72	0.05	*Codonopsis Radix*
MOL001641	METHYL LINOLEATE	294.53	41.93	0.17	*Codonopsis Radix*
MOL001644	Dodecanal	184.36	21.52	0.03	*Codonopsis Radix*
MOL000018	(+/-)-Isoborneol	154.28	86.98	0.05	*Codonopsis Radix*
MOL001817	Methyl stearate	298.57	16.8	0.16	*Codonopsis Radix*
MOL001819	METHYL PENTADECANOATE	256.48	18.82	0.1	*Codonopsis Radix*
MOL001887	SRT	150.1	45.27	0.02	*Codonopsis Radix*
MOL002046	hexanoic acid	116.18	73.08	0.01	*Codonopsis Radix*
MOL002140	Perlolyrine	264.3	65.95	0.27	*Codonopsis Radix*
MOL002307	20-Hexadecanoylingenol	586.94	28.2	0.68	*Codonopsis Radix*
MOL002521	beta-Curcumene	204.39	4.48	0.06	*Codonopsis Radix*
MOL002526	3691-11-0	204.39	23.66	0.07	*Codonopsis Radix*
MOL002579	capsaicin	305.46	10.31	0.2	*Codonopsis Radix*
MOL000261	Myristicin	192.23	17.99	0.07	*Codonopsis Radix*
MOL000027	alpha-Curcumene	202.37	4.68	0.06	*Codonopsis Radix*
MOL002879	Diop	390.62	43.59	0.39	*Codonopsis Radix*
MOL002943	BuOH	74.14	22.02	0	*Codonopsis Radix*
MOL000303	caprylic acid	144.24	16.4	0.02	*Codonopsis Radix*
MOL003035	stigmasterol-β-glucoside	574.93	2.4	0.63	*Codonopsis Radix*
MOL003036	ZINC03978781	412.77	43.83	0.76	*Codonopsis Radix*
MOL000305	lauric acid	200.36	23.59	0.04	*Codonopsis Radix*
MOL003050	nonanoic acid	158.27	40.51	0.02	*Codonopsis Radix*
MOL003177	Syringaldehyde	182.19	67.06	0.05	*Codonopsis Radix*
MOL003304	Hentriacontan	436.95	8.07	0.51	*Codonopsis Radix*
MOL000449	Stigmasterol	412.77	43.83	0.76	*Codonopsis Radix*
MOL000347	Syrigin	372.41	14.64	0.32	*Codonopsis Radix*
MOL003487	D-Friedoolean-14-en-3-one	424.78	12.9	0.77	*Codonopsis Radix*
MOL003509	Nonanol	144.29	33.19	0.01	*Codonopsis Radix*
MOL000365	syringaresinol	418.48	3.29	0.72	*Codonopsis Radix*

MOL003766	Shekanin	462.44	25.1	0.79	*Codonopsis Radix*
MOL003767	tectorigenin	300.28	28.41	0.27	*Codonopsis Radix*
MOL003895	5-Methoxymethyl furfural	140.15	28.23	0.02	*Codonopsis Radix*
MOL003896	7-Methoxy-2-methyl isoflavone	266.31	42.56	0.2	*Codonopsis Radix*
MOL000394	choline	104.2	0.47	0.01	*Codonopsis Radix*
MOL000421	nicotinic acid	123.12	47.65	0.02	*Codonopsis Radix*
MOL004355	Spinasterol	412.77	42.98	0.76	*Codonopsis Radix*
MOL000044	atractylenolideII	232.35	47.5	0.15	*Codonopsis Radix*
MOL004492	Chrysanthemaxanthin	584.96	38.72	0.58	*Codonopsis Radix*
MOL004498	12-O-Nicotinoylisolineolone	469.63	20.7	0.83	*Codonopsis Radix*
MOL000045	atractylenolide iii	248.35	68.11	0.17	*Codonopsis Radix*
MOL004582	Methyl naphthalene	142.21	39.01	0.04	*Codonopsis Radix*
MOL004623	Encecalin	232.3	21.36	0.11	*Codonopsis Radix*
MOL004652	(2R,3R,4S,5S,6R)-2-[[(3S,5S,9R,10S,13R,14R,17R)-17-[(E,2R,5S)-5-ethyl-6-methylhept-3-en-2-yl]-10,13-dimethyl-2,3,4,5,6,9,11,12,14,15,16,17-dodecahydro-1H-cyclopenta[a]phenanthren-3-yl]oxy]-6-(hydroxymethyl)oxane-3,4,5-triol	574.93	21.2	0.63	*Codonopsis Radix*
MOL004664	heptanoic acid	130.21	13.38	0.01	*Codonopsis Radix*
MOL000508	Friedelin	426.8	29.16	0.76	*Codonopsis Radix*
MOL005270	n-Heptadecanol	256.53	12.97	0.09	*Codonopsis Radix*
MOL005302	7-(beta-Xylosyl)cephalomannine	962.15	27.33	0.17	*Codonopsis Radix*
MOL005304	7alpha-L-Rhamnosyl-6-methoxylutcolin	462.44	15.03	0.79	*Codonopsis Radix*
MOL005321	Frutinone A	264.24	65.9	0.34	*Codonopsis Radix*
MOL005482	FOA	112.09	35.66	0.02	*Codonopsis Radix*
MOL000006	luteolin	286.25	36.16	0.25	*Codonopsis Radix*
MOL000617	(14S)-14-methylpalmitic acid	270.51	23.12	0.11	*Codonopsis Radix*
MOL000628	darutoside	574.93	21.32	0.63	*Codonopsis Radix*
MOL006322	Friedoolean-14-en-3-yl acetate	468.84	9.59	0.74	*Codonopsis Radix*
MOL006554	Taraxerol	426.8	38.4	0.77	*Codonopsis Radix*
MOL000667	1-hexanol	102.2	22.04	0.01	*Codonopsis Radix*
MOL006774	stigmast-7-enol	414.79	37.42	0.75	*Codonopsis Radix*
MOL006844	Norharman	168.21	18.88	0.08	*Codonopsis Radix*
MOL000069	palmitic acid	256.48	19.3	0.1	*Codonopsis Radix*
MOL006988	NSC405997	342.68	12.59	0.26	*Codonopsis Radix*
MOL007059	3-beta-Hydroxymethyllenetanshiquinone	294.32	32.16	0.41	*Codonopsis Radix*
MOL000721	Nonadienal	138.23	19.03	0.02	*Codonopsis Radix*
MOL000748	HMF	126.12	45.07	0.02	*Codonopsis Radix*
MOL007514	methyl icosa-11,14-dienoate	322.59	39.67	0.23	*Codonopsis Radix*
MOL000008	apigenin	270.25	23.06	0.21	*Codonopsis Radix*
MOL008142	Ricinin	164.18	26.26	0.04	*Codonopsis Radix*
MOL008284	BUA	88.12	21.62	0	*Codonopsis Radix*
MOL000835	EA-fructofuranoside	208.24	47.33	0.06	*Codonopsis Radix*
MOL008375	(1R)-2,3,4,9-tetrahydro-1H-$b-carboline-1-carboxylic acid	216.26	52.9	0.13	*Codonopsis Radix*
MOL008376	13-Methyl pentadecanoic acid	256.48	24.14	0.1	*Codonopsis Radix*
MOL008377	Galuteolin	448.41	2.7	0.79	*Codonopsis Radix*
MOL008378	o-(o-Methoxyphenoxy)phenol	216.25	50.75	0.09	*Codonopsis Radix*

MOL008379	tangshenoside I	678.71	4.93	0.47	*Codonopsis Radix*
MOL008380	tangshenoside I_qt	354.39	23.58	0.3	*Codonopsis Radix*
MOL008381	tangshenoside III	726.8	8.75	0.46	*Codonopsis Radix*
MOL008382	tangshenoside III_qt	402.48	4.98	0.45	*Codonopsis Radix*
MOL008383	tangshenoside IV	1033.1	3.01	0.15	*Codonopsis Radix*
MOL008384	tangshenoside IV_qt	546.62	4.28	0.67	*Codonopsis Radix*
MOL008385	T-BUTYLBENZENE	134.24	53.06	0.02	*Codonopsis Radix*
MOL008386	2,6-NONADIENOL	140.25	23.77	0.02	*Codonopsis Radix*
MOL008387	3-METHYLCARBAZOLE	181.25	20.14	0.09	*Codonopsis Radix*
MOL008388	L-Sulforaphane	177.32	56.97	0.02	*Codonopsis Radix*
MOL008389	4-Phenylbicyclo[2,2,2]octan-1-ol	202.32	25.07	0.09	*Codonopsis Radix*
MOL008390	5-Mpe-bis(hobz)phenol	438.55	11.33	0.66	*Codonopsis Radix*
MOL008391	5alpha-Stigmastan-3,6-dione	428.77	33.12	0.79	*Codonopsis Radix*
MOL008392	6,6'-Dimethoxygossypol	550.7	8.93	0.86	*Codonopsis Radix*
MOL008393	7-(beta-Xylosyl)cephalomannine_qt	830.02	38.33	0.29	*Codonopsis Radix*
MOL008394	Butylcyclohexane	140.3	35.68	0.02	*Codonopsis Radix*
MOL008395	Codonopsine	267.36	45.83	0.13	*Codonopsis Radix*
MOL008396	Coelogin	300.33	21.68	0.42	*Codonopsis Radix*
MOL008397	Daturilin	436.64	50.37	0.77	*Codonopsis Radix*
MOL008398	Ethyl-p-digallate	350.3	2.27	0.31	*Codonopsis Radix*
MOL008399	fritillaziebinol	324.98	15.01	0.34	*Codonopsis Radix*
MOL008400	glycitein	284.28	50.48	0.24	*Codonopsis Radix*
MOL008401	Henicosanoic acid	326.63	16.14	0.23	*Codonopsis Radix*
MOL008402	BHG	264.36	15.6	0.11	*Codonopsis Radix*
MOL008403	Hexyl-beta-D-glucopyranosyl-(1-2)-beta-D-glucopyranoside	426.52	6.51	0.44	*Codonopsis Radix*
MOL008404	(2S)-2-ammonio-4-[(R)-methylsulfinyl]butyrate	165.24	83.52	0.02	*Codonopsis Radix*
MOL008405	1-Peroxyferolide	338.39	17.38	0.35	*Codonopsis Radix*
MOL008406	Spinoside A	716.95	39.97	0.4	*Codonopsis Radix*
MOL008407	(8S,9S,10R,13R,14S,17R)-17-[(E,2R,5S)-5-ethyl-6-methylhept-3-en-2-yl]-10,13-dimethyl-1,2,4,7,8,9,11,12,14,15,16,17-dodecahydrocyclopenta[a]phenanthren-3-one	410.75	45.4	0.76	*Codonopsis Radix*
MOL008408	Stigmasteryl ferulate	588.95	24.53	0.55	*Codonopsis Radix*
MOL008409	Tangshenoside II	372.41	19.5	0.32	*Codonopsis Radix*
MOL008410	Tangshenoside II_qt	210.25	51.72	0.06	*Codonopsis Radix*
MOL008411	11-Hydroxyrankinidine	356.46	40	0.66	*Codonopsis Radix*
MOL008412	alpha-Stigmasta-7,22-dien-3-one	410.75	11.42	0.76	*Codonopsis Radix*
MOL008413	Codopiloic acid	127.11	57.5	0.02	*Codonopsis Radix*
MOL008414	delta22-Stigmasterol	414.79	7.04	0.76	*Codonopsis Radix*
MOL008415	delta7-Stigmastenone-3	412.77	9.69	0.76	*Codonopsis Radix*
MOL008416	delta7-stigmastenol-belta-D-glucopyranoside	578.97	19.58	0.62	*Codonopsis Radix*
MOL008417	ethyl-β-D-fructofuranoside	320.48	33.84	0.15	*Codonopsis Radix*
MOL000860	stearic acid	284.54	17.83	0.14	*Codonopsis Radix*
MOL000867	Heptadekan	240.53	8.64	0.07	*Codonopsis Radix*
MOL000869	Henicosane	296.65	8.41	0.15	*Codonopsis Radix*
MOL000879	methyl palmitate	270.51	18.09	0.12	*Codonopsis Radix*
MOL000885	Dodekan	170.38	17.74	0.02	*Codonopsis Radix*
MOL000890	(+)-alpha-Curcumene	202.37	26.56	0.06	*Codonopsis Radix*

MOL000899	Furanodiene	216.35	45.11	0.1	*Codonopsis Radix*
MOL000009	luteolin-7-o-glucoside	448.41	7.29	0.78	*Codonopsis Radix*
MOL000905	()-beta-Pinene	136.26	44.77	0.05	*Codonopsis Radix*
MOL000095	delta 7-stigmastenol	416.81	25.32	0.75	*Codonopsis Radix*
MOL000998	2-(3,4-dihydroxyphenyl)-5,7-dihydroxy-3-[(2R,3R,4S,5S)-3,4,5-trihydroxytetrahydropyran-2-yl]oxy-chromone	434.38	4.05	0.7	*Codonopsis Radix*
MOL000018	(+/-)-Isoborneol	154.28	86.98	0.05	*Atractylodes Macrocephala Koidz.*
MOL000019	D-Camphene	136.26	34.98	0.04	*Atractylodes Macrocephala Koidz.*
MOL000020	12-senecioyl-2E,8E,10E-atractylentriol	312.39	62.4	0.22	*Atractylodes Macrocephala Koidz.*
MOL000021	14-acetyl-12-senecioyl-2E,8E,10E-atractylentriol	355.44	60.31	0.31	*Atractylodes Macrocephala Koidz.*
MOL000022	14-acetyl-12-senecioyl-2E,8Z,10E-atractylentriol	356.45	63.37	0.3	*Atractylodes Macrocephala Koidz.*
MOL000023	Hemo-sol	136.26	39.84	0.02	*Atractylodes Macrocephala Koidz.*
MOL000024	alpha-humulene	204.39	22.98	0.06	*Atractylodes Macrocephala Koidz.*
MOL000025	α-Longipinene	204.39	53.26	0.12	*Atractylodes Macrocephala Koidz.*
MOL000026	stigmast-22E-en-3beta-ol	414.79	10.39	0.75	*Atractylodes Macrocephala Koidz.*
MOL000027	alpha-Curcumene	202.37	4.68	0.06	*Atractylodes Macrocephala Koidz.*
MOL000028	α-Amyrin	426.8	39.51	0.76	*Atractylodes Macrocephala Koidz.*
MOL000029	beta-Humulene	204.39	26.87	0.06	*Atractylodes Macrocephala Koidz.*
MOL000030	(1R)-2-methyl-1-phenylprop-2-en-1-ol	148.22	75.1	0.03	*Atractylodes Macrocephala Koidz.*
MOL000031	(3S)-3-[(1R)-1,5-dimethylhex-4-enyl]-6-methylenecyclohexene	204.39	19.86	0.06	*Atractylodes Macrocephala Koidz.*
MOL000032	beta-Eudesmol	222.41	26.09	0.1	*Atractylodes Macrocephala Koidz.*
MOL000033	(3S,8S,9S,10R,13R,14S,17R)-10,13-dimethyl-17-[(2R,5S)-5-propan-2-yloctan-2-yl]-2,3,4,7,8,9,11,12,14,15,16,17-dodecahydro-1H-cyclopenta[a]phenanthren-3-ol	428.82	36.23	0.78	*Atractylodes Macrocephala Koidz.*
MOL000034	2-[(1R,3S,4S)-3-isopropenyl-4-methyl-4-vinylcyclohexyl]propan-2-ol	222.41	19.03	0.07	*Atractylodes Macrocephala Koidz.*
MOL000035	beta-Selinene	204.39	24.39	0.08	*Atractylodes Macrocephala Koidz.*
MOL000036	beta-caryophyllene	204.39	29.7	0.09	*Atractylodes Macrocephala Koidz.*
MOL000037	γ-elemene	204.39	23.79	0.06	*Atractylodes Macrocephala Koidz.*

MOL000038	Akridin	179.23	33.71	0.1	*Atractylodes Macrocephala Koidz.*
MOL000039	(1S,2R,4R)-Neoiso-dihydrocarveol	154.28	52.4	0.03	*Atractylodes Macrocephala Koidz.*
MOL000040	Scopoletol	192.18	27.77	0.08	*Atractylodes Macrocephala Koidz.*
MOL000041	PHA	165.21	41.62	0.04	*Atractylodes Macrocephala Koidz.*
MOL000042	LPG	89.11	87.69	0.01	*Atractylodes Macrocephala Koidz.*
MOL000043	atractylenolide i	230.33	37.37	0.15	*Atractylodes Macrocephala Koidz.*
MOL000044	atractylenolideII	232.35	47.5	0.15	*Atractylodes Macrocephala Koidz.*
MOL000045	atractylenolide iii	248.35	68.11	0.17	*Atractylodes Macrocephala Koidz.*
MOL000046	atractylone	216.35	41.1	0.13	*Atractylodes Macrocephala Koidz.*
MOL000047	juniper camphor	222.41	33.3	0.1	*Atractylodes Macrocephala Koidz.*
MOL000048	(5E,9Z)-3,6,10-trimethyl-4,7,8,11-tetrahydrocyclodeca[b]furan	216.35	43.17	0.1	*Atractylodes Macrocephala Koidz.*
MOL000049	3β-acetoxyatractylone	274.39	54.07	0.22	*Atractylodes Macrocephala Koidz.*
MOL000050	GLY	75.08	48.74	0	*Atractylodes Macrocephala Koidz.*
MOL000051	Polymannose	180.18	1.76	0.03	*Atractylodes Macrocephala Koidz.*
MOL000052	Gulutamine	147.15	6.66	0.02	*Atractylodes Macrocephala Koidz.*
MOL000053	Methose	180.18	1.68	0.03	*Atractylodes Macrocephala Koidz.*
MOL000054	L-	174.24	47.64	0.03	*Atractylodes Macrocephala Koidz.*
MOL000055	L-Lysin	146.22	29.33	0.02	*Atractylodes Macrocephala Koidz.*
MOL000056	DTY	181.21	57.55	0.05	*Atractylodes Macrocephala Koidz.*
MOL000057	DIBP	278.38	49.63	0.13	*Atractylodes Macrocephala Koidz.*
MOL000058	2-[(2R,5S,6S)-6,10-dimethylspiro[4.5]dec-9-en-2-yl]propan-2-ol	222.41	38.59	0.09	*Atractylodes Macrocephala Koidz.*
MOL000059	uridine	244.23	10.49	0.11	*Atractylodes Macrocephala Koidz.*
MOL000060	selina-4(14),7(11)-dien-8-one	218.37	32.31	0.1	*Atractylodes Macrocephala Koidz.*
MOL000061	Prolinum	115.15	77.57	0.01	*Atractylodes Macrocephala Koidz.*

MOL000062	biatractylolide	462.68	17.45	0.81	*Atractylodes Macrocephala Koidz.*
MOL000063	ATRACTYLODES MACROCEPHALA	462.68	14.6	0.81	*Atractylodes Macrocephala Koidz.*
MOL000064	D-Serin	105.11	83.59	0.01	*Atractylodes Macrocephala Koidz.*
MOL000065	ASI	133.12	79.74	0.02	*Atractylodes Macrocephala Koidz.*
MOL000066	alloaromadedrene	204.39	53.46	0.1	*Atractylodes Macrocephala Koidz.*
MOL000067	L-Valin	117.17	53.33	0.01	*Atractylodes Macrocephala Koidz.*
MOL000068	L-Ile	131.2	59.05	0.02	*Atractylodes Macrocephala Koidz.*
MOL000069	palmitic acid	256.48	19.3	0.1	*Atractylodes Macrocephala Koidz.*
MOL000070	Ethyl pivaloylacetate	172.25	40.52	0.03	*Atractylodes Macrocephala Koidz.*
MOL000071	Istidina	155.18	53.18	0.03	*Atractylodes Macrocephala Koidz.*
MOL000072	8β-ethoxy atractylenolide Ⅲ	276.41	35.95	0.21	*Atractylodes Macrocephala Koidz.*
MOL000136	Dioscoreside C	1061.36	10.32	0.03	*Rhizoma Dioscoreae*
MOL001559	piperlonguminine	273.36	30.71	0.18	*Rhizoma Dioscoreae*
MOL001618	Pellitorin	223.4	23.81	0.06	*Rhizoma Dioscoreae*
MOL001736	(-)-taxifolin	304.27	60.51	0.27	*Rhizoma Dioscoreae*
MOL002442	Cholesteryl ferulate	562.91	22.43	0.63	*Rhizoma Dioscoreae*
MOL000309	denudatin,a	340.4	6.93	0.44	*Rhizoma Dioscoreae*
MOL000310	Denudatin B	356.45	61.47	0.38	*Rhizoma Dioscoreae*
MOL000322	Kadsurenone	356.45	54.72	0.38	*Rhizoma Dioscoreae*
MOL000388	gamma-aminobutyric acid	103.14	24.09	0.01	*Rhizoma Dioscoreae*
MOL000394	choline	104.2	0.47	0.01	*Rhizoma Dioscoreae*
MOL003955	D-Cystine	240.34	39.58	0.05	*Rhizoma Dioscoreae*
MOL003969	L-Serin	105.11	98.47	0.01	*Rhizoma Dioscoreae*
MOL003971	Threonin	119.14	73.52	0.01	*Rhizoma Dioscoreae*
MOL000041	PHA	165.21	41.62	0.04	*Rhizoma Dioscoreae*
MOL000042	LPG	89.11	87.69	0.01	*Rhizoma Dioscoreae*
MOL004668	GLB	180.18	47.71	0.04	*Rhizoma Dioscoreae*
MOL000050	GLY	75.08	48.74	0	*Rhizoma Dioscoreae*
MOL000052	Gulutamine	147.15	6.66	0.02	*Rhizoma Dioscoreae*
MOL000053	Methose	180.18	1.68	0.03	*Rhizoma Dioscoreae*
MOL005367	GUP	180.18	43.04	0.04	*Rhizoma Dioscoreae*
MOL000054	L-	174.24	47.64	0.03	*Rhizoma Dioscoreae*
MOL005427	(24S)-belta-Methyl cholest-8(14)-enol	400.76	7.33	0.72	*Rhizoma Dioscoreae*
MOL005428	(24S)-belta-Methyl cholestanol	402.78	25.81	0.71	*Rhizoma Dioscoreae*
MOL005429	hancinol	372.5	64.01	0.37	*Rhizoma Dioscoreae*
MOL005430	hancinone C	400.51	59.05	0.39	*Rhizoma Dioscoreae*
MOL005431	Crotepoxide	362.36	27.02	0.44	*Rhizoma Dioscoreae*

MOL005432	ABK	264.35	63.67	0.13	*Rhizoma Dioscoreae*
MOL005433	(3S,8S,9S,10R,13R,14S,17R)-17-[(2R,5S)-5-ethyl-6-methylhept-6-en-2-yl]-10,13-dimethyl-2,3,4,7,8,9,11,12,14,15,16,17-dodecahydro-1H-cyclopenta[a]phenanthren-3-ol	412.77	7.56	0.76	*Rhizoma Dioscoreae*
MOL005434	24-Methylcholest-5-enyl-3belta-O-glucopyranoside	562.92	20.49	0.67	*Rhizoma Dioscoreae*
MOL005435	24-Methylcholest-5-enyl-3belta-O-glucopyranoside_qt	400.76	37.58	0.72	*Rhizoma Dioscoreae*
MOL005436	24-Methylcholesta-5,7,22-trien-ebelta-ol	396.72	18.22	0.72	*Rhizoma Dioscoreae*
MOL005437	(2S,3S,3aR)-3a-allyl-2-(1,3-benzodioxol-5-yl)-5-methoxy-3-methyl-2,3-dihydrobenzofuran-6-one	340.4	3.23	0.43	*Rhizoma Dioscoreae*
MOL005438	campesterol	400.76	37.58	0.71	*Rhizoma Dioscoreae*
MOL005439	Ostreasterol	398.74	13.62	0.72	*Rhizoma Dioscoreae*
MOL005440	Isofucosterol	412.77	43.78	0.76	*Rhizoma Dioscoreae*
MOL005441	BGC	180.18	48.09	0.04	*Rhizoma Dioscoreae*
MOL005442	LDP	153.2	74.4	0.03	*Rhizoma Dioscoreae*
MOL005443	Batatasin I	284.33	23.7	0.27	*Rhizoma Dioscoreae*
MOL005444	3-[2-(3-hydroxyphenyl)ethyl]-5-methoxyphenol	244.31	3.75	0.12	*Rhizoma Dioscoreae*
MOL000298	ergosterol	396.72	14.29	0.72	*Rhizoma Dioscoreae*
MOL005446	Batatasin IV	244.31	82.76	0.12	*Rhizoma Dioscoreae*
MOL005447	L-Xyl	150.15	40.44	0.02	*Rhizoma Dioscoreae*
MOL005448	Leucinum	131.2	72.92	0.01	*Rhizoma Dioscoreae*
MOL005449	h-Met-h	149.24	70.87	0.01	*Rhizoma Dioscoreae*
MOL005450	cholestanol	388.75	25.63	0.67	*Rhizoma Dioscoreae*
MOL005451	holest-7-enol	386.73	11.81	0.68	*Rhizoma Dioscoreae*
MOL000449	Stigmasterol	412.77	43.83	0.76	*Rhizoma Dioscoreae*
MOL005453	phytic acid	660.06	4.78	0.5	*Rhizoma Dioscoreae*
MOL005454	(S)-Allantoin	158.14	106.68	0.03	*Rhizoma Dioscoreae*
MOL005455	(-)-ABA	264.35	31.79	0.13	*Rhizoma Dioscoreae*
MOL005456	Deltoside	1063.33	10.07	0.01	*Rhizoma Dioscoreae*
MOL005457	Deltoside_qt	430.69	10.36	0.78	*Rhizoma Dioscoreae*
MOL005458	Dioscoreside C_qt	444.72	36.38	0.87	*Rhizoma Dioscoreae*
MOL005459	Diosgenin-3-O-beta-D-glucopyranoside	576.85	7.94	0.29	*Rhizoma Dioscoreae*
MOL000546	diosgenin	414.69	80.88	0.81	*Rhizoma Dioscoreae*
MOL005460	ophipogonin D_qt	414.69	15.84	0.81	*Rhizoma Dioscoreae*
MOL005461	Doradexanthin	584.96	38.16	0.54	*Rhizoma Dioscoreae*
MOL005462	Methylcimicifugoside	688.94	46.31	0.09	*Rhizoma Dioscoreae*
MOL005463	Methylcimicifugoside_qt	556.81	31.69	0.24	*Rhizoma Dioscoreae*
MOL005464	(3S,4aS,4bS,8R,8aS,10aR)-3-hydroxy-1,1,4a,8-tetramethyl-7-vinyl-3,4,4b,8,8a,9,10,10a-octahydrophenanthrene-2,5-dione	316.48	24.48	0.31	*Rhizoma Dioscoreae*
MOL005465	AIDS180907	394.45	45.33	0.77	*Rhizoma Dioscoreae*
MOL005466	1,3,6-trihydroxy-8-(3-hydroxy-3-methyl-butyl)-7-methoxy-2-(3-methylbut-2-enyl)xanthone	428.52	0.98	0.68	*Rhizoma Dioscoreae*
MOL000055	L-Lysin	146.22	29.33	0.02	*Rhizoma Dioscoreae*
MOL000056	DTY	181.21	57.55	0.05	*Rhizoma Dioscoreae*
MOL000061	Prolinum	115.15	77.57	0.01	*Rhizoma Dioscoreae*

MOL000628	darutoside	574.93	21.32	0.63	*Rhizoma Dioscoreae*
MOL000065	ASI	133.12	79.74	0.02	*Rhizoma Dioscoreae*
MOL000067	L-Valin	117.17	53.33	0.01	*Rhizoma Dioscoreae*
MOL000068	L-Ile	131.2	59.05	0.02	*Rhizoma Dioscoreae*
MOL000071	Istidina	155.18	53.18	0.03	*Rhizoma Dioscoreae*
MOL000953	CLR	386.73	37.87	0.68	*Rhizoma Dioscoreae*
MOL000273	(2R)-2-[(3S,5R,10S,13R,14R,16R,17R)-3,16-dihydroxy-4,4,10,13,14-pentamethyl-2,3,5,6,12,15,16,17-octahydro-1H-cyclopenta[a]phenanthren-17-yl]-6-methylhept-5-enoic acid	470.76	30.93	0.81	*Poria Cocos(Schw.) Wolf.*
MOL000274	3β-hydroxylanosta-7,9(11),24-trien-21-oic acid	454.76	24.92	0.8	*Poria Cocos(Schw.) Wolf.*
MOL000275	trametenolic acid	456.78	38.71	0.8	*Poria Cocos(Schw.) Wolf.*
MOL000276	7,9(11)-dehydropachymic acid	526.83	35.11	0.81	*Poria Cocos(Schw.) Wolf.*
MOL000277	tumulosic acid	486.81	15.95	0.81	*Poria Cocos(Schw.) Wolf.*
MOL000278	Beta-Glucan	516.56	0.73	0.7	*Poria Cocos(Schw.) Wolf.*
MOL000279	Cerevisterol	430.74	37.96	0.77	*Poria Cocos(Schw.) Wolf.*
MOL000280	(2R)-2-[(3S,5R,10S,13R,14R,16R,17R)-3,16-dihydroxy-4,4,10,13,14-pentamethyl-2,3,5,6,12,15,16,17-octahydro-1H-cyclopenta[a]phenanthren-17-yl]-5-isopropyl-hex-5-enoic acid	484.79	31.07	0.82	*Poria Cocos(Schw.) Wolf.*
MOL000281	Dimethyl L-malate	162.16	8.59	0.03	*Poria Cocos(Schw.) Wolf.*
MOL000282	ergosta-7,22E-dien-3beta-ol	398.74	43.51	0.72	*Poria Cocos(Schw.) Wolf.*
MOL000283	Ergosterol peroxide	430.74	40.36	0.81	*Poria Cocos(Schw.) Wolf.*
MOL000284	L-uridine	244.23	23.4	0.11	*Poria Cocos(Schw.) Wolf.*
MOL000285	(2R)-2-[(5R,10S,13R,14R,16R,17R)-16-hydroxy-3-keto-4,4,10,13,14-pentamethyl-1,2,5,6,12,15,16,17-octahydrocyclopenta[a]phenanthren-17-yl]-5-isopropyl-hex-5-enoic acid	482.77	38.26	0.82	*Poria Cocos(Schw.) Wolf.*
MOL000286	β-amyrin acetate	468.84	9.11	0.74	*Poria Cocos(Schw.) Wolf.*
MOL000287	3beta-Hydroxy-24-methylene-8-lanostene-21-oic acid	470.81	38.7	0.81	*Poria Cocos(Schw.) Wolf.*
MOL000288	pachyman	500.56	0.45	0.68	*Poria Cocos(Schw.) Wolf.*
MOL000289	pachymic acid	528.85	33.63	0.81	*Poria Cocos(Schw.) Wolf.*
MOL000290	Poricoic acid A	498.77	30.61	0.76	*Poria Cocos(Schw.) Wolf.*
MOL000291	Poricoic acid B	484.74	30.52	0.75	*Poria Cocos(Schw.) Wolf.*
MOL000292	poricoic acid C	482.77	38.15	0.75	*Poria Cocos(Schw.) Wolf.*
MOL000293	poricoic acid D	514.77	22.38	0.78	*Poria Cocos(Schw.) Wolf.*
MOL000294	poricoic acid DM	528.8	29.32	0.78	*Poria Cocos(Schw.) Wolf.*
MOL000295	alexandrin	576.95	20.63	0.63	*Poria Cocos(Schw.) Wolf.*
MOL000296	hederagenin	414.79	36.91	0.75	*Poria Cocos(Schw.) Wolf.*
MOL000297	Tumulosic acid	486.81	29.88	0.81	*Poria Cocos(Schw.) Wolf.*
MOL000298	ergosterol	396.72	14.29	0.72	*Poria Cocos(Schw.) Wolf.*
MOL000299	Trimethyl citrate	234.23	67.61	0.07	*Poria Cocos(Schw.) Wolf.*
MOL000300	dehydroeburicoic acid	453.75	44.17	0.83	*Poria Cocos(Schw.) Wolf.*
MOL000301	2-lauroleic acid	198.34	31.42	0.04	*Poria Cocos(Schw.) Wolf.*
MOL000302	Undekansaeure	186.33	30.14	0.03	*Poria Cocos(Schw.) Wolf.*
MOL000303	caprylic acid	144.24	16.4	0.02	*Poria Cocos(Schw.) Wolf.*

MOL000304	Ethyl glucoside	208.24	15.21	0.06	*Poria Cocos(Schw.) Wolf.*
MOL000305	lauric acid	200.36	23.59	0.04	*Poria Cocos(Schw.) Wolf.*
MOL000069	palmitic acid	256.48	19.3	0.1	*Poria Cocos(Schw.) Wolf.*
MOL000105	protocatechuic acid	154.13	25.37	0.04	*Arum Ternatum Thunb.*
MOL000114	vanillic acid	168.16	35.47	0.04	*Arum Ternatum Thunb.*
MOL000127	Neral	152.26	19.48	0.02	*Arum Ternatum Thunb.*
MOL001281	L-alpha-Palmitin	330.57	26.66	0.22	*Arum Ternatum Thunb.*
MOL000131	EIC	280.5	41.9	0.14	*Arum Ternatum Thunb.*
MOL001385	9-Oxononanoic acid	172.25	19.6	0.03	*Arum Ternatum Thunb.*
MOL001396	PENTADECYLIC ACID	242.45	20.18	0.08	*Arum Ternatum Thunb.*
MOL001452	protocatechualdehyde	138.13	38.35	0.03	*Arum Ternatum Thunb.*
MOL001492	Ethyl icosanoate	340.66	16.67	0.25	*Arum Ternatum Thunb.*
MOL000172	Furol	96.09	34.35	0.01	*Arum Ternatum Thunb.*
MOL001729	Crysophanol	254.25	18.64	0.21	*Arum Ternatum Thunb.*
MOL001739	zoomaric acid	254.46	35.78	0.1	*Arum Ternatum Thunb.*
MOL001744	uracil	112.1	42.53	0.02	*Arum Ternatum Thunb.*
MOL001755	24-Ethylcholest-4-en-3-one	412.77	36.08	0.76	*Arum Ternatum Thunb.*
MOL001757	GUN	151.15	42.45	0.04	*Arum Ternatum Thunb.*
MOL001788	adenine	135.15	62.81	0.03	*Arum Ternatum Thunb.*
MOL001816	Amide HPL	255.5	19.79	0.1	*Arum Ternatum Thunb.*
MOL001818	Methyl palmitelaidate	268.49	34.61	0.12	*Arum Ternatum Thunb.*
MOL001831	HX	136.13	52.29	0.04	*Arum Ternatum Thunb.*
MOL000223	caffeic acid	180.17	25.76	0.05	*Arum Ternatum Thunb.*
MOL002254	Barolub	807.49	16.29	0.22	*Arum Ternatum Thunb.*
MOL002495	6-shogaol	276.41	31	0.14	*Arum Ternatum Thunb.*
MOL002670	Cavidine	353.45	35.64	0.81	*Arum Ternatum Thunb.*
MOL002714	baicalein	270.25	33.52	0.21	*Arum Ternatum Thunb.*
MOL002776	Baicalin	446.39	40.12	0.75	*Arum Ternatum Thunb.*
MOL000346	succinic acid	118.1	29.62	0.01	*Arum Ternatum Thunb.*
MOL000357	Sitogluside	576.95	20.63	0.62	*Arum Ternatum Thunb.*
MOL000358	beta-sitosterol	414.79	36.91	0.75	*Arum Ternatum Thunb.*
MOL000384	DL-Glucuronic acid	194.16	3.35	0.04	*Arum Ternatum Thunb.*
MOL003870	gynesine	137.15	60.07	0.03	*Arum Ternatum Thunb.*
MOL000388	gamma-aminobutyric acid	103.14	24.09	0.01	*Arum Ternatum Thunb.*
MOL000389	FERULIC ACID (CIS)	194.2	54.97	0.06	*Arum Ternatum Thunb.*
MOL000394	choline	104.2	0.47	0.01	*Arum Ternatum Thunb.*
MOL003969	L-Serin	105.11	98.47	0.01	*Arum Ternatum Thunb.*
MOL000397	cis-p-Coumarate	164.17	45.98	0.04	*Arum Ternatum Thunb.*
MOL003971	Threonin	119.14	73.52	0.01	*Arum Ternatum Thunb.*
MOL000399	Docosanoate	340.66	15.69	0.26	*Arum Ternatum Thunb.*
MOL000432	linolenic acid	278.48	45.01	0.15	*Arum Ternatum Thunb.*
MOL004481	(2S)-2-amino-3-[(2R)-2-amino-3-hydroxy-3-oxopropyl]disulfanylpropanoic acid	240.34	73.59	0.05	*Arum Ternatum Thunb.*
MOL000449	Stigmasterol	412.77	43.83	0.76	*Arum Ternatum Thunb.*
MOL000458	campesterol	400.76	5.57	0.72	*Arum Ternatum Thunb.*
MOL004649	1-[(2R,3R,4S,5S)-3,4-dihydroxy-5-(hydroxymethyl)oxolan-2-yl]pyrimidine-2,4-dione	244.23	17.85	0.11	*Arum Ternatum Thunb.*
MOL004738	Spantol	179.24	2.42	0.04	*Arum Ternatum Thunb.*
MOL004739	DAL	89.11	85.17	0.01	*Arum Ternatum Thunb.*

MOL000475	anethole	148.22	32.49	0.03	*Arum Ternatum Thunb.*
MOL004796	soya-cerebroside i	714.16	3.86	0.37	*Arum Ternatum Thunb.*
MOL004797	soya-cerebroside i_qt	552	21.22	0.51	*Arum Ternatum Thunb.*
MOL000050	GLY	75.08	48.74	0	*Arum Ternatum Thunb.*
MOL005030	gondoic acid	310.58	30.7	0.2	*Arum Ternatum Thunb.*
MOL005125	ANN	152.16	29.69	0.03	*Arum Ternatum Thunb.*
MOL000513	3,4,5-trihydroxybenzoic acid	170.13	31.69	0.04	*Arum Ternatum Thunb.*
MOL000519	coniferin	314.41	31.11	0.32	*Arum Ternatum Thunb.*
MOL000052	Gulutamine	147.15	6.66	0.02	*Arum Ternatum Thunb.*
MOL000054	L-	174.24	47.64	0.03	*Arum Ternatum Thunb.*
MOL005448	Leucinum	131.2	72.92	0.01	*Arum Ternatum Thunb.*
MOL000055	L-Lysin	146.22	29.33	0.02	*Arum Ternatum Thunb.*
MOL000056	DTY	181.21	57.55	0.05	*Arum Ternatum Thunb.*
MOL000579	hydroquinone	110.12	29.26	0.02	*Arum Ternatum Thunb.*
MOL006240	THM	242.26	11.34	0.11	*Arum Ternatum Thunb.*
MOL000065	ASI	133.12	79.74	0.02	*Arum Ternatum Thunb.*
MOL006594	Eciphin	165.26	43.35	0.03	*Arum Ternatum Thunb.*
MOL006597	OMD	168.16	92.44	0.04	*Arum Ternatum Thunb.*
MOL000067	L-Valin	117.17	53.33	0.01	*Arum Ternatum Thunb.*
MOL000675	oleic acid	282.52	33.13	0.14	*Arum Ternatum Thunb.*
MOL000068	L-Ile	131.2	59.05	0.02	*Arum Ternatum Thunb.*
MOL006844	Norharman	168.21	18.88	0.08	*Arum Ternatum Thunb.*
MOL000069	palmitic acid	256.48	19.3	0.1	*Arum Ternatum Thunb.*
MOL006930	(+) isolariciresinol 9-o-β-d-glucopyranoside	522.6	3.83	0.84	*Arum Ternatum Thunb.*
MOL006931	isolariciresino	360.44	6.96	0.39	*Arum Ternatum Thunb.*
MOL006932	l-Pseudoephedrine	165.26	45.01	0.03	*Arum Ternatum Thunb.*
MOL006933	1,2,3,4,6-penta-o-galloyl-β-d-glucose	1092.83	3.01	0.13	*Arum Ternatum Thunb.*
MOL006934	WLN: Q5Q	104.17	24.8	0.01	*Arum Ternatum Thunb.*
MOL006935	Octylene	112.24	39.25	0.01	*Arum Ternatum Thunb.*
MOL006936	10,13-eicosadienoic	308.56	39.99	0.2	*Arum Ternatum Thunb.*
MOL006937	12,13-epoxy-9-hydroxynonadeca-7,10-dienoic acid	324.51	42.15	0.24	*Arum Ternatum Thunb.*
MOL006938	DUR	228.23	23.69	0.09	*Arum Ternatum Thunb.*
MOL006939	Methylpyrazine	94.13	27.1	0.01	*Arum Ternatum Thunb.*
MOL006940	D-2-Aminobutyrate	103.14	68.78	0.01	*Arum Ternatum Thunb.*
MOL006941	3-methyleicosa	296.65	10.18	0.15	*Arum Ternatum Thunb.*
MOL006942	N-(5-methylisoxazol-3-yl)acetamide	140.16	20.82	0.02	*Arum Ternatum Thunb.*
MOL006943	5,8-epidioxyergosta-6,22-dien-3-ol	428.72	22.71	0.82	*Arum Ternatum Thunb.*
MOL006944	8-Octadecenoic acid	282.52	33.13	0.14	*Arum Ternatum Thunb.*
MOL006945	9-Heptadecanol	256.53	14.24	0.09	*Arum Ternatum Thunb.*
MOL006946	adenosine	255.27	19.85	0.16	*Arum Ternatum Thunb.*
MOL006947	heptadecanoic acid 2,3-dihydroxy-propyl ester	344.6	25.9	0.26	*Arum Ternatum Thunb.*
MOL006948	inosine	268.26	11.17	0.18	*Arum Ternatum Thunb.*
MOL006949	6-Deoxy-gulose	164.18	44.03	0.03	*Arum Ternatum Thunb.*
MOL006950	(2R)-2-acetamidoglutaric acid	189.19	15.5	0.04	*Arum Ternatum Thunb.*
MOL006951	pedatisectine a	242.27	64.09	0.16	*Arum Ternatum Thunb.*
MOL006952	pedatisectine f	200.22	53.81	0.06	*Arum Ternatum Thunb.*
MOL006953	Thy	126.13	74.2	0.02	*Arum Ternatum Thunb.*
MOL006954	BVE	100.18	42.32	0.01	*Arum Ternatum Thunb.*

MOL000875	Cedrol	222.41	16.23	0.12	*Arum Ternatum Thunb.*
MOL006956	cyclo-(leu-tyr)	276.37	111.16	0.15	*Arum Ternatum Thunb.*
MOL006957	(3S,6S)-3-(benzyl)-6-(4-hydroxybenzyl)piperazine-2,5-quinone	310.38	46.89	0.27	*Arum Ternatum Thunb.*
MOL006958	cyclo-(val-tyr)	262.34	122.79	0.14	*Arum Ternatum Thunb.*
MOL003578	Cycloartenol	426.8	38.69	0.78	*Arum Ternatum Thunb.*
MOL006960	(5R)-5-hydroxy-1-(4-hydroxy-3-methoxyphenyl)tetradecan-3-one	350.55	19.14	0.28	*Arum Ternatum Thunb.*
MOL006961	GR	283.28	20.9	0.21	*Arum Ternatum Thunb.*
MOL006962	2Z-hexadecenoic acid	254.46	34.02	0.1	*Arum Ternatum Thunb.*
MOL006963	Ethyl linolelaidate	308.56	7.33	0.19	*Arum Ternatum Thunb.*
MOL006964	valeraldoxime	101.17	82.58	0.01	*Arum Ternatum Thunb.*
MOL006965	soya-cerebroside ii	714.16	3.86	0.35	*Arum Ternatum Thunb.*
MOL006966	soya-cerebroside ii_qt	552	21.22	0.49	*Arum Ternatum Thunb.*
MOL006967	beta-D-Ribofuranoside, xanthine-9	284.26	44.72	0.21	*Arum Ternatum Thunb.*
MOL000697	MRY	122.14	59.62	0.01	*Arum Ternatum Thunb.*
MOL000708	WLN: VHR	106.13	32.63	0.01	*Arum Ternatum Thunb.*
MOL000071	Istidina	155.18	53.18	0.03	*Arum Ternatum Thunb.*
MOL000748	HMF	126.12	45.07	0.02	*Arum Ternatum Thunb.*
MOL000774	(-)-Citronellal	154.28	35.71	0.02	*Arum Ternatum Thunb.*
MOL000858	Glycerol palmitate	330.57	26.66	0.22	*Arum Ternatum Thunb.*
MOL000860	stearic acid	284.54	17.83	0.14	*Arum Ternatum Thunb.*
MOL000089	catechol	110.12	29.86	0.02	*Arum Ternatum Thunb.*
MOL000908	beta-elemene	204.39	25.63	0.06	*Arum Ternatum Thunb.*
MOL000924	Mnk	170.33	17.66	0.03	*Arum Ternatum Thunb.*
MOL000971	Ethylpalmitate	284.54	18.99	0.14	*Arum Ternatum Thunb.*
MOL001055	5-isopropyl-2-methylbicyclo[3.1.0]hex-2-ene	136.26	47.19	0.04	*Citrus Reticulata*
MOL001101	alpha-Ocimene	136.26	21.43	0.02	*Citrus Reticulata*
MOL001110	cis-beta-Ocimene	136.26	25.38	0.02	*Citrus Reticulata*
MOL000118	(L)-alpha-Terpineol	154.28	48.8	0.03	*Citrus Reticulata*
MOL000121	Decanal	156.3	29.81	0.02	*Citrus Reticulata*
MOL000125	(-)-alpha-Pinene	136.26	46.25	0.05	*Citrus Reticulata*
MOL000127	Neral	152.26	19.48	0.02	*Citrus Reticulata*
MOL000168	()-2-Carene	136.26	46.69	0.04	*Citrus Reticulata*
MOL001797	(2S)-7-[(2S,3R,4S,5S,6R)-4,5-dihydroxy-6-methylol-3-[(2S,3R,4R,5R,6S)-3,4,5-trihydroxy-6-methyl-tetrahydropyran-2-yl]oxy-tetrahydropyran-2-yl]oxy-5-hydroxy-2-(3-hydroxy-5-methoxy-phenyl)chroman-4-one	610.62	11.17	0.7	*Citrus Reticulata*
MOL000198	(R)-linalool	154.28	39.8	0.02	*Citrus Reticulata*
MOL000201	p-Ocimene	136.26	15.06	0.02	*Citrus Reticulata*
MOL002029	()-Cuparene	202.37	38.26	0.07	*Citrus Reticulata*
MOL002050	Isovanillic acid	168.16	39.42	0.04	*Citrus Reticulata*
MOL002092	Antioxidant No. 33	206.36	26.74	0.06	*Citrus Reticulata*
MOL002095	DEP	222.26	52.19	0.07	*Citrus Reticulata*
MOL002138	p-Cymen-8-ol	150.24	32.26	0.03	*Citrus Reticulata*
MOL002336	farnesane	212.47	3.13	0.04	*Citrus Reticulata*

MOL002456	[(3R)-3,7-dimethyloct-6-enyl] butanoate	226.4	21.03	0.06	*Citrus Reticulata*
MOL000259	o-Thymol	150.24	43.28	0.03	*Citrus Reticulata*
MOL000268	(1S,5S)-1-isopropyl-4-methylenebicyclo[3.1.0]hexane	136.26	46.21	0.04	*Citrus Reticulata*
MOL002868	1-Undecyne	152.31	33.99	0.02	*Citrus Reticulata*
MOL002944	(E)-Linalol pyranoxide	170.28	44.25	0.04	*Citrus Reticulata*
MOL000305	lauric acid	200.36	23.59	0.04	*Citrus Reticulata*
MOL003450	dodec-2-enal	182.34	31.95	0.03	*Citrus Reticulata*
MOL003508	Antak	158.32	16.85	0.02	*Citrus Reticulata*
MOL003538	()-Ledene	204.39	51.84	0.1	*Citrus Reticulata*
MOL000359	sitosterol	414.79	36.91	0.75	*Citrus Reticulata*
MOL003949	Dimethyl anthranilate	165.21	65.87	0.04	*Citrus Reticulata*
MOL004328	naringenin	272.27	59.29	0.21	*Citrus Reticulata*
MOL007930	hesperidin	610.62	13.33	0.67	*Citrus Reticulata*
MOL005100	5,7-dihydroxy-2-(3-hydroxy-4-methoxyphenyl)chroman-4-one	302.3	47.74	0.27	*Citrus Reticulata*
MOL005577	undecanal	170.33	22.9	0.03	*Citrus Reticulata*
MOL000057	DIBP	278.38	49.63	0.13	*Citrus Reticulata*
MOL005811	Hepta-3	432.46	23.91	0.58	*Citrus Reticulata*
MOL005812	naringin	580.59	6.92	0.78	*Citrus Reticulata*
MOL005813	Germacrene A	204.39	19.21	0.06	*Citrus Reticulata*
MOL005814	tangeretin	372.4	21.38	0.43	*Citrus Reticulata*
MOL005815	Citromitin	404.45	86.9	0.51	*Citrus Reticulata*
MOL005816	alpha-Sinensal	218.37	57.79	0.06	*Citrus Reticulata*
MOL005817	2-(2-butynyl)-cyclohexanone	150.24	47.78	0.03	*Citrus Reticulata*
MOL005818	2,5,5-trimethylhepta-1,6-diene	138.28	44.34	0.02	*Citrus Reticulata*
MOL005819	2,6,11-trimethyldodecane	212.47	14.19	0.05	*Citrus Reticulata*
MOL005820	22410-74-8	154.28	39.91	0.02	*Citrus Reticulata*
MOL005821	(2S)-2-ethoxypentane	116.23	39.6	0.01	*Citrus Reticulata*
MOL005822	3-decyn-2-ol	154.28	39.33	0.02	*Citrus Reticulata*
MOL005823	Isoprenol	86.15	65.72	0	*Citrus Reticulata*
MOL005824	4-ACETYLBENZOIC ACID	164.17	28.66	0.04	*Citrus Reticulata*
MOL005825	6-Hepten-1-ol	114.21	21.34	0.01	*Citrus Reticulata*
MOL005826	cis-2,6-Dimethyl-2,6-octadiene	138.28	21.1	0.01	*Citrus Reticulata*
MOL005827	Eufin	118.15	0.26	0.01	*Citrus Reticulata*
MOL005828	nobiletin	402.43	61.67	0.52	*Citrus Reticulata*
MOL005829	Undecyl acetate	214.39	19.92	0.05	*Citrus Reticulata*
MOL000597	Neryl acetate	196.32	57.47	0.04	*Citrus Reticulata*
MOL000608	()-Terpinen-4-ol	154.28	81.41	0.03	*Citrus Reticulata*
MOL000615	delta-amorphene	204.39	17.95	0.08	*Citrus Reticulata*
MOL000635	vanillin	152.16	52	0.03	*Citrus Reticulata*
MOL000696	β-terpineol	154.28	47.89	0.03	*Citrus Reticulata*
MOL000710	OYA	128.24	19.07	0.01	*Citrus Reticulata*
MOL000748	HMF	126.12	45.07	0.02	*Citrus Reticulata*
MOL000771	p-coumaric acid	164.17	43.29	0.04	*Citrus Reticulata*
MOL000860	stearic acid	284.54	17.83	0.14	*Citrus Reticulata*
MOL000869	Henicosane	296.65	8.41	0.15	*Citrus Reticulata*
MOL000922	(R)-p-Menth-1-en-4-ol	154.28	32.16	0.03	*Citrus Reticulata*
MOL000114	vanillic acid	168.16	35.47	0.04	*Trichosanthes Kirilowii Maxim*

MOL001393	myristic acid	228.42	21.18	0.07	*Trichosanthes Kirilowii Maxim*
MOL001398	Methyllinolenate	292.51	46.15	0.17	*Trichosanthes Kirilowii Maxim*
MOL001494	Mandenol	308.56	42	0.19	*Trichosanthes Kirilowii Maxim*
MOL001640	NON	172.3	26.74	0.03	*Trichosanthes Kirilowii Maxim*
MOL001641	METHYL LINOLEATE	294.53	41.93	0.17	*Trichosanthes Kirilowii Maxim*
MOL001739	zoomaric acid	254.46	35.78	0.1	*Trichosanthes Kirilowii Maxim*
MOL002038	9E,12Z-octadecadienoic acid	280.5	41.9	0.14	*Trichosanthes Kirilowii Maxim*
MOL002083	tricin	330.31	27.86	0.34	*Trichosanthes Kirilowii Maxim*
MOL002254	Barolub	807.49	16.29	0.22	*Trichosanthes Kirilowii Maxim*
MOL002682	aldehydo-D-galactose	180.18	47.81	0.03	*Trichosanthes Kirilowii Maxim*
MOL002881	Diosmetin	300.28	31.14	0.27	*Trichosanthes Kirilowii Maxim*
MOL000003	MTL	182.2	17.73	0.03	*Trichosanthes Kirilowii Maxim*
MOL000305	lauric acid	200.36	23.59	0.04	*Trichosanthes Kirilowii Maxim*
MOL003050	nonanoic acid	158.27	40.51	0.02	*Trichosanthes Kirilowii Maxim*
MOL003969	L-Serin	105.11	98.47	0.01	*Trichosanthes Kirilowii Maxim*
MOL003971	Threonin	119.14	73.52	0.01	*Trichosanthes Kirilowii Maxim*
MOL000041	PHA	165.21	41.62	0.04	*Trichosanthes Kirilowii Maxim*
MOL000432	linolenic acid	278.48	45.01	0.15	*Trichosanthes Kirilowii Maxim*
MOL004355	Spinasterol	412.77	42.98	0.76	*Trichosanthes Kirilowii Maxim*
MOL000458	campesterol	400.76	5.57	0.72	*Trichosanthes Kirilowii Maxim*
MOL004690	RNS	164.18	40.73	0.03	*Trichosanthes Kirilowii Maxim*
MOL004691	aldehydo-D-ribose	150.15	40.76	0.02	*Trichosanthes Kirilowii Maxim*
MOL004739	DAL	89.11	85.17	0.01	*Trichosanthes Kirilowii Maxim*
MOL000050	GLY	75.08	48.74	0	*Trichosanthes Kirilowii Maxim*
MOL000515	Melissic acid	452.9	13.22	0.49	*Trichosanthes Kirilowii Maxim*
MOL000052	Gulutamine	147.15	6.66	0.02	*Trichosanthes Kirilowii Maxim*
MOL000054	L-	174.24	47.64	0.03	*Trichosanthes Kirilowii Maxim*
MOL005448	Leucinum	131.2	72.92	0.01	*Trichosanthes Kirilowii Maxim*
MOL005449	h-Met-h	149.24	70.87	0.01	*Trichosanthes Kirilowii Maxim*
MOL000055	L-Lysin	146.22	29.33	0.02	*Trichosanthes Kirilowii Maxim*
MOL005530	Hydroxygenkwanin	300.28	36.47	0.27	*Trichosanthes Kirilowii Maxim*
MOL000056	DTY	181.21	57.55	0.05	*Trichosanthes Kirilowii Maxim*
MOL000602	FUM	116.08	17.74	0.01	*Trichosanthes Kirilowii Maxim*
MOL000061	Prolinum	115.15	77.57	0.01	*Trichosanthes Kirilowii Maxim*
MOL000635	vanillin	152.16	52	0.03	*Trichosanthes Kirilowii Maxim*
MOL000065	ASI	133.12	79.74	0.02	*Trichosanthes Kirilowii Maxim*
MOL000659	Montanic acid	424.84	13.69	0.46	*Trichosanthes Kirilowii Maxim*
MOL000662	Ceric acid	396.78	14.24	0.4	*Trichosanthes Kirilowii Maxim*
MOL000663	lignoceric acid	368.72	14.9	0.33	*Trichosanthes Kirilowii Maxim*
MOL000067	L-Valin	117.17	53.33	0.01	*Trichosanthes Kirilowii Maxim*
MOL000675	oleic acid	282.52	33.13	0.14	*Trichosanthes Kirilowii Maxim*
MOL006756	Schottenol	414.79	37.42	0.75	*Trichosanthes Kirilowii Maxim*
MOL000068	L-Ile	131.2	59.05	0.02	*Trichosanthes Kirilowii Maxim*
MOL000069	palmitic acid	256.48	19.3	0.1	*Trichosanthes Kirilowii Maxim*
MOL006946	adenosine	255.27	19.85	0.16	*Trichosanthes Kirilowii Maxim*
MOL000071	Istidina	155.18	53.18	0.03	*Trichosanthes Kirilowii Maxim*
MOL007161	1-tri-chosanoyl-2- linolenic-3-palmitoyl-glucerin	851.49	36.04	0.15	*Trichosanthes Kirilowii Maxim*
MOL007162	1-tri-chosanoyl-2-linoleoyl-3-palmitoyl-glucerin	853.51	33.97	0.16	*Trichosanthes Kirilowii Maxim*
MOL007163	1-trichosanoyl-2,3-dilinoleoyl-glycerin	877.53	36.98	0.14	*Trichosanthes Kirilowii Maxim*

MOL007164	1-trichosanoyl-2,3-linolenic-glycerin	873.49	38.92	0.13	*Trichosanthes Kirilowii Maxim*
MOL007165	10α-cucurbita-5,24-diene-3β-ol	426.8	44.02	0.74	*Trichosanthes Kirilowii Maxim*
MOL007166	1,3-ditrichosanoyl-2-linoleoyl-glycerin	875.51	37.81	0.14	*Trichosanthes Kirilowii Maxim*
MOL007167	2,2'bioxazolidine-3,3'-diethanol	232.32	3.51	0.09	*Trichosanthes Kirilowii Maxim*
MOL007168	Odhpca	139.12	43.07	0.03	*Trichosanthes Kirilowii Maxim*
MOL007169	4-hydroxy-2-methoxybenzoic acid	168.16	50.76	0.04	*Trichosanthes Kirilowii Maxim*
MOL007170	cirsiumaldehyde	234.22	41.38	0.11	*Trichosanthes Kirilowii Maxim*
MOL007171	5-dehydrokarounidiol	438.76	30.23	0.77	*Trichosanthes Kirilowii Maxim*
MOL007172	7-oxo-dihydrokaro-unidiol	456.78	36.85	0.75	*Trichosanthes Kirilowii Maxim*
MOL007173	beta-D-arabinopyranose	150.15	52.64	0.03	*Trichosanthes Kirilowii Maxim*
MOL007174	5-hydroxy-2-(3-hydroxy-4-methoxyphenyl)-7-[(2S,3R,4S,5S,6R)-3,4,5-trihydroxy-6-(hydroxymethyl)oxan-2-yl]oxychromen-4-one	462.44	28.08	0.82	*Trichosanthes Kirilowii Maxim*
MOL007175	karounidiol 3-o-benzoate	544.89	43.99	0.5	*Trichosanthes Kirilowii Maxim*
MOL007176	ARA	150.15	46.48	0.03	*Trichosanthes Kirilowii Maxim*
MOL007177	FCY	121.18	22.1	0.01	*Trichosanthes Kirilowii Maxim*
MOL007178	1,4-D-Galactonolactone	178.16	59.77	0.04	*Trichosanthes Kirilowii Maxim*
MOL007179	Linolenic acid ethyl ester	306.54	46.1	0.2	*Trichosanthes Kirilowii Maxim*
MOL007180	vitamin-e	490.69	32.29	0.7	*Trichosanthes Kirilowii Maxim*
MOL007181	Punicic acid	278.48	44.9	0.15	*Trichosanthes Kirilowii Maxim*
MOL007182	5-hydroxy-2-[4-hydroxy-3-[(2S,3R,4S,5S,6R)-3,4,5-trihydroxy-6-(hydroxymethyl)oxan-2-yl]oxyphenyl]-7-methoxychromen-4-one	462.44	8.01	0.83	*Trichosanthes Kirilowii Maxim*
MOL007183	dibutyl (2R)-2-hydroxybutanedioate	246.34	42.99	0.07	*Trichosanthes Kirilowii Maxim*
MOL007184	[(2R,5S)-5-(hydroxymethyl)oxolan-2-yl]methanol	132.18	72.52	0.02	*Trichosanthes Kirilowii Maxim*
MOL007185	isokarounidiol	440.78	29.16	0.77	*Trichosanthes Kirilowii Maxim*
MOL007186	Karounidiol	440.78	26.26	0.77	*Trichosanthes Kirilowii Maxim*
MOL007187	stigmast-7,22-dien-3β-o-d-glucoside	574.93	21.2	0.62	*Trichosanthes Kirilowii Maxim*
MOL000731	XYS	150.15	58.74	0.03	*Trichosanthes Kirilowii Maxim*
MOL000734	GLO	180.18	24.44	0.03	*Trichosanthes Kirilowii Maxim*
MOL000748	HMF	126.12	45.07	0.02	*Trichosanthes Kirilowii Maxim*
MOL000860	stearic acid	284.54	17.83	0.14	*Trichosanthes Kirilowii Maxim*
MOL000879	methyl palmitate	270.51	18.09	0.12	*Trichosanthes Kirilowii Maxim*
MOL000971	Ethylpalmitate	284.54	18.99	0.14	*Trichosanthes Kirilowii Maxim*
MOL001002	ellagic acid	302.2	43.06	0.43	*Radix Paeoniae Rubra*
MOL000106	PYG	126.12	22.98	0.02	*Radix Paeoniae Rubra*
MOL000114	vanillic acid	168.16	35.47	0.04	*Radix Paeoniae Rubra*
MOL000131	EIC	280.5	41.9	0.14	*Radix Paeoniae Rubra*
MOL001801	salicylic acid	138.13	32.13	0.03	*Radix Paeoniae Rubra*
MOL001906	Methylgallate	184.16	30.91	0.05	*Radix Paeoniae Rubra*
MOL001907	Progallin A	198.19	25.61	0.06	*Radix Paeoniae Rubra*
MOL001918	paeoniflorgenone	318.35	87.59	0.37	*Radix Paeoniae Rubra*
MOL001921	Lactiflorin	462.49	49.12	0.8	*Radix Paeoniae Rubra*
MOL001924	paeoniflorin	480.51	53.87	0.79	*Radix Paeoniae Rubra*
MOL001925	paeoniflorin_qt	318.35	68.18	0.4	*Radix Paeoniae Rubra*
MOL001932	galloylpaeoniflorin	632.62	3.03	0.42	*Radix Paeoniae Rubra*
MOL000219	BOX	121.12	31.55	0.02	*Radix Paeoniae Rubra*
MOL002714	baicalein	270.25	33.52	0.21	*Radix Paeoniae Rubra*
MOL002776	Baicalin	446.39	40.12	0.75	*Radix Paeoniae Rubra*

MOL000357	Sitogluside	576.95	20.63	0.62	*Radix Paeoniae Rubra*
MOL000358	beta-sitosterol	414.79	36.91	0.75	*Radix Paeoniae Rubra*
MOL000359	sitosterol	414.79	36.91	0.75	*Radix Paeoniae Rubra*
MOL000361	Amyrin	426.8	17.6	0.76	*Radix Paeoniae Rubra*
MOL004355	Spinasterol	412.77	42.98	0.76	*Radix Paeoniae Rubra*
MOL000449	Stigmasterol	412.77	43.83	0.76	*Radix Paeoniae Rubra*
MOL000463	16844-71-6	428.82	27.34	0.76	*Radix Paeoniae Rubra*
MOL000492	(+)-catechin	290.29	54.83	0.24	*Radix Paeoniae Rubra*
MOL000508	Friedelin	426.8	29.16	0.76	*Radix Paeoniae Rubra*
MOL005090	oxypaeoniflorin_qt	334.35	19.4	0.44	*Radix Paeoniae Rubra*
MOL000513	3,4,5-trihydroxybenzoic acid	170.13	31.69	0.04	*Radix Paeoniae Rubra*
MOL000578	arbutin	272.28	6.82	0.15	*Radix Paeoniae Rubra*
MOL000579	hydroquinone	110.12	29.26	0.02	*Radix Paeoniae Rubra*
MOL006179	2-(6-carboxy-2,3,4-trihydroxyphenyl)-3,4,5-trihydroxybenzoic acid	338.24	15.21	0.27	*Radix Paeoniae Rubra*
MOL006765	peonidin	301.29	26.92	0.27	*Radix Paeoniae Rubra*
MOL006798	pedunculagin	784.57	37.81	0.07	*Radix Paeoniae Rubra*
MOL000069	palmitic acid	256.48	19.3	0.1	*Radix Paeoniae Rubra*
MOL006990	(1S,2S,4R)-trans-2-hydroxy-1,8-cineole-B-D-glucopyranoside	332.44	30.25	0.27	*Radix Paeoniae Rubra*
MOL006991	60761-00-4	170.28	45.44	0.06	*Radix Paeoniae Rubra*
MOL006992	(2R,3R)-4-methoxyl-distylin	318.3	59.98	0.3	*Radix Paeoniae Rubra*
MOL006993	1-o-beta-d-glucopyranosyl-8-o-benzoylpaeonisuffrone	464.51	12.62	0.77	*Radix Paeoniae Rubra*
MOL006994	1-o-beta-d-glucopyranosyl-8-o-benzoylpaeonisuffrone_qt	302.35	36.01	0.3	*Radix Paeoniae Rubra*
MOL006995	1-o-beta-d-glucopyranosylpaeonisuffrone	494.54	26.62	0.78	*Radix Paeoniae Rubra*
MOL006996	1-o-beta-d-glucopyranosylpaeonisuffrone_qt	332.38	65.08	0.35	*Radix Paeoniae Rubra*
MOL006997	2-[(2R,5R,6R)-6,10-dimethylspiro[4.5]dec-9-en-2-yl]propan-2-ol	222.41	18.44	0.09	*Radix Paeoniae Rubra*
MOL009092	Pentagalloylglucose	940.72	3.01	0.21	*Radix Paeoniae Rubra*
MOL006999	stigmast-7-en-3-ol	414.79	37.42	0.75	*Radix Paeoniae Rubra*
MOL007000	2-methoxy-5-(e)-propenyl-phenol-beta-vicianoside	458.51	4.38	0.65	*Radix Paeoniae Rubra*
MOL007001	2-methoxy-5-[(Z)-prop-1-enyl]phenol	164.22	58.73	0.04	*Radix Paeoniae Rubra*
MOL007002	paeonioflorin	480.51	10.22	0.79	*Radix Paeoniae Rubra*
MOL007003	benzoyl paeoniflorin	584.62	31.14	0.54	*Radix Paeoniae Rubra*
MOL007004	Albiflorin	480.51	30.25	0.77	*Radix Paeoniae Rubra*
MOL007005	Albiflorin_qt	318.35	48.7	0.33	*Radix Paeoniae Rubra*
MOL007006	oxypaeoniflorin	496.51	12.98	0.78	*Radix Paeoniae Rubra*
MOL007007	4-ethyl-paeoniflorin	494.54	24.81	0.82	*Radix Paeoniae Rubra*
MOL007008	4-ethyl-paeoniflorin_qt	332.38	56.87	0.44	*Radix Paeoniae Rubra*
MOL007009	4-o-galloylalbiflorin	632.62	3.09	0.61	*Radix Paeoniae Rubra*
MOL007010	4-o-galloylalbiflorin_qt	470.46	2.14	0.8	*Radix Paeoniae Rubra*
MOL007011	4-o-methyl-paeoniflorin	494.54	25.71	0.78	*Radix Paeoniae Rubra*
MOL007012	4-o-methyl-paeoniflorin_qt	332.38	56.7	0.43	*Radix Paeoniae Rubra*
MOL003867	Paeonolide	460.48	6.3	0.64	*Radix Paeoniae Rubra*
MOL007014	8-debenzoylpaeonidanin	390.43	31.74	0.45	*Radix Paeoniae Rubra*
MOL007015	8-debenzoylpaeonidanin_qt	228.27	129.3	0.15	*Radix Paeoniae Rubra*
MOL007016	Paeoniflorigenone	318.35	65.33	0.37	*Radix Paeoniae Rubra*

MOL007017	9-ethyl-neo-paeoniaflorin A	496.56	23.64	0.68	*Radix Paeoniae Rubra*
MOL007018	9-ethyl-neo-paeoniaflorin A_qt	334.4	64.42	0.3	*Radix Paeoniae Rubra*
MOL007019	Eugeniin	938.7	10.06	0.13	*Radix Paeoniae Rubra*
MOL007020	Lactiflorin_qt	318.35	20.44	0.33	*Radix Paeoniae Rubra*
MOL007021	trans-.beta.-Terpinyl benzoate	258.39	46.89	0.12	*Radix Paeoniae Rubra*
MOL007022	evofolinB	318.35	64.74	0.22	*Radix Paeoniae Rubra*
MOL007023	galloylpaeoniflorine	632.62	3.03	0.42	*Radix Paeoniae Rubra*
MOL007024	galloylpaeoniflorine_qt 2	318.35	28.18	0.4	*Radix Paeoniae Rubra*
MOL007025	isobenzoylpaeoniflorin	584.62	31.14	0.54	*Radix Paeoniae Rubra*
MOL007026	paeonin,a	372.41	20.35	0.37	*Radix Paeoniae Rubra*
MOL007027	paeonin,a_qt	210.25	73.79	0.09	*Radix Paeoniae Rubra*
MOL007028	paeonin,b	358.38	17.29	0.34	*Radix Paeoniae Rubra*
MOL007029	paeonin,b_qt	196.22	105.45	0.08	*Radix Paeoniae Rubra*
MOL007030	paeonin,c	372.41	10.26	0.36	*Radix Paeoniae Rubra*
MOL007031	paeonin,c_qt	210.25	72.7	0.09	*Radix Paeoniae Rubra*
MOL000874	paeonol	166.19	28.79	0.04	*Radix Paeoniae Rubra*
MOL000023	Hemo-sol	136.26	39.84	0.02	*Radix Paeoniae Rubra*
MOL000172	Furol	96.09	34.35	0.01	*Radix Paeoniae Rubra*
MOL000202	Moslene	136.26	33.02	0.02	*Radix Paeoniae Rubra*
MOL000244	()-Borneol	154.28	81.8	0.05	*Radix Paeoniae Rubra*
MOL000254	eugenol	164.22	56.24	0.04	*Radix Paeoniae Rubra*
MOL000305	lauric acid	200.36	23.59	0.04	*Radix Paeoniae Rubra*
MOL000607	Dehydro-p-cymene	132.22	11.63	0.02	*Radix Paeoniae Rubra*
MOL000610	TRD	184.41	17.89	0.03	*Radix Paeoniae Rubra*
MOL000666	hexanal	100.18	55.71	0.01	*Radix Paeoniae Rubra*
MOL000675	oleic acid	282.52	33.13	0.14	*Radix Paeoniae Rubra*
MOL000708	WLN: VHR	106.13	32.63	0.01	*Radix Paeoniae Rubra*
MOL000842	sucrose	342.34	7.17	0.23	*Radix Paeoniae Rubra*
MOL000865	hexadecane	226.5	12.32	0.06	*Radix Paeoniae Rubra*
MOL000867	Heptadekan	240.53	8.64	0.07	*Radix Paeoniae Rubra*
MOL000868	LFA	282.62	8.46	0.13	*Radix Paeoniae Rubra*
MOL000869	Henicosane	296.65	8.41	0.15	*Radix Paeoniae Rubra*
MOL000879	methyl palmitate	270.51	18.09	0.12	*Radix Paeoniae Rubra*
MOL000880	Tricosane	324.71	8.33	0.21	*Radix Paeoniae Rubra*
MOL000971	Ethylpalmitate	284.54	18.99	0.14	*Radix Paeoniae Rubra*
MOL001392	Methyl myristate	242.45	19.68	0.08	*Radix Paeoniae Rubra*
MOL001393	myristic acid	228.42	21.18	0.07	*Radix Paeoniae Rubra*
MOL001394	Oktadekan	254.56	9.81	0.09	*Radix Paeoniae Rubra*
MOL001396	PENTADECYLIC ACID	242.45	20.18	0.08	*Radix Paeoniae Rubra*
MOL001399	TWT	310.68	8.37	0.18	*Radix Paeoniae Rubra*
MOL001401	HEXACOSANE	366.8	8.21	0.31	*Radix Paeoniae Rubra*
MOL001619	UPL	268.59	8.52	0.11	*Radix Paeoniae Rubra*
MOL001641	METHYL LINOLEATE	294.53	41.93	0.17	*Radix Paeoniae Rubra*
MOL001746	ELD	281.54	31.2	0.14	*Radix Paeoniae Rubra*
MOL001747	Tetracosane	338.74	8.28	0.24	*Radix Paeoniae Rubra*
MOL001752	EUG	150.19	38.39	0.03	*Radix Paeoniae Rubra*
MOL001935	(3aR,6S,7aR)-6-hydroxy-6-methyl-3-methylene-3a,4,7,7a-tetrahydrobenzofuran-2,5-dione	196.22	97.79	0.08	*Radix Paeoniae Rubra*

MOL002092	Antioxidant No. 33	206.36	26.74	0.06	*Radix Paeoniae Rubra*
MOL002850	butylated hydroxytoluene	220.39	40.02	0.07	*Radix Paeoniae Rubra*
MOL002883	Ethyl oleate (NF)	310.58	32.4	0.19	*Radix Paeoniae Rubra*
MOL003032	BZM	212.26	18.64	0.09	*Radix Paeoniae Rubra*
MOL004929	Pentadecanol	228.47	13.73	0.06	*Radix Paeoniae Rubra*
MOL005043	campest-5-en-3beta-ol	400.76	37.58	0.71	*Radix Paeoniae Rubra*
MOL005089	Oxypaeoniflorin	496.51	8.38	0.78	*Radix Paeoniae Rubra*
MOL005368	Methyl tricosanoate	368.72	14.61	0.33	*Radix Paeoniae Rubra*
MOL005470	Durol	134.24	17.74	0.03	*Radix Paeoniae Rubra*
MOL005610	WLN: QR BV1	136.16	24.15	0.03	*Radix Paeoniae Rubra*
MOL009569	4,7-DIMETHYLBENZOFURAN	146.2	55.66	0.04	*Radix Paeoniae Rubra*
MOL010564	NK	122.13	32.1	0.02	*Radix Paeoniae Rubra*
MOL012220	Saligenol	124.15	29.43	0.02	*Radix Paeoniae Rubra*
MOL000103	PHB	138.13	30.15	0.03	*Chuanxiong Rhizoma*
MOL001055	5-isopropyl-2-methylbicyclo[3.1.0]hex-2-ene	136.26	47.19	0.04	*Chuanxiong Rhizoma*
MOL000114	vanillic acid	168.16	35.47	0.04	*Chuanxiong Rhizoma*
MOL000116	Nonanal	142.27	40.28	0.02	*Chuanxiong Rhizoma*
MOL000117	Cymol	134.24	27.2	0.02	*Chuanxiong Rhizoma*
MOL002085	alpha-Cubebene	204.39	16.73	0.11	*Chuanxiong Rhizoma*
MOL000118	(L)-alpha-Terpineol	154.28	48.8	0.03	*Chuanxiong Rhizoma*
MOL001201	(1R,5R,7S)-4,7-dimethyl-7-(4-methylpent-3-enyl)bicyclo[3.1.1]hept-3-ene	204.39	16.23	0.09	*Chuanxiong Rhizoma*
MOL001210	(4S)-4-isopropylcyclohexene-1-carbaldehyde	152.26	40.36	0.03	*Chuanxiong Rhizoma*
MOL000122	1,8-cineole	154.28	39.73	0.05	*Chuanxiong Rhizoma*
MOL001223	(S)-2,2,3-Trimethylcyclopent-3-ene-1-acetaldehyde	152.26	45.18	0.03	*Chuanxiong Rhizoma*
MOL000125	(-)-alpha-Pinene	136.26	46.25	0.05	*Chuanxiong Rhizoma*
MOL000126	(-)-nopinene	136.26	44.84	0.05	*Chuanxiong Rhizoma*
MOL001306	o-Acetyl-p-cresol	150.19	24.96	0.03	*Chuanxiong Rhizoma*
MOL000131	EIC	280.5	41.9	0.14	*Chuanxiong Rhizoma*
MOL001335	WLN: Q1R	108.15	58.68	0.01	*Chuanxiong Rhizoma*
MOL001390	49070_FLUKA	222.41	85.51	0.12	*Chuanxiong Rhizoma*
MOL001494	Mandenol	308.56	42	0.19	*Chuanxiong Rhizoma*
MOL001579	germacrene	208.43	15.06	0.06	*Chuanxiong Rhizoma*
MOL001641	METHYL LINOLEATE	294.53	41.93	0.17	*Chuanxiong Rhizoma*
MOL000165	2-[(2S,5S,6S)-6,10-dimethylspiro[4.5]dec-9-en-2-yl]propan-2-ol	222.41	37.62	0.09	*Chuanxiong Rhizoma*
MOL000172	Furol	96.09	34.35	0.01	*Chuanxiong Rhizoma*
MOL001729	Crysophanol	254.25	18.64	0.21	*Chuanxiong Rhizoma*
MOL001744	uracil	112.1	42.53	0.02	*Chuanxiong Rhizoma*
MOL001752	EUG	150.19	38.39	0.03	*Chuanxiong Rhizoma*
MOL001787	ADO	267.28	15.98	0.18	*Chuanxiong Rhizoma*
MOL001788	adenine	135.15	62.81	0.03	*Chuanxiong Rhizoma*
MOL001819	METHYL PENTADECANOATE	256.48	18.82	0.1	*Chuanxiong Rhizoma*
MOL000019	D-Camphene	136.26	34.98	0.04	*Chuanxiong Rhizoma*
MOL000196	L-Bornyl acetate	196.32	65.52	0.08	*Chuanxiong Rhizoma*
MOL000197	Myrcene	136.26	24.96	0.02	*Chuanxiong Rhizoma*
MOL000198	(R)-linalool	154.28	39.8	0.02	*Chuanxiong Rhizoma*
MOL000200	(S)-(+)-alpha-Phellandrene	136.26	27.9	0.02	*Chuanxiong Rhizoma*
MOL000201	p-Ocimene	136.26	15.06	0.02	*Chuanxiong Rhizoma*

MOL000202	Moslene	136.26	33.02	0.02	*Chuanxiong Rhizoma*
MOL002028	(+)-beta-Phellandrene	136.26	40.3	0.02	*Chuanxiong Rhizoma*
MOL000204	-cis-.beta.-Elemene diastereomer	204.39	28.62	0.06	*Chuanxiong Rhizoma*
MOL002042	thymol	150.24	41.47	0.03	*Chuanxiong Rhizoma*
MOL000207	Methyleugenol	178.25	73.36	0.04	*Chuanxiong Rhizoma*
MOL000208	()-Aromadendrene	204.39	55.74	0.1	*Chuanxiong Rhizoma*
MOL002096	(+)-ALPHA-FUNEBRENE	204.39	52.87	0.1	*Chuanxiong Rhizoma*
MOL002097	gem-Dimethylcyclopentane	98.21	41.22	0.01	*Chuanxiong Rhizoma*
MOL002098	3-Butylidene-7-hydroxyphthalide	204.24	62.68	0.08	*Chuanxiong Rhizoma*
MOL002099	Senkyunolide-K	208.28	61.75	0.08	*Chuanxiong Rhizoma*
MOL002100	(3Z,6S,7R)-3-butylidene-6-butyryl-7-hydroxy-4,5,6,7-tetrahydroisobenzofuran-1-one	278.38	3.41	0.16	*Chuanxiong Rhizoma*
MOL002101	Senkyunolide-N	226.3	37.27	0.1	*Chuanxiong Rhizoma*
MOL002102	Levistolid A	380.52	2.15	0.82	*Chuanxiong Rhizoma*
MOL002103	Senkyunolide-P	382.54	9.38	0.81	*Chuanxiong Rhizoma*
MOL002104	Senkyunolide-Q	278.38	26.84	0.16	*Chuanxiong Rhizoma*
MOL002105	Senkyunolide-R	240.28	13.14	0.11	*Chuanxiong Rhizoma*
MOL002106	1,1-Diethoxybutane	146.26	29.28	0.01	*Chuanxiong Rhizoma*
MOL002107	Valerophenone	162.25	42.58	0.03	*Chuanxiong Rhizoma*
MOL002108	(1S,5S)-7,7-dimethyl-2-methylenebicyclo[3.1.1]hept-3-ene	134.24	37.71	0.05	*Chuanxiong Rhizoma*
MOL002109	Z-6,8',7,3'-diligustilide	380.52	11.98	0.7	*Chuanxiong Rhizoma*
MOL002110	Allocymene	136.26	14.89	0.02	*Chuanxiong Rhizoma*
MOL002111	BdPh	188.24	42.44	0.07	*Chuanxiong Rhizoma*
MOL002112	alpha-Selinene	218.42	31.81	0.1	*Chuanxiong Rhizoma*
MOL002113	1,2,3,4,4a,7-Hexahydro-l,6-dimethyl-4-(1-methylethyl)-naphthalene	162.3	19.03	0.05	*Chuanxiong Rhizoma*
MOL002114	augustic-acid	472.78	21.08	0.74	*Chuanxiong Rhizoma*
MOL002115	m-Ethyltoluene	120.21	50.77	0.02	*Chuanxiong Rhizoma*
MOL002116	(1R,4S,5R)-4-isopropenyl-1,8-dimethylspiro[4.5]dec-8-ene	204.39	40.01	0.07	*Chuanxiong Rhizoma*
MOL002117	β-sesquiphellandrene	204.39	23.68	0.06	*Chuanxiong Rhizoma*
MOL002118	(4aS,7S,8aR)-7-isopropenyl-4a-methyl-1-methylenedecalin	204.39	23.65	0.08	*Chuanxiong Rhizoma*
MOL002119	Artemisia triene	136.26	42.1	0.02	*Chuanxiong Rhizoma*
MOL002120	betea-CUBEBENE	204.39	32.16	0.11	*Chuanxiong Rhizoma*
MOL002121	(1S,4E,8E,10R)-4,8,11,11-tetramethylbicyclo[8.1.0]undeca-4,8-diene	204.39	21.69	0.08	*Chuanxiong Rhizoma*
MOL002122	(Z)-Ligustilide	188.24	53.72	0.07	*Chuanxiong Rhizoma*
MOL002123	Chuanxiongol	218.27	22.19	0.1	*Chuanxiong Rhizoma*
MOL002124	beta-asarone	208.28	35.61	0.06	*Chuanxiong Rhizoma*
MOL002125	cis-Piperitol	154.28	43.26	0.03	*Chuanxiong Rhizoma*
MOL002126	(1S,4R,5R)-1-isopropyl-4-methyl-4-bicyclo[3.1.0]hexanol	154.28	27.22	0.05	*Chuanxiong Rhizoma*
MOL002127	Cnidilide	194.3	77.55	0.07	*Chuanxiong Rhizoma*
MOL002128	1,3,8-p-Menthatriene	134.24	35.86	0.02	*Chuanxiong Rhizoma*
MOL002129	CYCLODODECENE	166.34	47.89	0.03	*Chuanxiong Rhizoma*
MOL002130	cyclohexane,1,1,2,3-tetramethyl-	140.3	48.08	0.03	*Chuanxiong Rhizoma*
MOL002131	2,9-Dimethyldecane	170.38	9.93	0.02	*Chuanxiong Rhizoma*

MOL002132	(2R,4aR)-2-isopropenyl-4a,8-dimethyl-2,3,4,5,6,7-hexahydro-1H-naphthalene	204.39	22.13	0.08	*Chuanxiong Rhizoma*
MOL002133	1,5,5-trimethyl-6-methylenecyclohexene	136.26	46.08	0.03	*Chuanxiong Rhizoma*
MOL002134	Isobutyrophenone	148.22	80.37	0.03	*Chuanxiong Rhizoma*
MOL002135	Myricanone	356.45	40.6	0.51	*Chuanxiong Rhizoma*
MOL002136	neocnidilide	194.3	83.83	0.07	*Chuanxiong Rhizoma*
MOL002137	OCT	114.26	29.72	0.01	*Chuanxiong Rhizoma*
MOL002138	p-Cymen-8-ol	150.24	32.26	0.03	*Chuanxiong Rhizoma*
MOL002139	APH	150.2	24.76	0.03	*Chuanxiong Rhizoma*
MOL002140	Perlolyrine	264.3	65.95	0.27	*Chuanxiong Rhizoma*
MOL002141	PLO	316.53	14.07	0.43	*Chuanxiong Rhizoma*
MOL002142	sedanoic-acid	210.3	44.69	0.06	*Chuanxiong Rhizoma*
MOL002143	senkyunolide-C	204.24	46.8	0.08	*Chuanxiong Rhizoma*
MOL002144	senkyunolide-D	222.26	79.13	0.1	*Chuanxiong Rhizoma*
MOL002145	senkyunolide-E	204.24	34.4	0.08	*Chuanxiong Rhizoma*
MOL002146	senkyunolide-F	206.26	40.35	0.08	*Chuanxiong Rhizoma*
MOL002147	senkyunolide-J	226.3	21.14	0.1	*Chuanxiong Rhizoma*
MOL002148	senkyunolide-L	242.72	29.64	0.09	*Chuanxiong Rhizoma*
MOL002149	senkyunolide-S	240.33	20.61	0.11	*Chuanxiong Rhizoma*
MOL002150	1-Acetyl-beta-carboline	210.25	67.12	0.13	*Chuanxiong Rhizoma*
MOL002151	senkyunone	326.52	47.66	0.24	*Chuanxiong Rhizoma*
MOL002152	sinapic acid	224.23	64.15	0.08	*Chuanxiong Rhizoma*
MOL002153	1H-Cycloprop(e)azulen-7-ol, decahydro-1,1,7-trimethyl-4-methylene-, (1aR-(1aalpha,4aalpha,7beta,7abeta,7balpha))-	220.39	82.33	0.12	*Chuanxiong Rhizoma*
MOL002154	trans-2-Nonen-1-ol	142.27	19.96	0.02	*Chuanxiong Rhizoma*
MOL002177	trans-Piperitol	154.28	47.83	0.03	*Chuanxiong Rhizoma*
MOL002156	TML	59.13	59.98	0	*Chuanxiong Rhizoma*
MOL002157	wallichilide	412.57	42.31	0.71	*Chuanxiong Rhizoma*
MOL002158	xiongterpene	574.87	23.77	0.42	*Chuanxiong Rhizoma*
MOL002159	1-Octanol,2,7-dimethyl-	158.32	24.43	0.02	*Chuanxiong Rhizoma*
MOL002160	1-terpineol	154.28	49.83	0.03	*Chuanxiong Rhizoma*
MOL002161	1-beta-ethylacrylate-7-aldehyde-beta-carboline	294.33	28.53	0.31	*Chuanxiong Rhizoma*
MOL002162	2-Propionylfuran	124.15	63.12	0.02	*Chuanxiong Rhizoma*
MOL002163	WLN: 2VR	134.19	60.17	0.02	*Chuanxiong Rhizoma*
MOL002164	2,2,3-Trimethylcyclopent-3-ene-1-carboxaldehyde	138.23	42.64	0.03	*Chuanxiong Rhizoma*
MOL002165	methyl 2-pentanoylbenzoate	220.29	69.28	0.07	*Chuanxiong Rhizoma*
MOL002166	ISOHEPTANE	100.23	59.94	0.01	*Chuanxiong Rhizoma*
MOL002167	WLN: T5OJ BVO1	126.12	49.41	0.02	*Chuanxiong Rhizoma*
MOL002168	2-Methyl-1-phenylpropene	132.22	20.17	0.02	*Chuanxiong Rhizoma*
MOL002169	2-methyl-5-(1-methylene)-1,3-cyclohexadiene	106.18	39.91	0.03	*Chuanxiong Rhizoma*
MOL002170	(4S,6S)-cis-Carveol	152.26	32.5	0.03	*Chuanxiong Rhizoma*
MOL002171	2-Methylbenzoxazol	133.16	65.25	0.03	*Chuanxiong Rhizoma*
MOL002172	(5S,6R)-5,6-dimethyltetrahydropyran-2-one	128.19	48.07	0.02	*Chuanxiong Rhizoma*
MOL002173	3(S)-3-Butyl-4,5-dihydrophthalide	194.3	25.76	0.07	*Chuanxiong Rhizoma*
MOL002174	3,4-epoxy-2,2,7,7-tetramethyl-octane	184.36	66.87	0.05	*Chuanxiong Rhizoma*
MOL002175	3-cyclohexen-1-ol	98.16	70.57	0.01	*Chuanxiong Rhizoma*
MOL002176	Methyl 3-furoate	126.12	77.82	0.02	*Chuanxiong Rhizoma*

MOL002178	4,7-Dihydroxy-3-butylphthalide	222.26	106.09	0.1	*Chuanxiong Rhizoma*
MOL002179	4-iodoindoline	245.07	26.88	0.03	*Chuanxiong Rhizoma*
MOL002180	4-Octanone	128.24	19.37	0.01	*Chuanxiong Rhizoma*
MOL002181	4-hydroxy-3-butylphthalide	206.26	70.31	0.08	*Chuanxiong Rhizoma*
MOL002182	(-)-spathulenol	220.39	25.82	0.12	*Chuanxiong Rhizoma*
MOL002183	5-Propyl-2-thiouracil	170.26	77.6	0.03	*Chuanxiong Rhizoma*
MOL002184	(6R)-6-butylcyclohepta-1,4-diene	150.29	31.69	0.02	*Chuanxiong Rhizoma*
MOL002185	7-oxabicyclo-2.2.1-heptane,1-methyl-4-[1-methylethyl]-	154.28	60.92	0.04	*Chuanxiong Rhizoma*
MOL002186	Aromadendrene oxide 2	220.39	65.1	0.14	*Chuanxiong Rhizoma*
MOL002187	Amylbenzene	148.27	34.34	0.03	*Chuanxiong Rhizoma*
MOL002188	Dimethyl D-malate	162.16	11.47	0.03	*Chuanxiong Rhizoma*
MOL002189	dl-3n-butylphthalide	190.26	47.9	0.07	*Chuanxiong Rhizoma*
MOL002190	Cedrene	204.39	51.14	0.11	*Chuanxiong Rhizoma*
MOL002191	carotol	222.41	149.03	0.09	*Chuanxiong Rhizoma*
MOL002192	Coniferylfcrulate	356.4	4.54	0.39	*Chuanxiong Rhizoma*
MOL002193	Cerulignol	166.24	62.43	0.04	*Chuanxiong Rhizoma*
MOL002194	Decahydro-1,6-bis(methylene)-4-(1-methylethyl)-naphthalene	204.39	28.34	0.08	*Chuanxiong Rhizoma*
MOL003127	Germacrene D	204.39	19.22	0.06	*Chuanxiong Rhizoma*
MOL002196	Hexaphenone	176.28	19.88	0.04	*Chuanxiong Rhizoma*
MOL002197	(E,E)-1,3,5-Undecatriene	150.29	34.61	0.02	*Chuanxiong Rhizoma*
MOL002198	Heptan	100.23	41.8	0	*Chuanxiong Rhizoma*
MOL002199	L-valyl-L-valinc-achydride	214.35	40.18	0.05	*Chuanxiong Rhizoma*
MOL002200	Levistolide-A	380.52	9.96	0.82	*Chuanxiong Rhizoma*
MOL002201	cis-ligustilide	190.26	51.3	0.07	*Chuanxiong Rhizoma*
MOL002202	tetramethylpyrazine	136.22	20.01	0.03	*Chuanxiong Rhizoma*
MOL002203	Exceparl M-OL	296.55	31.9	0.16	*Chuanxiong Rhizoma*
MOL002204	506-43-4	266.52	37.76	0.12	*Chuanxiong Rhizoma*
MOL002205	Octatriacontane	535.16	7.91	0.37	*Chuanxiong Rhizoma*
MOL002206	(2-amylphenyl)methanol	178.3	55.59	0.04	*Chuanxiong Rhizoma*
MOL002207	1(3H)-Isobenzofuranone, 3-butyl-3a,4,5,6-tetrahydro-, cis-(-)-	194.3	65.03	0.07	*Chuanxiong Rhizoma*
MOL002208	Senkyunolide A	192.28	26.56	0.07	*Chuanxiong Rhizoma*
MOL002209	Senkyunolide G	208.28	39.52	0.08	*Chuanxiong Rhizoma*
MOL002210	(3Z,6S,7S)-3-butylidene-6,7-dihydroxy-4,5,6,7-tetrahydroisobenzofuran-1-one	224.28	26.78	0.1	*Chuanxiong Rhizoma*
MOL000223	caffeic acid	180.17	25.76	0.05	*Chuanxiong Rhizoma*
MOL000234	L-Limonen	136.26	38.09	0.02	*Chuanxiong Rhizoma*
MOL000024	alpha-humulene	204.39	22.98	0.06	*Chuanxiong Rhizoma*
MOL000264	Tereben	136.26	29.62	0.02	*Chuanxiong Rhizoma*
MOL000268	(1S,5S)-1-isopropyl-4-methylenebicyclo[3.1.0]hexane	136.26	46.21	0.04	*Chuanxiong Rhizoma*
MOL000027	alpha-Curcumene	202.37	4.68	0.06	*Chuanxiong Rhizoma*
MOL000270	CHEBI:7	136.26	45.2	0.04	*Chuanxiong Rhizoma*
MOL000302	Undekansaeure	186.33	30.14	0.03	*Chuanxiong Rhizoma*

MOL000034	2-[(1R,3S,4S)-3-isopropenyl-4-methyl-4-vinylcyclohexyl]propan-2-ol	222.41	19.03	0.07	*Chuanxiong Rhizoma*
MOL000035	beta-Selinene	204.39	24.39	0.08	*Chuanxiong Rhizoma*
MOL000357	Sitogluside	576.95	20.63	0.62	*Chuanxiong Rhizoma*
MOL000359	sitosterol	414.79	36.91	0.75	*Chuanxiong Rhizoma*
MOL000433	FA	441.45	68.96	0.71	*Chuanxiong Rhizoma*
MOL000608	()-Terpinen-4-ol	154.28	81.41	0.03	*Chuanxiong Rhizoma*
MOL000635	vanillin	152.16	52	0.03	*Chuanxiong Rhizoma*
MOL000666	hexanal	100.18	55.71	0.01	*Chuanxiong Rhizoma*
MOL000668	PENTYLFURAN	138.23	54.59	0.02	*Chuanxiong Rhizoma*
MOL000675	oleic acid	282.52	33.13	0.14	*Chuanxiong Rhizoma*
MOL000069	palmitic acid	256.48	19.3	0.1	*Chuanxiong Rhizoma*
MOL000698	(R)-(-)-alpha-Phellandrene	136.26	27.51	0.02	*Chuanxiong Rhizoma*
MOL000705	WLN: VH6	114.21	19.59	0.01	*Chuanxiong Rhizoma*
MOL000710	OYA	128.24	19.07	0.01	*Chuanxiong Rhizoma*
MOL000712	o-Cymol	134.24	51.89	0.02	*Chuanxiong Rhizoma*
MOL000842	sucrose	342.34	7.17	0.23	*Chuanxiong Rhizoma*
MOL000860	stearic acid	284.54	17.83	0.14	*Chuanxiong Rhizoma*
MOL000864	MYS	212.47	13.98	0.05	*Chuanxiong Rhizoma*
MOL000879	methyl palmitate	270.51	18.09	0.12	*Chuanxiong Rhizoma*
MOL000886	tetradecane	198.44	15.94	0.04	*Chuanxiong Rhizoma*
MOL000908	beta-elemene	204.39	25.63	0.06	*Chuanxiong Rhizoma*
MOL000911	Terpilene	136.26	33.95	0.02	*Chuanxiong Rhizoma*
MOL000922	(R)-p-Menth-1-en-4-ol	154.28	32.16	0.03	*Chuanxiong Rhizoma*
MOL000932	alpha-Farnesene	204.39	21.7	0.05	*Chuanxiong Rhizoma*
MOL000933	.gamma.-Bisabolene	204.39	20.78	0.06	*Chuanxiong Rhizoma*
MOL000937	58870_FLUKA	204.39	49.01	0.1	*Chuanxiong Rhizoma*
MOL000971	Ethylpalmitate	284.54	18.99	0.14	*Chuanxiong Rhizoma*
MOL000131	EIC	280.5	41.9	0.14	*Persicae Semen*
MOL001315	campesterol-3-O-β-D-glucopyranoside	562.92	20.49	0.67	*Persicae Semen*
MOL001316	campesterol-3-O-β-D-glucopyranoside_qt	400.76	7.86	0.72	*Persicae Semen*
MOL001317	β-sitosterol 3-O-β-D-(6-O-oleyl)glucopyranoside	841.45	26.94	0.16	*Persicae Semen*
MOL001318	β-sitosterol-3-(6-palmitoyl)glucopyranoside	815.41	26.07	0.18	*Persicae Semen*
MOL001319	3-feruloylquinic acid	368.37	19.31	0.36	*Persicae Semen*
MOL001320	Amygdalin	457.48	4.42	0.61	*Persicae Semen*
MOL001321	d-mandelonitrile	133.16	48.26	0.02	*Persicae Semen*
MOL001955	Heriguard	354.34	11.93	0.33	*Persicae Semen*
MOL001323	Sitosterol alpha1	426.8	43.28	0.78	*Persicae Semen*
MOL001324	campesterol-3-O-β-D-(6-O-oleyl)glucopyranoside	827.42	27.03	0.17	*Persicae Semen*
MOL001325	campesterol-3-O-β-D-(6-O-palmityl)glucopyranoside	801.38	25.65	0.19	*Persicae Semen*
MOL001327	2,3-didehydro GA69	330.41	14.28	0.5	*Persicae Semen*
MOL001328	2,3-didehydro GA70	330.41	63.29	0.5	*Persicae Semen*
MOL001329	2,3-didehydro GA77	346.41	88.08	0.53	*Persicae Semen*
MOL001330	2,3-didehydro GA9	314.41	17.03	0.45	*Persicae Semen*
MOL001331	Amygdalinic acid	476.48	4.15	0.63	*Persicae Semen*
MOL001332	RMN	152.16	43.67	0.03	*Persicae Semen*
MOL001333	7-dehydroavenasterol	412.77	10.03	0.76	*Persicae Semen*
MOL001334	Benzyl Beta -gentiobioside	432.47	3.46	0.56	*Persicae Semen*

MOL001335	WLN: Q1R	108.15	58.68	0.01	*Persicae Semen*
MOL001336	(2S,3R,4S,5S,6R)-2-(benzyloxy)-6-methylol-tetrahydropyran-3,4,5-triol	270.31	17.14	0.14	*Persicae Semen*
MOL001337	Benzyl glucopyranoside	270.31	12.39	0.14	*Persicae Semen*
MOL001338	GA118	348.43	10.41	0.53	*Persicae Semen*
MOL001339	GA119	332.43	76.36	0.49	*Persicae Semen*
MOL001340	GA120	314.41	84.85	0.45	*Persicae Semen*
MOL001341	GA121	330.41	14.13	0.5	*Persicae Semen*
MOL001342	GA121-isolactone	330.41	72.7	0.54	*Persicae Semen*
MOL001343	GA122	330.41	64.79	0.5	*Persicae Semen*
MOL001344	GA122-isolactone	330.41	88.11	0.54	*Persicae Semen*
MOL001345	Methyl-alpha-D-fructofuranoside	194.21	65.63	0.05	*Persicae Semen*
MOL001346	GA126	346.41	11.8	0.53	*Persicae Semen*
MOL001347	GA16	348.43	14.26	0.53	*Persicae Semen*
MOL001348	gibberellin 17	378.46	94.64	0.49	*Persicae Semen*
MOL001349	4a-formyl-7alpha-hydroxy-1-methyl-8-methylidene-4aalpha,4bbeta-gibbane-1alpha,10beta-dicarboxylic acid	362.46	88.6	0.46	*Persicae Semen*
MOL001350	GA30	346.41	61.72	0.54	*Persicae Semen*
MOL001351	Gibberellin A44	346.46	101.61	0.54	*Persicae Semen*
MOL001352	GA54	348.43	64.21	0.53	*Persicae Semen*
MOL001353	GA60	348.43	93.17	0.53	*Persicae Semen*
MOL001354	GA61	332.43	14.82	0.49	*Persicae Semen*
MOL001355	GA63	348.43	65.54	0.54	*Persicae Semen*
MOL001356	MGL	194.21	24.46	0.05	*Persicae Semen*
MOL001357	GA69	332.43	17.67	0.49	*Persicae Semen*
MOL001358	gibberellin 7	330.41	73.8	0.5	*Persicae Semen*
MOL001359	GA70	332.43	14.04	0.49	*Persicae Semen*
MOL001360	GA77	348.43	87.89	0.53	*Persicae Semen*
MOL001361	GA87	362.41	68.85	0.57	*Persicae Semen*
MOL001362	GA95	330.41	20.01	0.49	*Persicae Semen*
MOL001363	GA97	364.48	10.12	0.47	*Persicae Semen*
MOL001364	(2S)-2-phenyl-2-[(2S,3R,4S,5S,6R)-3,4,5-trihydroxy-6-(hydroxymethyl)oxan-2-yl]oxyacetic acid	314.32	8.27	0.2	*Persicae Semen*
MOL001365	prunasin	295.32	12.61	0.18	*Persicae Semen*
MOL001366	MNN	133.16	48.36	0.02	*Persicae Semen*
MOL001367	[(2S,3R,4S,5S,6R)-3,4,5-trihydroxy-6-(hydroxymethyl)oxan-2-yl] (E)-3-(4-hydroxyphenyl)prop-2-enoate	326.33	9.8	0.26	*Persicae Semen*
MOL001368	3-O-p-coumaroylquinic acid	338.34	37.63	0.29	*Persicae Semen*
MOL001369	Grandidentatin	424.49	10.56	0.54	*Persicae Semen*
MOL001370	[2-[(2S,3R,4S,5S,6R)-3,4,5-trihydroxy-6-(hydroxymethyl)oxan-2-yl]oxyphenyl]methyl (E)-3-(3,4-dihydroxyphenyl)prop-2-enoate	448.46	8.22	0.69	*Persicae Semen*
MOL001371	Populoside_qt	286.3	108.89	0.2	*Persicae Semen*

MOL001372	beta-D-Glucopyranoside, 2-((benzoyloxy)methyl)-4-hydroxyphenyl	406.42	13.51	0.53	*Persicae Semen*
MOL001373	Salireposide_qt	244.26	24.3	0.12	*Persicae Semen*
MOL001901	24-Methylenecycloartanol	440.83	10.4	0.79	*Persicae Semen*
MOL000256	Olein	885.61	27.27	0.13	*Persicae Semen*
MOL000295	alexandrin	576.95	20.63	0.63	*Persicae Semen*
MOL000296	hederagenin	414.79	36.91	0.75	*Persicae Semen*
MOL000358	beta-sitosterol	414.79	36.91	0.75	*Persicae Semen*
MOL000397	cis-p-Coumarate	164.17	45.98	0.04	*Persicae Semen*
MOL000493	campesterol	400.76	37.58	0.71	*Persicae Semen*
MOL000012	Arachic acid	312.6	16.66	0.19	*Rehmanniae Radix Praeparata*
MOL000131	EIC	280.5	41.9	0.14	*Rehmanniae Radix Praeparata*
MOL001396	PENTADECYLIC ACID	242.45	20.18	0.08	*Rehmanniae Radix Praeparata*
MOL001436	leonuride	348.39	2.6	0.33	*Rehmanniae Radix Praeparata*
MOL001501	Daturic acid	270.51	18.51	0.12	*Rehmanniae Radix Praeparata*
MOL003333	acteoside	624.65	2.94	0.62	*Rehmanniae Radix Praeparata*
MOL001709	methyl (2E,4E)-hexadeca-2,4-dienoate	266.47	41.57	0.12	*Rehmanniae Radix Praeparata*
MOL001739	zoomaric acid	254.46	35.78	0.1	*Rehmanniae Radix Praeparata*
MOL001768	Sumiki's acid	142.12	52.44	0.03	*Rehmanniae Radix Praeparata*
MOL001772	Dihydro-beta-ionone	194.35	26.25	0.05	*Rehmanniae Radix Praeparata*
MOL002321	Pca	129.13	96.25	0.02	*Rehmanniae Radix Praeparata*
MOL002819	catalpol	362.37	5.07	0.44	*Rehmanniae Radix Praeparata*
MOL002820	catapol_qt	200.21	44.69	0.1	*Rehmanniae Radix Praeparata*
MOL000003	MTL	182.2	17.73	0.03	*Rehmanniae Radix Praeparata*
MOL000305	lauric acid	200.36	23.59	0.04	*Rehmanniae Radix Praeparata*
MOL003202	8-epi-Loganic acid	376.4	4.43	0.4	*Rehmanniae Radix Praeparata*
MOL003331	Forsythiaside	624.65	3.05	0.61	*Rehmanniae Radix Praeparata*
MOL000346	succinic acid	118.1	29.62	0.01	*Rehmanniae Radix Praeparata*
MOL000357	Sitogluside	576.95	20.63	0.62	*Rehmanniae Radix Praeparata*
MOL000359	sitosterol	414.79	36.91	0.75	*Rehmanniae Radix Praeparata*
MOL000360	FER	194.2	39.56	0.06	*Rehmanniae Radix Praeparata*
MOL003688	2-(4-hydroxyphenyl)ethyl hexacosanoate	516.94	13.12	0.53	*Rehmanniae Radix Praeparata*
MOL003689	aeginetic acid	268.39	48.31	0.13	*Rehmanniae Radix Praeparata*
MOL003690	Ajugol	348.39	16.87	0.32	*Rehmanniae Radix Praeparata*
MOL003691	Ajugoside	390.43	12.15	0.45	*Rehmanniae Radix Praeparata*
MOL003692	Ajugoside_qt	212.27	81.68	0.08	*Rehmanniae Radix Praeparata*
MOL003694	Cerebrosid	266.29	13.81	0.11	*Rehmanniae Radix Praeparata*
MOL003695	Cistanoside A	800.84	3.4	0.33	*Rehmanniae Radix Praeparata*
MOL003696	Cistanoside F	488.49	4.74	0.69	*Rehmanniae Radix Praeparata*
MOL003697	methyl 9,10-methylene-hexadecanoate	282.52	22.94	0.15	*Rehmanniae Radix Praeparata*
MOL003698	(2S,3R,4R,5S,6R)-2-[[(1S,4aS,5R,7aR)-4a,5-dihydroxy-7-methylol-5,7a-dihydro-1H-cyclopenta[c]pyran-1-yl]oxy]-6-methylol-tetrahydropyran-3,4,5-triol	362.37	3.1	0.37	*Rehmanniae Radix Praeparata*
MOL003699	melittoside_qt	200.21	40	0.08	*Rehmanniae Radix Praeparata*
MOL003700	Dihydrocatalpol	364.39	3.58	0.44	*Rehmanniae Radix Praeparata*
MOL003701	geniposide	388.41	3.78	0.44	*Rehmanniae Radix Praeparata*
MOL003702	geniposide_qt	226.25	39.71	0.1	*Rehmanniae Radix Praeparata*
MOL003703	glutinoside	398.83	21.33	0.52	*Rehmanniae Radix Praeparata*

MOL003704	Jiofuran	184.21	54.91	0.06	*Rehmanniae Radix Praeparata*
MOL003705	jioglutin A	250.7	90.7	0.13	*Rehmanniae Radix Praeparata*
MOL003706	jioglutin B	250.7	90.71	0.13	*Rehmanniae Radix Praeparata*
MOL003707	jioglutin C	232.26	2.55	0.13	*Rehmanniae Radix Praeparata*
MOL003708	jioglutin D	246.29	39.02	0.14	*Rehmanniae Radix Praeparata*
MOL003709	jioglutin E	232.31	81.9	0.1	*Rehmanniae Radix Praeparata*
MOL003710	jioglutolide	186.23	86.95	0.06	*Rehmanniae Radix Praeparata*
MOL003711	Jioglutoside A	346.37	3.92	0.39	*Rehmanniae Radix Praeparata*
MOL003712	methyl (1S,4aS,7aS)-7-methylene-1-[(2S,3R,4S,5S,6R)-3,4,5-trihydroxy-6-[[(2R,3R,4R,5S,6R)-3,4,5-trihydroxy-6-methyl-tetrahydropyran-2-yl]oxymethyl]tetrahydropyran-2-yl]oxy-4a,5,6,7a-tetrahydro-1H-cyclopenta[d]pyran-4-carboxylate	518.57	28.27	0.84	*Rehmanniae Radix Praeparata*
MOL003713	Jioglutoside B_qt	210.25	89.22	0.08	*Rehmanniae Radix Praeparata*
MOL003714	Jionoside A	800.84	3.62	0.36	*Rehmanniae Radix Praeparata*
MOL003715	Jionoside B	814.87	4.27	0.35	*Rehmanniae Radix Praeparata*
MOL003716	Melittoside	524.53	19.2	0.8	*Rehmanniae Radix Praeparata*
MOL003717	METHYL PALMITOLEATE	268.49	34.61	0.12	*Rehmanniae Radix Praeparata*
MOL003718	6-O-p-coumaroylajugol	524.57	26.13	0.85	*Rehmanniae Radix Praeparata*
MOL003719	methyl-2,6,10-trimethyltridecanoate	269.5	24.86	0.1	*Rehmanniae Radix Praeparata*
MOL003720	Purpureaside C	786.81	3.14	0.38	*Rehmanniae Radix Praeparata*
MOL003721	Rehmaglutin B	236.67	64.62	0.11	*Rehmanniae Radix Praeparata*
MOL003722	(3aS,4R,6aS)-4-hydroxy-6,6a-dimethylol-3a,4-dihydro-3H-cyclopenta[d]furan-2-one	200.21	29.92	0.08	*Rehmanniae Radix Praeparata*
MOL003723	(2S,3R,4S,5S,6R)-2-[(1R,2R)-2-hydroxy-2-[(E,3S)-3-hydroxybut-1-enyl]-1,3,3-trimethylcyclohexoxy]-6-(hydroxymethyl)tetrahydropyran-3,4,5-triol	390.53	8.43	0.33	*Rehmanniae Radix Praeparata*
MOL003724	(2S,3R,4S,5S,6R)-2-[(1R,2R)-2-hydroxy-2-[(E,3R)-3-hydroxybut-1-enyl]-1,3,3-trimethylcyclohexoxy]-6-(hydroxymethyl)tetrahydropyran-3,4,5-triol	390.53	3.24	0.33	*Rehmanniae Radix Praeparata*
MOL003725	Rehmaionoside C	388.51	12.89	0.34	*Rehmanniae Radix Praeparata*
MOL003726	rehmannioside B	524.53	2.05	0.88	*Rehmanniae Radix Praeparata*
MOL003727	rehmannioside C	510.55	10.23	0.86	*Rehmanniae Radix Praeparata*
MOL003728	6-O-p-hydroxybenzoylajugol	468.5	4.53	0.81	*Rehmanniae Radix Praeparata*
MOL003729	(3R)-2,6,6-trimethyl-3-[(2R,3R,4S,5S,6R)-3,4,5-trihydroxy-6-(hydroxymethyl)oxan-2-yl]oxycyclohexene-1-carboxylic acid	346.42	13.59	0.26	*Rehmanniae Radix Praeparata*
MOL003730	Rehmannioside A	524.53	25.95	0.87	*Rehmanniae Radix Praeparata*
MOL003731	Rehmaglutin A	202.23	29.7	0.1	*Rehmanniae Radix Praeparata*
MOL003732	Rehmaglutin D	220.67	57.03	0.1	*Rehmanniae Radix Praeparata*
MOL003733	6-O-vanilloylajugol	484.5	23.44	0.84	*Rehmanniae Radix Praeparata*
MOL003734	echinacoside	786.81	3.14	0.38	*Rehmanniae Radix Praeparata*
MOL003735	aucubin	346.37	4.17	0.33	*Rehmanniae Radix Praeparata*
MOL003736	Acetylcatalpol	402.44	5.53	0.58	*Rehmanniae Radix Praeparata*
MOL000399	Docosanoate	340.66	15.69	0.26	*Rehmanniae Radix Praeparata*
MOL000414	Caffeate	180.17	54.97	0.05	*Rehmanniae Radix Praeparata*
MOL000449	Stigmasterol	412.77	43.83	0.76	*Rehmanniae Radix Praeparata*

MOL000732	Stachyose	666.66	3.25	0.59	*Rehmanniae Radix Praeparata*
MOL000748	HMF	126.12	45.07	0.02	*Rehmanniae Radix Praeparata*
MOL000841	raffinose	504.5	11.79	0.66	*Rehmanniae Radix Praeparata*
MOL000842	sucrose	342.34	7.17	0.23	*Rehmanniae Radix Praeparata*
MOL000105	protocatechuic acid	154.13	25.37	0.04	*Cornus Officinalis Sieb. Et Zucc.*
MOL000118	(L)-alpha-Terpineol	154.28	48.8	0.03	*Cornus Officinalis Sieb. Et Zucc.*
MOL000126	(-)-nopinene	136.26	44.84	0.05	*Cornus Officinalis Sieb. Et Zucc.*
MOL001300	PEL	122.18	44.03	0.02	*Cornus Officinalis Sieb. Et Zucc.*
MOL000131	EIC	280.5	41.9	0.14	*Cornus Officinalis Sieb. Et Zucc.*
MOL001393	myristic acid	228.42	21.18	0.07	*Cornus Officinalis Sieb. Et Zucc.*
MOL001394	Oktadekan	254.56	9.81	0.09	*Cornus Officinalis Sieb. Et Zucc.*
MOL001398	Methyllinolenate	292.51	46.15	0.17	*Cornus Officinalis Sieb. Et Zucc.*
MOL001403	ERUCAMIDE	337.66	27.85	0.26	*Cornus Officinalis Sieb. Et Zucc.*
MOL001468	MLT	134.1	59.62	0.02	*Cornus Officinalis Sieb. Et Zucc.*
MOL001494	Mandenol	308.56	42	0.19	*Cornus Officinalis Sieb. Et Zucc.*
MOL001495	Ethyl linolenate	306.54	46.1	0.2	*Cornus Officinalis Sieb. Et Zucc.*
MOL001562	Nonadecene	266.57	5.23	0.11	*Cornus Officinalis Sieb. Et Zucc.*
MOL001599	α-cubebol	208.38	64.81	0.09	*Cornus Officinalis Sieb. Et Zucc.*
MOL001600	copaene	204.39	29.47	0.12	*Cornus Officinalis Sieb. Et Zucc.*
MOL001604	Linalool	170.28	49.37	0.04	*Cornus Officinalis Sieb. Et Zucc.*
MOL001606	BB_NC-0668	204.39	35.57	0.08	*Cornus Officinalis Sieb. Et Zucc.*
MOL001618	Pellitorin	223.4	23.81	0.06	*Cornus Officinalis Sieb. Et Zucc.*
MOL001619	UPL	268.59	8.52	0.11	*Cornus Officinalis Sieb. Et Zucc.*
MOL001680	Loganin	390.43	5.9	0.44	*Cornus Officinalis Sieb. Et Zucc.*
MOL001681	methyl (1R,4aS,6S,7R,7aS)-1,6-dihydroxy-7-methyl-1,4a,5,6,7,7a-hexahydrocyclopenta[d]pyran-4-carboxylate	228.27	29.99	0.1	*Cornus Officinalis Sieb. Et Zucc.*
MOL001682	(1S,4aS,6R,8S,8aS)-6-hydroxy-8-methyl-1-[(2S,3R,4S,5S,6R)-3,4,5-trihydroxy-6-methylol-tetrahydropyran-2-yl]oxy-1,4a,5,6,8,8a-hexahydropyrano[4,3-d]pyran-4-carboxylic acid methyl ester	406.43	13.86	0.5	*Cornus Officinalis Sieb. Et Zucc.*
MOL001683	morroniside_qt	244.27	1.68	0.12	*Cornus Officinalis Sieb. Et Zucc.*
MOL001696	Morusin	420.49	11.52	0.76	*Cornus Officinalis Sieb. Et Zucc.*
MOL000172	Furol	96.09	34.35	0.01	*Cornus Officinalis Sieb. Et Zucc.*
MOL001739	zoomaric acid	254.46	35.78	0.1	*Cornus Officinalis Sieb. Et Zucc.*
MOL001746	ELD	281.54	31.2	0.14	*Cornus Officinalis Sieb. Et Zucc.*
MOL001771	poriferast-5-en-3beta-ol	414.79	36.91	0.75	*Cornus Officinalis Sieb. Et Zucc.*
MOL001816	Amide HPL	255.5	19.79	0.1	*Cornus Officinalis Sieb. Et Zucc.*
MOL001886	Tar	150.1	66.38	0.02	*Cornus Officinalis Sieb. Et Zucc.*
MOL001889	Methyl linolelaidate	294.53	41.93	0.17	*Cornus Officinalis Sieb. Et Zucc.*
MOL001893	BU3	90.14	34.87	0.01	*Cornus Officinalis Sieb. Et Zucc.*
MOL001906	Methylgallate	184.16	30.91	0.05	*Cornus Officinalis Sieb. Et Zucc.*
MOL000199	Safrol	162.2	45.34	0.05	*Cornus Officinalis Sieb. Et Zucc.*
MOL000206	isoeugenol	164.22	70.1	0.04	*Cornus Officinalis Sieb. Et Zucc.*
MOL000207	Methyleugenol	178.25	73.36	0.04	*Cornus Officinalis Sieb. Et Zucc.*
MOL002167	WLN: T5OJ BVO1	126.12	49.41	0.02	*Cornus Officinalis Sieb. Et Zucc.*
MOL000223	caffeic acid	180.17	25.76	0.05	*Cornus Officinalis Sieb. Et Zucc.*

MOL002250	[(2R,3R,4S,5R,6R)-3,5-dihydroxy-2-(3,4,5-trihydroxybenzoyl)oxy-6-[(3,4,5-trihydroxybenzoyl)oxymethyl]oxan-4-yl] 3,4,5-trihydroxybenzoate	636.51	3.01	0.54	*Cornus Officinalis Sieb. Et Zucc.*
MOL002307	20-Hexadecanoylingenol	586.94	28.2	0.68	*Cornus Officinalis Sieb. Et Zucc.*
MOL002343	tetrandrine	622.82	26.64	0.1	*Cornus Officinalis Sieb. Et Zucc.*
MOL002373	gamma-tocopherol	416.76	15.62	0.52	*Cornus Officinalis Sieb. Et Zucc.*
MOL002534	1,6-dimethyl-4-isopropyl-1,2,3,4,4a,7-hexahydronaphthalene	204.39	17.14	0.08	*Cornus Officinalis Sieb. Et Zucc.*
MOL000261	Myristicin	192.23	17.99	0.07	*Cornus Officinalis Sieb. Et Zucc.*
MOL000263	oleanolic acid	456.78	29.02	0.76	*Cornus Officinalis Sieb. Et Zucc.*
MOL000269	Elemicin	208.28	21.94	0.06	*Cornus Officinalis Sieb. Et Zucc.*
MOL000027	alpha-Curcumene	202.37	4.68	0.06	*Cornus Officinalis Sieb. Et Zucc.*
MOL002703	OCTADECENE	252.54	19.21	0.09	*Cornus Officinalis Sieb. Et Zucc.*
MOL002778	THZ	135.2	70.33	0.03	*Cornus Officinalis Sieb. Et Zucc.*
MOL002850	butylated hydroxytoluene	220.39	40.02	0.07	*Cornus Officinalis Sieb. Et Zucc.*
MOL002879	Diop	390.62	43.59	0.39	*Cornus Officinalis Sieb. Et Zucc.*
MOL002883	Ethyl oleate (NF)	310.58	32.4	0.19	*Cornus Officinalis Sieb. Et Zucc.*
MOL003010	quercetin-3-o-beta-D-glu	478.39	1.81	0.79	*Cornus Officinalis Sieb. Et Zucc.*
MOL000305	lauric acid	200.36	23.59	0.04	*Cornus Officinalis Sieb. Et Zucc.*
MOL003080	2-METHYLPENTADECANE	226.5	4.35	0.06	*Cornus Officinalis Sieb. Et Zucc.*
MOL003137	Leucanthoside	462.44	32.12	0.78	*Cornus Officinalis Sieb. Et Zucc.*
MOL003166	Swertiamarin	374.38	21.9	0.42	*Cornus Officinalis Sieb. Et Zucc.*
MOL003374	Urushiol III	316.53	3.57	0.25	*Cornus Officinalis Sieb. Et Zucc.*
MOL000034	2-[(1R,3S,4S)-3-isopropenyl-4-methyl-4-vinylcyclohexyl]propan-2-ol	222.41	19.03	0.07	*Cornus Officinalis Sieb. Et Zucc.*
MOL003484	PEY	178.24	25.7	0.1	*Cornus Officinalis Sieb. Et Zucc.*
MOL003527	Tyranton	116.18	58.34	0.01	*Cornus Officinalis Sieb. Et Zucc.*
MOL003546	Aristolone	218.37	45.31	0.13	*Cornus Officinalis Sieb. Et Zucc.*
MOL003547	Azaron	208.28	38.39	0.06	*Cornus Officinalis Sieb. Et Zucc.*
MOL000357	Sitogluside	576.95	20.63	0.62	*Cornus Officinalis Sieb. Et Zucc.*
MOL003573	calacorene	200.35	16.2	0.08	*Cornus Officinalis Sieb. Et Zucc.*
MOL000358	beta-sitosterol	414.79	36.91	0.75	*Cornus Officinalis Sieb. Et Zucc.*
MOL000359	sitosterol	414.79	36.91	0.75	*Cornus Officinalis Sieb. Et Zucc.*
MOL003788	Cinnamein	238.3	78.8	0.12	*Cornus Officinalis Sieb. Et Zucc.*
MOL003789	Coumaran	120.16	50.9	0.03	*Cornus Officinalis Sieb. Et Zucc.*
MOL003877	Fuseloel	88.17	65.67	0	*Cornus Officinalis Sieb. Et Zucc.*
MOL003940	Stearamide	283.56	18.44	0.14	*Cornus Officinalis Sieb. Et Zucc.*
MOL003955	D-Cystine	240.34	39.58	0.05	*Cornus Officinalis Sieb. Et Zucc.*
MOL003969	L-Serin	105.11	98.47	0.01	*Cornus Officinalis Sieb. Et Zucc.*
MOL003971	Threonin	119.14	73.52	0.01	*Cornus Officinalis Sieb. Et Zucc.*
MOL004048	keto-L-fructose	180.18	49.67	0.03	*Cornus Officinalis Sieb. Et Zucc.*
MOL000041	PHA	165.21	41.62	0.04	*Cornus Officinalis Sieb. Et Zucc.*
MOL000042	LPG	89.11	87.69	0.01	*Cornus Officinalis Sieb. Et Zucc.*
MOL004284	2-Nonadecanone	282.57	14.38	0.14	*Cornus Officinalis Sieb. Et Zucc.*
MOL000431	coumarin	146.15	29.17	0.04	*Cornus Officinalis Sieb. Et Zucc.*
MOL000449	Stigmasterol	412.77	43.83	0.76	*Cornus Officinalis Sieb. Et Zucc.*
MOL004582	Methyl naphthalene	142.21	39.01	0.04	*Cornus Officinalis Sieb. Et Zucc.*
MOL004590	2-METHYLHEXADECANE	240.53	4.19	0.07	*Cornus Officinalis Sieb. Et Zucc.*

MOL004741	(7aR)-4,4,7a-trimethyl-6,7-dihydro-5H-benzofuran-2-one	180.27	40.48	0.07	*Cornus Officinalis Sieb. Et Zucc.*
MOL000475	anethole	148.22	32.49	0.03	*Cornus Officinalis Sieb. Et Zucc.*
MOL004784	Stenol	270.56	12.66	0.11	*Cornus Officinalis Sieb. Et Zucc.*
MOL000050	GLY	75.08	48.74	0	*Cornus Officinalis Sieb. Et Zucc.*
MOL005022	1,8-DIMETHYLNAPHTHALENE	156.24	42.8	0.05	*Cornus Officinalis Sieb. Et Zucc.*
MOL000511	ursolic acid	456.78	16.77	0.75	*Cornus Officinalis Sieb. Et Zucc.*
MOL001996	Betulonic acid	454.76	16.83	0.78	*Cornus Officinalis Sieb. Et Zucc.*
MOL000513	3,4,5-trihydroxybenzoic acid	170.13	31.69	0.04	*Cornus Officinalis Sieb. Et Zucc.*
MOL000052	Gulutamine	147.15	6.66	0.02	*Cornus Officinalis Sieb. Et Zucc.*
MOL000520	alpha-amyrin	426.8	10.28	0.76	*Cornus Officinalis Sieb. Et Zucc.*
MOL005272	13-Tetradecenyl acetate	254.46	36.76	0.1	*Cornus Officinalis Sieb. Et Zucc.*
MOL005306	Acetal	118.2	26.4	0.01	*Cornus Officinalis Sieb. Et Zucc.*
MOL005360	malkangunin	432.56	57.71	0.63	*Cornus Officinalis Sieb. Et Zucc.*
MOL005386	Vulgarin	264.35	29.21	0.2	*Cornus Officinalis Sieb. Et Zucc.*
MOL005449	h-Met-h	149.24	70.87	0.01	*Cornus Officinalis Sieb. Et Zucc.*
MOL005467	Epicatechin gallate	442.4	17.89	0.75	*Cornus Officinalis Sieb. Et Zucc.*
MOL005468	1,2,3,6-tetra-O-galloyl-β-D-glucose	788.62	3.01	0.33	*Cornus Officinalis Sieb. Et Zucc.*
MOL005469	1,2,3-tri-O-galloyl-β-D-glucose	636.51	3.01	0.58	*Cornus Officinalis Sieb. Et Zucc.*
MOL005470	Durol	134.24	17.74	0.03	*Cornus Officinalis Sieb. Et Zucc.*
MOL005471	[(2R,3R,4S,5S,6R)-4,5-dihydroxy-2-(3,4,5-trihydroxybenzoyl)oxy-6-[(3,4,5-trihydroxybenzoyl)oxymethyl]oxan-3-yl] 3,4,5-trihydroxybenzoate	636.51	3.01	0.54	*Cornus Officinalis Sieb. Et Zucc.*
MOL005472	1,2-Benzenedicarboxylicacid, mono(2-ethyl) hexylester	278.38	55.17	0.13	*Cornus Officinalis Sieb. Et Zucc.*
MOL005473	1,5-DIACETYLNAPHTHALENE	212.26	11.6	0.1	*Cornus Officinalis Sieb. Et Zucc.*
MOL005474	1-Acetyl-4,6,8-trimethylazulene	212.31	27.02	0.09	*Cornus Officinalis Sieb. Et Zucc.*
MOL005475	Chloroicosane	317.06	10.43	0.15	*Cornus Officinalis Sieb. Et Zucc.*
MOL005476	Docosene	308.66	16.74	0.18	*Cornus Officinalis Sieb. Et Zucc.*
MOL005477	Eicosene	280.6	17.83	0.13	*Cornus Officinalis Sieb. Et Zucc.*
MOL005478	11,14-Octadecadienoic acid, methyl ester	294.53	41.93	0.17	*Cornus Officinalis Sieb. Et Zucc.*
MOL005479	pentatriacont-17-ene	491.05	13.19	0.43	*Cornus Officinalis Sieb. Et Zucc.*
MOL005480	Ucar AC	104.17	60.17	0.01	*Cornus Officinalis Sieb. Et Zucc.*
MOL005481	2,6,10,14,18-pentamethylicosa-2,6,10,14,18-pentaene	342.67	33.4	0.24	*Cornus Officinalis Sieb. Et Zucc.*
MOL005482	FOA	112.09	35.66	0.02	*Cornus Officinalis Sieb. Et Zucc.*
MOL005483	2-Methylnaphthalene	142.21	33.69	0.04	*Cornus Officinalis Sieb. Et Zucc.*
MOL005484	GRO	150.19	52.16	0.03	*Cornus Officinalis Sieb. Et Zucc.*
MOL005485	3-dibenzofuransulfonic acid	248.27	74.42	0.16	*Cornus Officinalis Sieb. Et Zucc.*
MOL005486	3,4-Dehydrolycopen-16-al	548.92	46.64	0.49	*Cornus Officinalis Sieb. Et Zucc.*
MOL005487	5-Carboxyresorcinol	154.13	69.34	0.03	*Cornus Officinalis Sieb. Et Zucc.*
MOL005488	3,5-Di-t-butyl-4-hydroxybenzaldehyde	234.37	27.19	0.08	*Cornus Officinalis Sieb. Et Zucc.*
MOL005489	3,6-Digalloylglucose	484.4	31.42	0.66	*Cornus Officinalis Sieb. Et Zucc.*
MOL005490	4-(4-ethylphenyl)benzoic acid	226.29	42.06	0.1	*Cornus Officinalis Sieb. Et Zucc.*
MOL005491	4-Ethyl-o-xylene	134.24	39.27	0.02	*Cornus Officinalis Sieb. Et Zucc.*
MOL005492	4-Methoxy-1,2-benzodioxole	152.16	57.08	0.04	*Cornus Officinalis Sieb. Et Zucc.*
MOL005493	6-Isopropyl-1,4-dimethylnaphthalene	198.33	33.79	0.08	*Cornus Officinalis Sieb. Et Zucc.*
MOL005494	6-Tetradecanesulfonic acid,butyl ester	334.63	21.36	0.13	*Cornus Officinalis Sieb. Et Zucc.*

MOL005495	6-ethyl-2,5-dihydroxy-1,4-naphthoquinone	218.22	20.58	0.1	*Cornus Officinalis Sieb. Et Zucc.*
MOL005496	7,8-Dehydropenstemoside	404.41	3.41	0.47	*Cornus Officinalis Sieb. Et Zucc.*
MOL005497	7,8-Dehydropenstemoside_qt	242.25	108.33	0.12	*Cornus Officinalis Sieb. Et Zucc.*
MOL005498	7-Hydroxycadalene	214.33	29.3	0.09	*Cornus Officinalis Sieb. Et Zucc.*
MOL005499	7-O-methylmorroniside	406.43	3.98	0.5	*Cornus Officinalis Sieb. Et Zucc.*
MOL005500	linolenate	278.48	45.01	0.15	*Cornus Officinalis Sieb. Et Zucc.*
MOL005501	Green Oil	178.24	17.74	0.1	*Cornus Officinalis Sieb. Et Zucc.*
MOL005502	Butoxysuccinic Acid	190.22	37.55	0.04	*Cornus Officinalis Sieb. Et Zucc.*
MOL005503	Cornudentanone	378.56	39.66	0.33	*Cornus Officinalis Sieb. Et Zucc.*
MOL005504	Cornusiin A	1557.19	7.95	0.01	*Cornus Officinalis Sieb. Et Zucc.*
MOL005505	Cornusiin B	1086.77	17.84	0.01	*Cornus Officinalis Sieb. Et Zucc.*
MOL005506	Cornusiin C	784.57	3.01	0.14	*Cornus Officinalis Sieb. Et Zucc.*
MOL005507	Cornusiin G	1725.3	6.95	0.01	*Cornus Officinalis Sieb. Et Zucc.*
MOL005508	Glucosol	472.78	15.86	0.74	*Cornus Officinalis Sieb. Et Zucc.*
MOL005509	11-Cyclohexylheneicosane	378.81	9.94	0.28	*Cornus Officinalis Sieb. Et Zucc.*
MOL005510	D-1-O-Methyl mucoinositol	194.21	25.11	0.05	*Cornus Officinalis Sieb. Et Zucc.*
MOL005511	D-delta-tocopherol	402.73	16.36	0.48	*Cornus Officinalis Sieb. Et Zucc.*
MOL005512	Dehydromorroniaglycone	226.25	58.78	0.13	*Cornus Officinalis Sieb. Et Zucc.*
MOL005513	DBF	168.2	37.28	0.08	*Cornus Officinalis Sieb. Et Zucc.*
MOL005514	Ethyl,alpha-hydroxymyristate	272.48	18.71	0.11	*Cornus Officinalis Sieb. Et Zucc.*
MOL005515	Eugenone	252.29	18.7	0.1	*Cornus Officinalis Sieb. Et Zucc.*
MOL005516	Fluoren	166.23	18.94	0.08	*Cornus Officinalis Sieb. Et Zucc.*
MOL005517	2-METHYLHEPTADECANE	254.56	10.57	0.09	*Cornus Officinalis Sieb. Et Zucc.*
MOL005518	Heptadecane,3-methyl	254.56	10.57	0.09	*Cornus Officinalis Sieb. Et Zucc.*
MOL005519	(7R)-7-methylheptadecane	254.56	10.57	0.08	*Cornus Officinalis Sieb. Et Zucc.*
MOL005520	Heptadecane,8-methyl	254.56	10.57	0.08	*Cornus Officinalis Sieb. Et Zucc.*
MOL005521	phytane	282.62	13.86	0.11	*Cornus Officinalis Sieb. Et Zucc.*
MOL005522	LEN	87.19	62.94	0	*Cornus Officinalis Sieb. Et Zucc.*
MOL005523	Nonadecane,2,3-di methyl	296.65	11.6	0.15	*Cornus Officinalis Sieb. Et Zucc.*
MOL005524	Nonadecane,2,6,10,14,18-pentamethyl,	338.74	13.99	0.2	*Cornus Officinalis Sieb. Et Zucc.*
MOL005525	Octadecane,2,6,10,14-tetramethyl-	310.68	13.58	0.15	*Cornus Officinalis Sieb. Et Zucc.*
MOL005526	Octadecane,3-methyl	268.59	10.42	0.11	*Cornus Officinalis Sieb. Et Zucc.*
MOL005527	Octadecane,6-methyl	268.59	10.42	0.1	*Cornus Officinalis Sieb. Et Zucc.*
MOL005528	2,6,10,14-tetramethylpentadecane	268.59	3.6	0.09	*Cornus Officinalis Sieb. Et Zucc.*
MOL005529	Pentadecane,2,6,10-trimethyl	254.56	13.33	0.08	*Cornus Officinalis Sieb. Et Zucc.*
MOL005530	Hydroxygenkwanin	300.28	36.47	0.27	*Cornus Officinalis Sieb. Et Zucc.*
MOL005531	Telocinobufagin	402.58	69.99	0.79	*Cornus Officinalis Sieb. Et Zucc.*
MOL005532	Tetradecane,2,6,10-trimethyl	240.53	3.93	0.06	*Cornus Officinalis Sieb. Et Zucc.*
MOL008457	Tetrahydroalstonine	352.47	32.42	0.81	*Cornus Officinalis Sieb. Et Zucc.*
MOL005534	trans-Verbenol	152.26	52.01	0.06	*Cornus Officinalis Sieb. Et Zucc.*
MOL005535	Vitamin B1.mol2	266.41	49.33	0.11	*Cornus Officinalis Sieb. Et Zucc.*
MOL005536	Z,Z-10,12-Hexadecadien-1-ol acetate	280.5	44.09	0.14	*Cornus Officinalis Sieb. Et Zucc.*
MOL005537	Z-5-Nonadecene	266.57	18.81	0.11	*Cornus Officinalis Sieb. Et Zucc.*
MOL005538	Linolenyl alcohol	264.5	42.79	0.12	*Cornus Officinalis Sieb. Et Zucc.*
MOL005539	Acenaphthylene	152.2	28.55	0.07	*Cornus Officinalis Sieb. Et Zucc.*
MOL000554	gallic acid-3-O-(6'-O-galloyl)-glucoside	484.4	30.25	0.67	*Cornus Officinalis Sieb. Et Zucc.*

MOL005540	alpha-Corocalene	200.35	13.97	0.08	*Cornus Officinalis Sieb. Et Zucc.*
MOL005541	TBU	74.14	60.45	0	*Cornus Officinalis Sieb. Et Zucc.*
MOL005542	camptothin A	1571.17	6.27	0.02	*Cornus Officinalis Sieb. Et Zucc.*
MOL005543	camptothin B	1086.77	3.01	0.04	*Cornus Officinalis Sieb. Et Zucc.*
MOL005544	Cornin	388.41	12.69	0.44	*Cornus Officinalis Sieb. Et Zucc.*
MOL005545	cornin_qt	226.25	25.1	0.1	*Cornus Officinalis Sieb. Et Zucc.*
MOL005546	cornuside	542.54	2.61	0.71	*Cornus Officinalis Sieb. Et Zucc.*
MOL005547	cornuside_qt	380.38	2.37	0.39	*Cornus Officinalis Sieb. Et Zucc.*
MOL005548	cornusiin E	1857.37	7.37	0	*Cornus Officinalis Sieb. Et Zucc.*
MOL005549	Dehydromevalonic lactone	112.14	53.46	0.02	*Cornus Officinalis Sieb. Et Zucc.*
MOL005550	Vanirom	166.19	28.07	0.04	*Cornus Officinalis Sieb. Et Zucc.*
MOL007019	Eugeniin	938.7	10.06	0.13	*Cornus Officinalis Sieb. Et Zucc.*
MOL005552	gemin D	634.49	68.83	0.56	*Cornus Officinalis Sieb. Et Zucc.*
MOL005553	2-Monopalmitin	330.57	26.74	0.22	*Cornus Officinalis Sieb. Et Zucc.*
MOL005554	i-Butanol	74.14	28.26	0	*Cornus Officinalis Sieb. Et Zucc.*
MOL005555	isoterchebin	954.7	3.01	0.09	*Cornus Officinalis Sieb. Et Zucc.*
MOL005556	1-tert-Butyl-7-methoxynaphthalene	214.33	27.46	0.09	*Cornus Officinalis Sieb. Et Zucc.*
MOL005557	lanosta-8,24-dien-3-ol,3-acetate	468.84	44.3	0.82	*Cornus Officinalis Sieb. Et Zucc.*
MOL005558	DLE	131.2	55.1	0.01	*Cornus Officinalis Sieb. Et Zucc.*
MOL005559	Maslinic acid	472.78	15.54	0.74	*Cornus Officinalis Sieb. Et Zucc.*
MOL005560	olean-13(18)-ene	410.8	6.6	0.76	*Cornus Officinalis Sieb. Et Zucc.*
MOL005561	Anisylacetone	178.25	19.69	0.04	*Cornus Officinalis Sieb. Et Zucc.*
MOL005562	phosphonofluoridic acid,(l-methylethyl)-,cyclohexyl ester	208.24	76	0.04	*Cornus Officinalis Sieb. Et Zucc.*
MOL005563	(-)-Pulegone	152.26	51.23	0.03	*Cornus Officinalis Sieb. Et Zucc.*
MOL005564	tellimagrandin I	786.59	3.01	0.32	*Cornus Officinalis Sieb. Et Zucc.*
MOL005565	Uretan	89.11	3.97	0	*Cornus Officinalis Sieb. Et Zucc.*
MOL005566	urs-12-en-28-al	424.78	8.66	0.77	*Cornus Officinalis Sieb. Et Zucc.*
MOL005567	vitamin a	286.5	19.53	0.16	*Cornus Officinalis Sieb. Et Zucc.*
MOL000057	DIBP	278.38	49.63	0.13	*Cornus Officinalis Sieb. Et Zucc.*
MOL000570	Nonox D	219.3	39.82	0.13	*Cornus Officinalis Sieb. Et Zucc.*
MOL000061	Prolinum	115.15	77.57	0.01	*Cornus Officinalis Sieb. Et Zucc.*
MOL000610	TRD	184.41	17.89	0.03	*Cornus Officinalis Sieb. Et Zucc.*
MOL000635	vanillin	152.16	52	0.03	*Cornus Officinalis Sieb. Et Zucc.*
MOL000644	swertiamarin_qt	212.22	2.58	0.09	*Cornus Officinalis Sieb. Et Zucc.*
MOL000065	ASI	133.12	79.74	0.02	*Cornus Officinalis Sieb. Et Zucc.*
MOL000650	1H,3H-Pyrano(3,4-c)pyran-1-one, 5-ethenyl-6-(beta-D-glucopyranosyloxy)-4,4a,5,6-tetrahydro-, (4aS-(4aalpha,5beta,6alpha))-	358.38	4.96	0.38	*Cornus Officinalis Sieb. Et Zucc.*
MOL000651	Sweroside aglycone	196.22	68.68	0.08	*Cornus Officinalis Sieb. Et Zucc.*
MOL000666	hexanal	100.18	55.71	0.01	*Cornus Officinalis Sieb. Et Zucc.*
MOL000676	DBP	278.38	64.54	0.13	*Cornus Officinalis Sieb. Et Zucc.*
MOL000068	L-Ile	131.2	59.05	0.02	*Cornus Officinalis Sieb. Et Zucc.*
MOL000069	palmitic acid	256.48	19.3	0.1	*Cornus Officinalis Sieb. Et Zucc.*
MOL000704	styrene	104.16	29.55	0.01	*Cornus Officinalis Sieb. Et Zucc.*
MOL000071	Istidina	155.18	53.18	0.03	*Cornus Officinalis Sieb. Et Zucc.*

MOL000749	Linoleic	280.5	41.9	0.14	*Cornus Officinalis Sieb. Et Zucc.*
MOL000775	EEE	88.12	45.02	0	*Cornus Officinalis Sieb. Et Zucc.*
MOL000860	stearic acid	284.54	17.83	0.14	*Cornus Officinalis Sieb. Et Zucc.*
MOL000864	MYS	212.47	13.98	0.05	*Cornus Officinalis Sieb. Et Zucc.*
MOL000865	hexadecane	226.5	12.32	0.06	*Cornus Officinalis Sieb. Et Zucc.*
MOL000867	Heptadekan	240.53	8.64	0.07	*Cornus Officinalis Sieb. Et Zucc.*
MOL000873	CYH	98.16	74.99	0.01	*Cornus Officinalis Sieb. Et Zucc.*
MOL000879	methyl palmitate	270.51	18.09	0.12	*Cornus Officinalis Sieb. Et Zucc.*
MOL000885	Dodekan	170.38	17.74	0.02	*Cornus Officinalis Sieb. Et Zucc.*
MOL000886	tetradecane	198.44	15.94	0.04	*Cornus Officinalis Sieb. Et Zucc.*
MOL000890	(+)-alpha-Curcumene	202.37	26.56	0.06	*Cornus Officinalis Sieb. Et Zucc.*
MOL000971	Ethylpalmitate	284.54	18.99	0.14	*Cornus Officinalis Sieb. Et Zucc.*
MOL000118	(L)-alpha-Terpineol	154.28	48.8	0.03	*Epimrdii Herba*
MOL000120	dec-2-enal	154.28	18.55	0.02	*Epimrdii Herba*
MOL000130	CAM	152.26	67.17	0.05	*Epimrdii Herba*
MOL001510	24-epicampesterol	400.76	37.58	0.71	*Epimrdii Herba*
MOL001579	germacrene	208.43	15.06	0.06	*Epimrdii Herba*
MOL001600	copaene	204.39	29.47	0.12	*Epimrdii Herba*
MOL001640	NON	172.3	26.74	0.03	*Epimrdii Herba*
MOL001645	Linoleyl acetate	308.56	42.1	0.2	*Epimrdii Herba*
MOL001707	24190-29-2	192.33	21.67	0.05	*Epimrdii Herba*
MOL001771	poriferast-5-en-3beta-ol	414.79	36.91	0.75	*Epimrdii Herba*
MOL001789	isoliquiritigenin	256.27	85.32	0.15	*Epimrdii Herba*
MOL001792	DFV	256.27	32.76	0.18	*Epimrdii Herba*
MOL001972	Pulegone	152.26	51.6	0.03	*Epimrdii Herba*
MOL000198	(R)-linalool	154.28	39.8	0.02	*Epimrdii Herba*
MOL000203	Izosafrol	162.2	56.92	0.05	*Epimrdii Herba*
MOL002040	(1S,4R)-fenchone	152.26	72.64	0.05	*Epimrdii Herba*
MOL000205	(6R)-6-isopropyl-3-methyl-1-cyclohex-2-enone	152.26	53.88	0.03	*Epimrdii Herba*
MOL000207	Methyleugenol	178.25	73.36	0.04	*Epimrdii Herba*
MOL002083	tricin	330.31	27.86	0.34	*Epimrdii Herba*
MOL002085	alpha-Cubebene	204.39	16.73	0.11	*Epimrdii Herba*
MOL002307	20-Hexadecanoylingenol	586.94	28.2	0.68	*Epimrdii Herba*
MOL002361	Terragon	148.22	36.59	0.03	*Epimrdii Herba*
MOL000244	()-Borneol	154.28	81.8	0.05	*Epimrdii Herba*
MOL002509	Ginkgetin	566.54	22.19	0.59	*Epimrdii Herba*
MOL002511	Isoginkgetin	566.54	21.56	0.58	*Epimrdii Herba*
MOL000263	oleanolic acid	456.78	29.02	0.76	*Epimrdii Herba*
MOL002689	3,4,5-Trimethoxytoluene	182.24	23.73	0.04	*Epimrdii Herba*
MOL002697	junipene	204.39	44.07	0.11	*Epimrdii Herba*
MOL002891	magnoflorine	342.45	0.48	0.55	*Epimrdii Herba*
MOL002929	salidroside	300.34	7.01	0.2	*Epimrdii Herba*
MOL002930	Tyrosol	138.18	33.81	0.02	*Epimrdii Herba*
MOL003030	Ginnol	424.89	11.33	0.43	*Epimrdii Herba*
MOL003044	Chryseriol	300.28	35.85	0.27	*Epimrdii Herba*
MOL000305	lauric acid	200.36	23.59	0.04	*Epimrdii Herba*
MOL003097	Flavone der.	298.31	27.12	0.27	*Epimrdii Herba*
MOL000325	(2R,3R)-2-(3,4-dimethoxyphenyl)-7-methoxy-3-methyl-5-[(E)-prop-1-enyl]-2,3-dihydrobenzofuran	340.45	24.19	0.35	*Epimrdii Herba*

MOL003518	Vetol	126.12	53.23	0.02	*Epimrdii Herba*
MOL003520	Damascenone	190.31	36.43	0.05	*Epimrdii Herba*
MOL003542	8-Isopentenyl-kaempferol	354.38	38.04	0.39	*Epimrdii Herba*
MOL003547	Azaron	208.28	38.39	0.06	*Epimrdii Herba*
MOL000357	Sitogluside	576.95	20.63	0.62	*Epimrdii Herba*
MOL000359	sitosterol	414.79	36.91	0.75	*Epimrdii Herba*
MOL000399	Docosanoate	340.66	15.69	0.26	*Epimrdii Herba*
MOL000422	kaempferol	286.25	41.88	0.24	*Epimrdii Herba*
MOL004363	(Z)-heptadec-3-ene	238.51	20.24	0.08	*Epimrdii Herba*
MOL004364	3,5-Dimethoxytoluene	152.21	20.87	0.03	*Epimrdii Herba*
MOL004365	Isomenthol	156.3	55.3	0.03	*Epimrdii Herba*
MOL004366	Octyl formate	158.27	53.32	0.02	*Epimrdii Herba*
MOL004367	olivil	376.44	62.23	0.41	*Epimrdii Herba*
MOL004368	Hyperin	464.41	6.94	0.77	*Epimrdii Herba*
MOL004369	quercetin-3-rhamnooside	448.41	2.61	0.74	*Epimrdii Herba*
MOL000437	Hirsutrin	464.41	1.86	0.77	*Epimrdii Herba*
MOL004370	Robinetin	302.25	6.35	0.28	*Epimrdii Herba*
MOL004371	rouhuoside	824.86	3.62	0.31	*Epimrdii Herba*
MOL004372	Sagittatoside A	676.73	8.5	0.57	*Epimrdii Herba*
MOL004373	Anhydroicaritin	368.41	45.41	0.44	*Epimrdii Herba*
MOL004374	sagittatoside B	646.7	5.58	0.64	*Epimrdii Herba*
MOL004375	Trifolin	448.41	3.1	0.74	*Epimrdii Herba*
MOL004376	wanepimedoside A	678.75	6.54	0.58	*Epimrdii Herba*
MOL004377	wanepimedoside_qt	386.43	6.03	0.48	*Epimrdii Herba*
MOL004378	Wushanicariin	530.57	5.49	0.86	*Epimrdii Herba*
MOL004379	wushanicariin_qt	368.41	23.36	0.46	*Epimrdii Herba*
MOL004380	C-Homoerythrinan, 1,6-didehydro-3,15,16-trimethoxy-, (3.beta.)-	329.48	39.14	0.49	*Epimrdii Herba*
MOL004381	Besigomsin	416.51	28.52	0.78	*Epimrdii Herba*
MOL004382	Yinyanghuo A	420.49	56.96	0.77	*Epimrdii Herba*
MOL004383	Yinyanghuo B	422.51	1.07	0.6	*Epimrdii Herba*
MOL004384	Yinyanghuo C	336.36	45.67	0.5	*Epimrdii Herba*
MOL004385	Yinyanghuo D	338.38	13.99	0.38	*Epimrdii Herba*
MOL004386	Yinyanghuo E	352.36	51.63	0.55	*Epimrdii Herba*
MOL004387	Yixinoside A	1107.49	6.13	0.04	*Epimrdii Herba*
MOL004388	6-hydroxy-11,12-dimethoxy-2,2-dimethyl-1,8-dioxo-2,3,4,8-tetrahydro-1H-isochromeno[3,4-h]isoquinolin-2-ium	370.41	60.64	0.66	*Epimrdii Herba*
MOL004389	3-Hexenyl-beta-glucopyranoside	262.34	12.12	0.11	*Epimrdii Herba*
MOL004390	5,7,4'-trihydroxy8,3'-diprenylflavone	406.51	1.02	0.59	*Epimrdii Herba*
MOL004391	8-(3-methylbut-2-enyl)-2-phenyl-chromone	290.38	48.54	0.25	*Epimrdii Herba*
MOL004392	acuminatoside	985.05	3.01	0.15	*Epimrdii Herba*
MOL004393	anhydroicaritin	368.41	28.27	0.59	*Epimrdii Herba*
MOL004394	Anhydroicaritin-3-O-alpha-L-rhamnoside	676.73	41.58	0.61	*Epimrdii Herba*
MOL004395	artonin U	352.41	19.33	0.39	*Epimrdii Herba*
MOL004396	1,2-bis(4-hydroxy-3-methoxyphenyl)propan-1,3-diol	320.37	52.31	0.22	*Epimrdii Herba*
MOL004397	baohuoside Ⅵ	822.89	6.06	0.32	*Epimrdii Herba*

MOL004398	3,5,7-Trihydroxy-4'-methoxyl-8-prenylflavone-3-O-rhamnopyranoside	514.57	3.7	0.84	*Epimrdii Herba*
MOL004399	Baohuoside VI	822.89	4.94	0.31	*Epimrdii Herba*
MOL004400	Bilobanol	234.37	14.22	0.09	*Epimrdii Herba*
MOL004401	bilobetin	552.51	7.27	0.63	*Epimrdii Herba*
MOL004402	brevicornin	400.46	14.09	0.52	*Epimrdii Herba*
MOL004403	caohuoside B	965.01	3.02	0.19	*Epimrdii Herba*
MOL004404	caohuoside D	562.62	24.89	0.83	*Epimrdii Herba*
MOL004405	3-[(2S,3R,4R,5R,6S)-4,5-dihydroxy-6-methyl-3-[(2S,3R,4S,5S,6R)-3,4,5-trihydroxy-6-methyl-tetrahydropyran-2-yl]oxy-tetrahydropyran-2-yl]oxy-5-hydroxy-2-(4-methoxyphenyl)-8-(3-methylbut-2-enyl)-7-[(2S,3R,4S,5S,6R)-3,4,5-trihydroxy-6-methylol-tetrahydropyran	822.89	6.06	0.32	*Epimrdii Herba*
MOL004406	2,15-Hexadecanedione	254.46	8.15	0.1	*Epimrdii Herba*
MOL004407	Epimedin B	792.86	8.65	0.34	*Epimrdii Herba*
MOL004408	Epimedin C_qt	352.41	5.87	0.4	*Epimrdii Herba*
MOL004409	Epimedin C	790.89	16.29	0.34	*Epimrdii Herba*
MOL004410	epimedokoreanone A	142.12	59.85	0.03	*Epimrdii Herba*
MOL004411	(2S,3S)-3,5-dihydroxy-2-(4-hydroxyphenyl)-8-(3-methylbut-2-enyl)-7-[(2S,3R,4S,5S,6R)-3,4,5-trihydroxy-6-methylol-tetrahydropyran-2-yl]oxy-chroman-4-one	518.56	3.58	0.84	*Epimrdii Herba*
MOL004413	epimedoside C	516.54	2.67	0.84	*Epimrdii Herba*
MOL004414	epimedoside D	794.83	5.49	0.34	*Epimrdii Herba*
MOL004415	Epimedoside E	794.83	5.49	0.35	*Epimrdii Herba*
MOL004417	epimedoside	760.81	14.32	0.41	*Epimrdii Herba*
MOL004418	DOB	154.13	88.18	0.04	*Epimrdii Herba*
MOL004419	globulol	222.41	19.94	0.12	*Epimrdii Herba*
MOL004420	Hentriacontanol-6	452.95	11.06	0.54	*Epimrdii Herba*
MOL004421	hexandraside D	822.89	5.44	0.32	*Epimrdii Herba*
MOL004422	Hexandraside E	678.7	13.56	0.6	*Epimrdii Herba*
MOL004423	hexandraside F	838.89	3.67	0.3	*Epimrdii Herba*
MOL004424	Icaride A2	436.5	4.76	0.54	*Epimrdii Herba*
MOL004425	Icariin	676.73	41.58	0.61	*Epimrdii Herba*
MOL004426	Icariresinol	433.47	7.13	0.53	*Epimrdii Herba*
MOL004427	Icariside A7	462.49	31.91	0.86	*Epimrdii Herba*
MOL004428	3,4,6-trimethoxyphenanthrene-2,7-diol	300.33	24.46	0.3	*Epimrdii Herba*
MOL004429	icariside C1	418.59	5.12	0.37	*Epimrdii Herba*
MOL004430	icariside I	530.57	21.88	0.85	*Epimrdii Herba*
MOL004431	icariside II	514.57	3.7	0.84	*Epimrdii Herba*
MOL004432	4H-1-Benzopyran-4-one, 3-((6-deoxy-alpha-L-mennopyranosyl)oxy)-5,7-dihydroxy-2-(4-hydroxyphenyl)-8-(3-methyl-2-butenyl)-	500.54	4.75	0.82	*Epimrdii Herba*
MOL004433	3-[(2S,3R,4S,5S,6R)-4,5-dihydroxy-6-methylol-3-[(2S,3R,4R,5R,6S)-3,4,5-trihydroxy-6-methyl-tetrahydropyran-2-yl]oxy-tetrahydropyran-2-yl]oxy-5,7-dihydroxy-2-(4-hydroxyphenyl)-8-(3-methylbut-2-enyl)chromone	662.7	2.93	0.61	*Epimrdii Herba*

MOL004434	Ikarisoside C	822.89	4.96	0.31	*Epimrdii Herba*
MOL004435	Ikarisoside F	632.67	1.95	0.67	*Epimrdii Herba*
MOL004436	Ikshusterol	430.79	9	0.79	*Epimrdii Herba*
MOL004437	Lespedin	578.57	7.97	0.79	*Epimrdii Herba*
MOL004438	korepimedoside A	748.8	14.93	0.44	*Epimrdii Herba*
MOL004439	korepimedoside B	965.01	3.02	0.19	*Epimrdii Herba*
MOL000472	emodin	270.25	24.4	0.24	*Epimrdii Herba*
MOL000478	Eucarvone	150.24	53.14	0.03	*Epimrdii Herba*
MOL000561	Astragalin	448.41	14.03	0.74	*Epimrdii Herba*
MOL000006	luteolin	286.25	36.16	0.25	*Epimrdii Herba*
MOL000622	Magnograndiolide	266.37	63.71	0.19	*Epimrdii Herba*
MOL000667	1-hexanol	102.2	22.04	0.01	*Epimrdii Herba*
MOL000695	patchouli alcohol	222.41	101.96	0.14	*Epimrdii Herba*
MOL000740	(+)-Cycloolivil	376.44	24.5	0.42	*Epimrdii Herba*
MOL000008	apigenin	270.25	23.06	0.21	*Epimrdii Herba*
MOL000098	quercetin	302.25	46.43	0.28	*Epimrdii Herba*
MOL000011	(2R,3R)-3-(4-hydroxy-3-methoxy-phenyl)-5-methoxy-2-methylol-2,3-dihydropyrano[5,6-h][1,4]benzodioxin-9-one	386.38	68.83	0.66	*Saposhnikoviae Radix*
MOL011387	heptadeca-1,8-dien-4,6-diyn-3,10-diol	260.41	53.86	0.11	*Saposhnikoviae Radix*
MOL000114	vanillic acid	168.16	35.47	0.04	*Saposhnikoviae Radix*
MOL000116	Nonanal	142.27	40.28	0.02	*Saposhnikoviae Radix*
MOL011729	11-hydroxy-sec-o-beta-d-glucosylhamaudol	472.49	16.62	0.77	*Saposhnikoviae Radix*
MOL011730	11-hydroxy-sec-o-beta-d-glucosylhamaudol_qt	292.31	50.24	0.27	*Saposhnikoviae Radix*
MOL011731	3'-O-Acetylhamaudol	318.35	26.21	0.34	*Saposhnikoviae Radix*
MOL011732	anomalin	426.5	59.65	0.66	*Saposhnikoviae Radix*
MOL001945	Majudin	216.2	42.21	0.13	*Saposhnikoviae Radix*
MOL011734	Cimifugin	306.34	13.49	0.29	*Saposhnikoviae Radix*
MOL011735	deltoin	328.39	14.25	0.36	*Saposhnikoviae Radix*
MOL011736	3'-O-angeloylhamaudol	358.42	12.01	0.45	*Saposhnikoviae Radix*
MOL011737	divaricatacid	320.32	87	0.32	*Saposhnikoviae Radix*
MOL011738	divaricataester,a	264.3	64.02	0.13	*Saposhnikoviae Radix*
MOL011739	divaricataester,b	346.41	26.1	0.39	*Saposhnikoviae Radix*
MOL011740	divaricatol	334.35	31.65	0.38	*Saposhnikoviae Radix*
MOL011741	fangfengalpyrimidine	296.36	15.37	0.24	*Saposhnikoviae Radix*
MOL011742	fraxidin	220.24	42.22	0.1	*Saposhnikoviae Radix*
MOL011743	hamaudol	276.31	21.46	0.24	*Saposhnikoviae Radix*
MOL001941	Ammidin	270.3	34.55	0.22	*Saposhnikoviae Radix*
MOL011745	isofraxidin	220.24	39.75	0.1	*Saposhnikoviae Radix*
MOL011746	isopimpinellin	246.23	43.14	0.17	*Saposhnikoviae Radix*
MOL011747	ledebouriellol	374.42	32.05	0.51	*Saposhnikoviae Radix*
MOL011748	nodakenetin	230.28	68.62	0.15	*Saposhnikoviae Radix*
MOL011749	phelloptorin	300.33	43.39	0.28	*Saposhnikoviae Radix*
MOL011750	prim-o-beta-d-glucosylcimifugin	486.52	27	0.79	*Saposhnikoviae Radix*
MOL001950	psoralen	186.17	33.06	0.1	*Saposhnikoviae Radix*
MOL011752	sec-o-beta-d-glucosylhamaudol	456.49	15.08	0.75	*Saposhnikoviae Radix*
MOL011753	5-O-Methylvisamminol	290.34	37.99	0.25	*Saposhnikoviae Radix*
MOL011754	4-hydroxy-9-methoxyfuro[3,2-g]chromen-7-one	232.2	31.78	0.15	*Saposhnikoviae Radix*

MOL011755	5-methoxy-8-hydroxypsoralen	232.2	48.4	0.15	*Saposhnikoviae Radix*
MOL011756	7-octen-4-ol	128.24	33.99	0.01	*Saposhnikoviae Radix*
MOL001953	Uvadex	216.2	35.3	0.13	*Saposhnikoviae Radix*
MOL001787	ADO	267.28	15.98	0.18	*Saposhnikoviae Radix*
MOL001940	Falcarindiol	260.41	39.3	0.11	*Saposhnikoviae Radix*
MOL001944	Marmesin	246.28	50.28	0.18	*Saposhnikoviae Radix*
MOL001946	Ammijin	408.44	15.48	0.69	*Saposhnikoviae Radix*
MOL002029	()-Cuparene	202.37	38.26	0.07	*Saposhnikoviae Radix*
MOL002644	Phellopterin	300.33	40.19	0.28	*Saposhnikoviae Radix*
MOL000003	MTL	182.2	17.73	0.03	*Saposhnikoviae Radix*
MOL000032	beta-Eudesmol	222.41	26.09	0.1	*Saposhnikoviae Radix*
MOL000357	Sitogluside	576.95	20.63	0.62	*Saposhnikoviae Radix*
MOL000359	sitosterol	414.79	36.91	0.75	*Saposhnikoviae Radix*
MOL000373	(2S)-4-methoxy-7-methyl-2-[1-methyl-1-[(2S,3R,4S,5S,6R)-3,4,5-trihydroxy-6-methylol-tetrahydropyran-2-yl]oxy-ethyl]-2,3-dihydrofuro[3,2-g]chromen-5-one	452.5	5.38	0.81	*Saposhnikoviae Radix*
MOL000040	Scopoletol	192.18	27.77	0.08	*Saposhnikoviae Radix*
MOL000663	lignoceric acid	368.72	14.9	0.33	*Saposhnikoviae Radix*
MOL000666	hexanal	100.18	55.71	0.01	*Saposhnikoviae Radix*
MOL000710	OYA	128.24	19.07	0.01	*Saposhnikoviae Radix*
MOL007502	NSC692928	244.41	43.31	0.1	*Saposhnikoviae Radix*
MOL000968	beta-Bisabolene	204.39	29.59	0.06	*Saposhnikoviae Radix*
MOL000018	(+/-)-Isoborneol	154.28	86.98	0.05	*Saposhnikoviae Radix*
MOL000023	Hemo-sol	136.26	39.84	0.02	*Saposhnikoviae Radix*
MOL000035	beta-Selinene	204.39	24.39	0.08	*Saposhnikoviae Radix*
MOL000069	palmitic acid	256.48	19.3	0.1	*Saposhnikoviae Radix*
MOL000115	Undecenal	168.31	39.35	0.03	*Saposhnikoviae Radix*
MOL000117	Cymol	134.24	27.2	0.02	*Saposhnikoviae Radix*
MOL000118	(L)-alpha-Terpineol	154.28	48.8	0.03	*Saposhnikoviae Radix*
MOL000120	dec-2-enal	154.28	18.55	0.02	*Saposhnikoviae Radix*
MOL000122	1,8-cineole	154.28	39.73	0.05	*Saposhnikoviae Radix*
MOL000125	(-)-alpha-Pinene	136.26	46.25	0.05	*Saposhnikoviae Radix*
MOL000128	NERYLACETATE	196.32	25.94	0.04	*Saposhnikoviae Radix*
MOL000129	Acetate C-8	172.3	18.43	0.03	*Saposhnikoviae Radix*
MOL000131	EIC	280.5	41.9	0.14	*Saposhnikoviae Radix*
MOL000162	beta-Chamigrene	204.39	31.99	0.08	*Saposhnikoviae Radix*
MOL000172	Furol	96.09	34.35	0.01	*Saposhnikoviae Radix*
MOL000173	wogonin	284.28	30.68	0.23	*Saposhnikoviae Radix*
MOL000196	L-Bornyl acetate	196.32	65.52	0.08	*Saposhnikoviae Radix*
MOL000197	Myrcene	136.26	24.96	0.02	*Saposhnikoviae Radix*
MOL000198	(R)-linalool	154.28	39.8	0.02	*Saposhnikoviae Radix*
MOL000202	Moslene	136.26	33.02	0.02	*Saposhnikoviae Radix*
MOL000234	L-Limonen	136.26	38.09	0.02	*Saposhnikoviae Radix*
MOL000264	Tereben	136.26	29.62	0.02	*Saposhnikoviae Radix*
MOL000266	beta-Cubebene	204.39	32.81	0.11	*Saposhnikoviae Radix*
MOL000268	(1S,5S)-1-isopropyl-4-methylenebicyclo[3.1.0]hexane	136.26	46.21	0.04	*Saposhnikoviae Radix*
MOL000271	l-carvone	150.24	49.47	0.03	*Saposhnikoviae Radix*

MOL000302	Undekansaeure	186.33	30.14	0.03	*Saposhnikoviae Radix*
MOL000303	caprylic acid	144.24	16.4	0.02	*Saposhnikoviae Radix*
MOL000305	lauric acid	200.36	23.59	0.04	*Saposhnikoviae Radix*
MOL000358	beta-sitosterol	414.79	36.91	0.75	*Saposhnikoviae Radix*
MOL000431	coumarin	146.15	29.17	0.04	*Saposhnikoviae Radix*
MOL000474	(-)-Epoxycaryophyllene	220.39	35.94	0.13	*Saposhnikoviae Radix*
MOL000597	Neryl acetate	196.32	57.47	0.04	*Saposhnikoviae Radix*
MOL000602	FUM	116.08	17.74	0.01	*Saposhnikoviae Radix*
MOL000614	osthol	244.31	38.75	0.13	*Saposhnikoviae Radix*
MOL000615	delta-amorphene	204.39	17.95	0.08	*Saposhnikoviae Radix*
MOL000661	PENTACOSANOIC ACID	382.75	14.57	0.37	*Saposhnikoviae Radix*
MOL000667	1-hexanol	102.2	22.04	0.01	*Saposhnikoviae Radix*
MOL000668	PENTYLFURAN	138.23	54.59	0.02	*Saposhnikoviae Radix*
MOL000675	oleic acid	282.52	33.13	0.14	*Saposhnikoviae Radix*
MOL000676	DBP	278.38	64.54	0.13	*Saposhnikoviae Radix*
MOL000699	m-Cymol	134.24	48.85	0.02	*Saposhnikoviae Radix*
MOL000705	WLN: VH6	114.21	19.59	0.01	*Saposhnikoviae Radix*
MOL000708	WLN: VHR	106.13	32.63	0.01	*Saposhnikoviae Radix*
MOL000709	(S)-Matsutake alcohol	128.24	40.11	0.01	*Saposhnikoviae Radix*
MOL000716	trans-2-nonenal	140.25	19.18	0.02	*Saposhnikoviae Radix*
MOL000723	trans-2,4-decadienal	152.26	51.03	0.02	*Saposhnikoviae Radix*
MOL000724	Geranylacetone	194.35	18.66	0.04	*Saposhnikoviae Radix*
MOL000860	stearic acid	284.54	17.83	0.14	*Saposhnikoviae Radix*
MOL000879	methyl palmitate	270.51	18.09	0.12	*Saposhnikoviae Radix*
MOL000905	()-beta-Pinene	136.26	44.77	0.05	*Saposhnikoviae Radix*
MOL000914	(5S)-1-isopropyl-4-methylbicyclo[3.1.0]hex-3-ene	136.26	47.13	0.04	*Saposhnikoviae Radix*
MOL000918	2-NONANONE	142.27	8.51	0.02	*Saposhnikoviae Radix*
MOL000922	(R)-p-Menth-1-en-4-ol	154.28	32.16	0.03	*Saposhnikoviae Radix*
MOL000974	cuminal	148.22	38.29	0.03	*Saposhnikoviae Radix*
MOL001110	cis-beta-Ocimene	136.26	25.38	0.02	*Saposhnikoviae Radix*
MOL001114	2-[(1R)-2,2,3-trimethyl-1-cyclopent-3-enyl]ethanal	152.26	45.71	0.03	*Saposhnikoviae Radix*
MOL001121	19894-97-4	152.26	49.98	0.06	*Saposhnikoviae Radix*
MOL001123	muurolene	204.39	19.5	0.08	*Saposhnikoviae Radix*
MOL001129	l-Verbenone	150.24	50.66	0.06	*Saposhnikoviae Radix*
MOL001227	Decyl acetate	200.36	25.07	0.04	*Saposhnikoviae Radix*
MOL001245	(1S,5R)-7,7-dimethyl-4-bicyclo[3.1.1]hept-3-enecarboxaldehyde	150.24	41.25	0.06	*Saposhnikoviae Radix*
MOL001390	49070_FLUKA	222.41	85.51	0.12	*Saposhnikoviae Radix*
MOL001393	myristic acid	228.42	21.18	0.07	*Saposhnikoviae Radix*
MOL001396	PENTADECYLIC ACID	242.45	20.18	0.08	*Saposhnikoviae Radix*
MOL001487	FITONE	268.54	6.67	0.1	*Saposhnikoviae Radix*
MOL001494	Mandenol	308.56	42	0.19	*Saposhnikoviae Radix*
MOL001556	Isocaryophyllene	204.39	27.3	0.09	*Saposhnikoviae Radix*
MOL001578	Hypnon	120.16	48.19	0.02	*Saposhnikoviae Radix*
MOL001640	NON	172.3	26.74	0.03	*Saposhnikoviae Radix*
MOL001641	METHYL LINOLEATE	294.53	41.93	0.17	*Saposhnikoviae Radix*
MOL001863	METHYL ISOPALMITATE	270.51	9.42	0.11	*Saposhnikoviae Radix*
MOL001889	Methyl linolelaidate	294.53	41.93	0.17	*Saposhnikoviae Radix*
MOL001942	isoimperatorin	270.3	45.46	0.23	*Saposhnikoviae Radix*

MOL001949	panaxynol	244.41	42.44	0.1	*Saposhnikoviae Radix*
MOL002024	(1S,4R)-1,7,7-trimethylbicyclo[2.2.1]hept-2-ene	136.26	39.62	0.04	*Saposhnikoviae Radix*
MOL002033	cis-Thujopsene	204.39	56.43	0.12	*Saposhnikoviae Radix*
MOL002137	OCT	114.26	29.72	0.01	*Saposhnikoviae Radix*
MOL002138	p-Cymen-8-ol	150.24	32.26	0.03	*Saposhnikoviae Radix*
MOL002153	1H-Cycloprop(e)azulen-7-ol, decahydro-1,1,7-trimethyl-4-methylene-, (1aR-(1aalpha,4aalpha,7beta,7abeta,7balpha))-	220.39	82.33	0.12	*Saposhnikoviae Radix*
MOL002198	Heptan	100.23	41.8	0	*Saposhnikoviae Radix*
MOL002207	1(3H)-Isobenzofuranone, 3-butyl-3a,4,5,6-tetrahydro-, cis-(-)-	194.3	65.03	0.07	*Saposhnikoviae Radix*
MOL002378	UND	156.35	17.15	0.02	*Saposhnikoviae Radix*
MOL002379	PTL	86.15	59.53	0	*Saposhnikoviae Radix*
MOL002453	(-)-Comphene	136.26	34.98	0.04	*Saposhnikoviae Radix*
MOL002480	Methylbutenol	86.15	54.58	0.01	*Saposhnikoviae Radix*
MOL002520	.beta.-Fenchyl acetate, exo-	196.32	108.68	0.07	*Saposhnikoviae Radix*
MOL002972	(4S)-1-methyl-4-(6-methylhepta-1,5-dien-2-yl)cyclohexene	204.39	20.3	0.06	*Saposhnikoviae Radix*
MOL003040	Amylol	88.17	76.16	0	*Saposhnikoviae Radix*
MOL003050	nonanoic acid	158.27	40.51	0.02	*Saposhnikoviae Radix*
MOL003453	ZINC02571348	142.27	39.15	0.02	*Saposhnikoviae Radix*
MOL003493	naphthalene	128.18	27.55	0.03	*Saposhnikoviae Radix*
MOL003547	Azaron	208.28	38.39	0.06	*Saposhnikoviae Radix*
MOL003561	isopimpinellin	246.23	25.93	0.17	*Saposhnikoviae Radix*
MOL003588	Prangenidin	270.3	36.31	0.22	*Saposhnikoviae Radix*
MOL003937	Naphthalene, 1,2,3,4,4a,5,6,8a-octahydro-7-methyl-4-methylene-1-(1-methylethyl)-, (1alpha,4abeta,8aalpha)-	204.39	20.21	0.08	*Saposhnikoviae Radix*
MOL003951	Benzyl isovalerate	192.28	58.44	0.05	*Saposhnikoviae Radix*
MOL004284	2-Nonadecanone	282.57	14.38	0.14	*Saposhnikoviae Radix*
MOL004294	Perillen	150.24	19.74	0.03	*Saposhnikoviae Radix*
MOL004480	acetic acid	60.06	47.87	0	*Saposhnikoviae Radix*
MOL004667	fraxetin	208.18	23.04	0.09	*Saposhnikoviae Radix*
MOL004682	Methyl octylate	158.27	18.71	0.02	*Saposhnikoviae Radix*
MOL004687	2-octanone	128.24	19.29	0.01	*Saposhnikoviae Radix*
MOL004727	(1R,4aR,8aS)-1-isopropyl-7-methyl-4-methylene-2,3,4a,5,6,8a-hexahydro-1H-naphthalene	204.39	21.35	0.08	*Saposhnikoviae Radix*
MOL004775	Isobergapten	216.2	34.75	0.13	*Saposhnikoviae Radix*
MOL004783	nonane	128.29	29.23	0.01	*Saposhnikoviae Radix*
MOL004793	Marmesine	246.28	84.77	0.18	*Saposhnikoviae Radix*
MOL005274	Neohexane	86.2	37.81	0.01	*Saposhnikoviae Radix*
MOL005402	Methyl margarate	284.54	17.41	0.14	*Saposhnikoviae Radix*
MOL005723	Phytodolor	222.21	52.32	0.1	*Saposhnikoviae Radix*
MOL005786	Byakangelicin	334.35	27.67	0.35	*Saposhnikoviae Radix*
MOL007514	methyl icosa-11,14-dienoate	322.59	39.67	0.23	*Saposhnikoviae Radix*
MOL007545	lanceol	220.39	37.54	0.07	*Saposhnikoviae Radix*
MOL008743	1-Tridecyne	180.37	17.67	0.03	*Saposhnikoviae Radix*
MOL009356	Tectochrysin	268.28	9.57	0.2	*Saposhnikoviae Radix*

MOL010120	(1S,4R,4aR,8aR)-1-isopropyl-4,7-dimethyl-2,3,4,5,6,8a-hexahydro-1H-naphthalen-4a-ol	222.41	62.54	0.09	*Saposhnikoviae Radix*
MOL010244	(2S)-Flavanone	224.27	64.04	0.13	*Saposhnikoviae Radix*
MOL010705	2-Decenol	156.3	29.92	0.02	*Saposhnikoviae Radix*
MOL010947	(Z)-calamenene	202.37	17.75	0.08	*Saposhnikoviae Radix*
MOL011648	METHYL 10-OCTADECENOATE	296.55	31.9	0.17	*Saposhnikoviae Radix*
MOL011860	d-Dihydrocarvone	152.26	62.94	0.03	*Saposhnikoviae Radix*
MOL012462	2-Octenal, 2-butyl-	182.34	18.43	0.03	*Saposhnikoviae Radix*
MOL013077	Decursin	328.39	39.27	0.38	*Saposhnikoviae Radix*
MOL013176	2-[(2S)-7-oxo-2,3-dihydrofuro[4,5-g]chromen-2-yl]propan-2-yl (E)-2-methylbut-2-enoate	328.39	17.32	0.36	*Saposhnikoviae Radix*

MW: Molecule Weight; OB: Oral Bioavailability; DL: Drug-Likeness

Supplementary Table S2. Chemical information of 180 active compounds of Yihuo Huatan Formula

No.	InChIKey	Compound	Abbr. of Compounds	OB	DL	Herb
MOL000359	KZJWDPNRJALLNS-ZFVHJZABSA-N	sitosterol	127CR_CX	36.91	0.75	*Chuanxiong Rhizoma*
MOL000433	OVBPIULPVIDEAO-LBPRGKRZSA-N	FA	076CR	68.96	0.71	*Chuanxiong Rhizoma*
MOL001494	FMMOOAYVCKXGMF-MURFETPASA-N	Mandenol	110CR	42	0.19	*Chuanxiong Rhizoma*
MOL002135	ZTSNTUQTNQSIDC-UHFFFAOYSA-N	Myricanone	112CR	40.6	0.51	*Chuanxiong Rhizoma*
MOL002140	KFUCYPGCMLPUMT-UHFFFAOYSA-N	Perlolyrine	118CR_CX	65.95	0.27	*Chuanxiong Rhizoma*
MOL002157	HADAHVQGKBBPPW-NLQWGDCSSA-N	wallichilide	137CR	42.31	0.71	*Chuanxiong Rhizoma*
MOL000011	OCBGWPJNUZMLCA-NVXWUHKLSA-N	(2R,3R)-3-(4-hydroxy-3-methoxy-phenyl)-5-methoxy-2-methylol-2,3-dihydropyrano[5,6-h][1,4]benzodioxin-9-one	004SR	68.83	0.66	*Saposhnikoviae Radix*
MOL000173	XLTFNNCXVBYBSX-UHFFFAOYSA-N	wogonin	138SR	30.68	0.23	*Saposhnikoviae Radix*
MOL000358	KZJWDPNRJALLNS-VJSFXXLFSA-N	beta-sitosterol	048SR	36.91	0.75	*Saposhnikoviae Radix*
MOL000359	KZJWDPNRJALLNS-ZFVHJZABSA-N	sitosterol	127SR	36.91	0.75	*Saposhnikoviae Radix*
MOL001494	FMMOOAYVCKXGMF-MURFETPASA-N	Mandenol	110SR	42	0.19	*Saposhnikoviae Radix*
MOL001941	OLOOJGVNMBJLLR-UHFFFAOYSA-N	Ammidin	041SR	34.55	0.22	*Saposhnikoviae Radix*
MOL001942	IGWDEVSBEKYORK-UHFFFAOYSA-N	isoimperatorin	097SR	45.46	0.23	*Saposhnikoviae Radix*
MOL002644	BMLZFLQMBMYVHG-UHFFFAOYSA-N	Phellopterin	119SR	40.19	0.28	*Saposhnikoviae Radix*
MOL003588	KDXVVZMYSLWJMA-UHFFFAOYSA-N	Prangenidin	124SR	36.31	0.22	*Saposhnikoviae Radix*
MOL007514	GWJCFAOQCNNFAM-ZDVGBALWSA-N	methyl icosa-11,14-dienoate	111SR	39.67	0.23	*Saposhnikoviae Radix*
MOL011730	HVGQWHMSVYODLJ-GFCCVEGCSA-N	11-hydroxy-sec-o-beta-d-glucosylhamaudol_qt	015SR	50.24	0.27	*Saposhnikoviae Radix*
MOL011732	PNTWXEIQXBRCPS-ODRFPHDWSA-N	anomalin	044SR	59.65	0.66	*Saposhnikoviae Radix*
MOL011737	YJHFCJWBLAJYRN-LBPRGKRZSA-N	divaricatacid	069SR	87	0.32	*Saposhnikoviae Radix*
MOL011740	DJEFKGJBMQEDCR-CQSZACIVSA-N	divaricatol	070SR	31.65	0.38	*Saposhnikoviae Radix*
MOL011747	KKDRQZCQNSHBHY-MGDKSHQASA-N	ledebouriellol	103SR	32.05	0.51	*Saposhnikoviae Radix*
MOL011749	BMLZFLQMBMYVHG-UHFFFAOYSA-O	phelloptorin	120SR	43.39	0.28	*Saposhnikoviae Radix*
MOL011753	DGFLRNOCLJGHLY-ZDUSSCGKSA-N	5-O-Methylvisamminol	030SR	37.99	0.25	*Saposhnikoviae Radix*
MOL013077	CUKSFECWKQBVED-INIZCTEOSA-N	Decursin	063SR	39.27	0.38	*Saposhnikoviae Radix*
MOL011753	DGFLRNOCLJGHLY-ZDUSSCGKSA-N	5-O-Methylvisamminol	030SR	37.99	0.25	*Saposhnikoviae Radix*

MOL001494	FMMOOAYVCKXGMF-MURFETPASA-N	Mandenol	110TKM	42	0.19	*Trichosanthes Kirilowii Maxim*
MOL002881	MBNGWHIJMBWFHU-UHFFFAOYSA-N	Diosmetin	068TKM	31.14	0.27	*Trichosanthes Kirilowii Maxim*
MOL004355	JZVFJDZBLUFKCA-FXIAWGAOSA-N	Spinasterol	129TKM	42.98	0.76	*Trichosanthes Kirilowii Maxim*
MOL005530	RRRSSAVLTCVNIQ-UHFFFAOYSA-N	Hydroxygenkwanin	093TKM	36.47	0.27	*Trichosanthes Kirilowii Maxim*
MOL006756	YSKVBPGQYRAUQO-UZSYLJJSSA-N	Schottenol	126TKM	37.42	0.75	*Trichosanthes Kirilowii Maxim*
MOL007165	WSPRAEIJBDUDRX-MPJPVBFLSA-N	10α-cucurbita-5,24-diene-3β-ol	013TKM	44.02	0.74	*Trichosanthes Kirilowii Maxim*
MOL007171	WDJWPKSFUUKJFX-CRUZSCCSSA-N	5-dehydrokarounidiol	029TKM	30.23	0.77	*Trichosanthes Kirilowii Maxim*
MOL007172	LGSJWAHGGJDXIU-RXGLLHQPSA-N	7-oxo-dihydrokaro-unidiol	035TKM	36.85	0.75	*Trichosanthes Kirilowii Maxim*
MOL007179	JYYFMIOPGOFNPK-AGRJPVHOSA-N	Linolenic acid ethyl ester	105TKM	46.1	0.2	*Trichosanthes Kirilowii Maxim*
MOL007180	WIGIZIANZCJQQY-RUCARUNLSA-N	vitamin-e	136TKM	32.29	0.7	*Trichosanthes Kirilowii Maxim*
MOL000358	KZJWDPNRJALLNS-VJSFXXLFSA-N	beta-sitosterol	048ATT	36.91	0.75	*Arum Ternatum Thunb.*
MOL000449	HCXVJBMSMIARIN-PHZDYDNGSA-N	Stigmasterol	132ATT	43.83	0.76	*Arum Ternatum Thunb.*
MOL000519	PKEGICXVZMKJPR-JTQLQIEISA-N	coniferin	059ATT	31.11	0.32	*Arum Ternatum Thunb.*
MOL001755	RUVUHIUYGJBLGI-XJZKHKOHSA-N	24-Ethylcholest-4-en-3-one	021ATT	36.08	0.76	*Arum Ternatum Thunb.*
MOL002670	JTZZGWPIBBTYNE-FKIZINRSSA-N	Cavidine	053ATT	35.64	0.81	*Arum Ternatum Thunb.*
MOL002714	FXNFHKRTJBSTCS-UHFFFAOYSA-N	baicalein	045ATT	33.52	0.21	*Arum Ternatum Thunb.*
MOL002776	IKIIZLYTISPENI-ZFORQUDYSA-N	Baicalin	046ATT	40.12	0.75	*Arum Ternatum Thunb.*
MOL003578	ONQRKEUAIJMULO-YBXTVTTCSA-N	Cycloartenol	061ATT	38.69	0.78	*Arum Ternatum Thunb.*
MOL005030	BITHHVVYSMSWAG-KTKRTIGZSA-N	gondoic acid	090ATT	30.7	0.2	*Arum Ternatum Thunb.*
MOL006936	BYHLDPSVWJCXEG-ZDVGBALWSA-N	10,13-eicosadienoic	012ATT	39.99	0.2	*Arum Ternatum Thunb.*
MOL006957	GRWVBLRIPRGGPD-HOTGVXAUSA-N	(3S,6S)-3-(benzyl)-6-(4-hydroxybenzyl)piperazine-2,5-quinone	006ATT	46.89	0.27	*Arum Ternatum Thunb.*
MOL006967	UBORTCNDUKBEOP-UUOKFMHZSA-N	beta-D-Ribofuranoside, xanthine-9	047ATT	44.72	0.21	*Arum Ternatum Thunb.*
MOL000022	CQDVFBMTEZFKKY-PUYRJQRLSA-N	14-acetyl-12-senecioyl-2E,8Z,10E-atractylentriol	016AMK	63.37	0.3	*Atractylodes Macrocephala Koidz.*
MOL000033	KLEXDBGYSOIREE-UIFQYPGESA-N	(3S,8S,9S,10R,13R,14S,17R)-10,13-dimethyl-17-[(2R,5S)-5-propan-2-yloctan-2-yl]-2,3,4,7,8,9,11,12,14,15,16,17-dodecahydro-1H-cyclopenta[a]phenanthren-3-ol	007AMK	36.23	0.78	*Atractylodes Macrocephala Koidz.*
MOL000049	YYGOPZCCRBWMBK-ZOBUZTSGSA-N	3β-acetoxyatractylone	026AMK	54.07	0.22	*Atractylodes Macrocephala Koidz.*
MOL000072	JATCNILBQFTWFJ-XKQJLSEDSA-N	8β-ethoxy atractylenolide Ⅲ	038AMK	35.95	0.21	*Atractylodes Macrocephala Koidz.*

MOL000359	KZJWDPNRJALLNS-ZFVHJZABSA-N	sitosterol	127CR_CP	36.91	0.75	*Citrus Reticulata*
MOL004328	FTVWIRXFELQLPI-ZDUSSCGKSA-N	naringenin	113CR	59.29	0.21	*Citrus Reticulata*
MOL005100	AIONOLUJZLIMTK-CQSZACIVSA-N	5,7-dihydroxy-2-(3-hydroxy-4-methoxyphenyl)chroman-4-one	028CR	47.74	0.27	*Citrus Reticulata*
MOL005815	LTRBUBSPQISFFL-CQSZACIVSA-N	Citromitin	057CR	86.9	0.51	*Citrus Reticulata*
MOL005828	MRIAQLRQZPPODS-UHFFFAOYSA-N	nobiletin	114CR	61.67	0.52	*Citrus Reticulata*
MOL000006	IQPNAANSBPBGFQ-UHFFFAOYSA-N	luteolin	107CR	36.16	0.25	*Codonopsis Radix*
MOL000449	HCXVJBMSMIARIN-PHZDYDNGSA-N	Stigmasterol	132CR	43.83	0.76	*Codonopsis Radix*
MOL001006	JZVFJDZBLUFKCA-INYURWPISA-N	poriferasta-7,22E-dien-3beta-ol	123CR	42.98	0.76	*Codonopsis Radix*
MOL002140	KFUCYPGCMLPUMT-UHFFFAOYSA-N	Perlolyrine	118CR_DS	65.95	0.27	*Codonopsis Radix*
MOL002879	IJFPVINAQGWBRJ-UHFFFAOYSA-N	Diop	065CR	43.59	0.39	*Codonopsis Radix*
MOL003036	HCXVJBMSMIARIN-NKMAIEQZSA-N	ZINC03978781	142CR	43.83	0.76	*Codonopsis Radix*
MOL003896	XRGWZIGGCNSFRY-UHFFFAOYSA-N	7-Methoxy-2-methyl isoflavone	033CR	42.56	0.2	*Codonopsis Radix*
MOL004355	JZVFJDZBLUFKCA-FXIAWGAOSA-N	Spinasterol	129CR	42.98	0.76	*Codonopsis Radix*
MOL005321	RFWULRHBGYKEEZ-UHFFFAOYSA-N	Frutinone A	078CR	65.9	0.34	*Codonopsis Radix*
MOL006774	YSKVBPGQYRAUQO-UBCZCFGNSA-N	stigmast-7-enol	131CR	37.42	0.75	*Codonopsis Radix*
MOL007059	RUJKJFRMCYQMLH-ZDUSSCGKSA-N	3-beta-Hydroxymethyllenetanshiquinone	024CR	32.16	0.41	*Codonopsis Radix*
MOL007514	GWJCFAOQCNNFAM-ZDVGBALWSA-N	methyl icosa-11,14-dienoate	111CR	39.67	0.23	*Codonopsis Radix*
MOL008393	YVFWOWYGCXXKFY-IICLVWGGSA-N	7-(beta-Xylosyl)cephalomannine_qt	032CR	38.33	0.29	*Codonopsis Radix*
MOL008397	QUGZOXCXHGEACS-ZFEQYVDASA-N	Daturilin	062CR	50.37	0.77	*Codonopsis Radix*
MOL008400	DXYUAIFZCFRPTH-UHFFFAOYSA-N	glycitein	089CR	50.48	0.24	*Codonopsis Radix*
MOL008407	XWDAKKDQJHVMKG-LPJPOILFSA-N	(8S,9S,10R,13R,14S,17R)-17-[(E,2R,5S)-5-ethyl-6-methylhept-3-en-2-yl]-10,13-dimethyl-1,2,4,7,8,9,11,12,14,15,16,17-dodecahydrocyclopenta[a]phenanthren-3-one	009CR	45.4	0.76	*Codonopsis Radix*
MOL008411	FALAMCOLIJTCTR-XAVCKHEUSA-N	11-Hydroxyrankinidine	014CR	40	0.66	*Codonopsis Radix*
MOL000358	KZJWDPNRJALLNS-VJSFXXLFSA-N	beta-sitosterol	048COS	36.91	0.75	*Cornus Officinalis Sieb. Et Zucc.*
MOL000359	KZJWDPNRJALLNS-ZFVHJZABSA-N	sitosterol	127COS	36.91	0.75	*Cornus Officinalis Sieb. Et Zucc.*
MOL000449	HCXVJBMSMIARIN-PHZDYDNGSA-N	Stigmasterol	132COS	43.83	0.76	*Cornus Officinalis Sieb. Et Zucc.*
MOL001494	FMMOOAYVCKXGMF-MURFETPASA-N	Mandenol	110COS	42	0.19	*Cornus Officinalis Sieb. Et Zucc.*
MOL001495	JYYFMIOPGOFNPK-XSHSMGBESA-N	Ethyl linolenate	074COS	46.1	0.2	*Cornus Officinalis Sieb. Et Zucc.*
MOL001771	KZJWDPNRJALLNS-FBZNIEFRSA-N	poriferast-5-en-3beta-ol	122COS	36.91	0.75	*Cornus Officinalis Sieb. Et Zucc.*

MOL002879	IJFPVINAQGWBRJ-UHFFFAOYSA-N	Diop	065COS	43.59	0.39	*Cornus Officinalis Sieb. Et Zucc.*
MOL002883	LVGKNOAMLMIIKO-QXMHVHEDSA-N	Ethyl oleate (NF)	075COS	32.4	0.19	*Cornus Officinalis Sieb. Et Zucc.*
MOL003137	DLVLXOYLQKCAME-DGHBBABESA-N	Leucanthoside	104COS	32.12	0.78	*Cornus Officinalis Sieb. Et Zucc.*
MOL005481	IMXDCJPVYKXJPD-FMOJUEAUSA-N	2,6,10,14,18-pentamethylicosa-2,6,10,14,18-pentaene	019COS	33.4	0.24	*Cornus Officinalis Sieb. Et Zucc.*
MOL005503	JIUGZSYPFREDLG-HXUWFJFHSA-N	Cornudentanone	060COS	39.66	0.33	*Cornus Officinalis Sieb. Et Zucc.*
MOL005530	RRRSSAVLTCVNIQ-UHFFFAOYSA-N	Hydroxygenkwanin	093COS	36.47	0.27	*Cornus Officinalis Sieb. Et Zucc.*
MOL005531	PBSOJKPTQWWJJD-XECVOWJVSA-N	Telocinobufagin	133COS	69.99	0.79	*Cornus Officinalis Sieb. Et Zucc.*
MOL008457	GRTOGORTSDXSFK-DLLGKBFGSA-N	Tetrahydroalstonine	134COS	32.42	0.81	*Cornus Officinalis Sieb. Et Zucc.*
MOL000006	IQPNAANSBPBGFQ-UHFFFAOYSA-N	luteolin	107EH	36.16	0.25	*Epimrdii Herba*
MOL000098	REFJWTPEDVJJIY-UHFFFAOYSA-N	quercetin	125EH	46.43	0.28	*Epimrdii Herba*
MOL000359	KZJWDPNRJALLNS-ZFVHJZABSA-N	sitosterol	127EH	36.91	0.75	*Epimrdii Herba*
MOL000422	IYRMWMYZSQPJKC-UHFFFAOYSA-N	kaempferol	102EH	41.88	0.24	*Epimrdii Herba*
MOL000622	VHFXPBHLQOPQHJ-ABBQYLIMSA-N	Magnograndiolide	108EH	63.71	0.19	*Epimrdii Herba*
MOL001510	SGNBVLSWZMBQTH-ZRUUVFCLSA-N	24-epicampesterol	020EH	37.58	0.71	*Epimrdii Herba*
MOL001645	KFXARGMQYWECBV-ZDVGBALWSA-N	Linoleyl acetate	106EH	42.1	0.2	*Epimrdii Herba*
MOL001771	KZJWDPNRJALLNS-FBZNIEFRSA-N	poriferast-5-en-3beta-ol	122EH	36.91	0.75	*Epimrdii Herba*
MOL001792	FURUXTVZLHCCNA-AWEZNQCLSA-N	DFV	064EH	32.76	0.18	*Epimrdii Herba*
MOL003044	SCZVLDHREVKTSH-UHFFFAOYSA-N	Chryseriol	056EH	35.85	0.27	*Epimrdii Herba*
MOL003542	NADCVNHITZNGJU-UHFFFAOYSA-N	8-Isopentenyl-kaempferol	037EH	38.04	0.39	*Epimrdii Herba*
MOL004367	BVHIKUCXNBQDEM-XMCHAPAWSA-N	olivil	115EH	62.23	0.41	*Epimrdii Herba*
MOL004373	TUUXBSASAQJECY-UHFFFAOYSA-N	Anhydroicaritin	042EH	45.41	0.44	*Epimrdii Herba*
MOL004380	VFNBFPRWBICVGZ-JXFKEZNVSA-N	C-Homoerythrinan, 1,6-didehydro-3,15,16-trimethoxy-, (3.beta.)-	055EH	39.14	0.49	*Epimrdii Herba*
MOL004382	ZAUWPDSVLSOCDG-SFHVURJKSA-N	Yinyanghuo A	139EH	56.96	0.77	*Epimrdii Herba*
MOL004384	GPXYBBZISZKRAH-UHFFFAOYSA-N	Yinyanghuo C	140EH	45.67	0.5	*Epimrdii Herba*
MOL004386	FIKLOAGOJKGOFT-UHFFFAOYSA-N	Yinyanghuo E	141EH	51.63	0.55	*Epimrdii Herba*
MOL004388	FUBYUUKASUJMSZ-UHFFFAOYSA-N	6-hydroxy-11,12-dimethoxy-2,2-dimethyl-1,8-dioxo-2,3,4,8-tetrahydro-1H-isochromeno[3,4-h]isoquinolin-2-ium	031EH	60.64	0.66	*Epimrdii Herba*
MOL004391	FMPOBQILEGSRSJ-UHFFFAOYSA-N	8-(3-methylbut-2-enyl)-2-phenyl-chromone	036EH	48.54	0.25	*Epimrdii Herba*
MOL004394	TZJALUIVHRYQQB-YPRONELTSA-N	Anhydroicaritin-3-O-alpha-L-rhamnoside	043EH	41.58	0.61	*Epimrdii Herba*

MOL004396	DFUOJBWSSSODTR-SJCJKPOMSA-N	1,2-bis(4-hydroxy-3-methoxyphenyl)propan-1,3-diol	010EH	52.31	0.22	*Epimrdii Herba*
MOL004425	TZJALUIVHRYQQB-XLRXWWTNSA-N	Icariin	094EH	41.58	0.61	*Epimrdii Herba*
MOL004427	HNMHZSRVQJZGPQ-PUIBNRJISA-N	Icariside A7	095EH	31.91	0.86	*Epimrdii Herba*
MOL000033	KLEXDBGYSOIREE-UIFQYPGESA-N	(3S,8S,9S,10R,13R,14S,17R)-10,13-dimethyl-17-[(2R,5S)-5-propan-2-yloctan-2-yl]-2,3,4,7,8,9,11,12,14,15,16,17-dodecahydro-1H-cyclopenta[a]phenanthren-3-ol	007HMM	36.23	0.78	*Hedysarum Multijugum Maxim.*
MOL000098	REFJWTPEDVJJIY-UHFFFAOYSA-N	quercetin	125HMM	46.43	0.28	*Hedysarum Multijugum Maxim.*
MOL000211	QGJZLNKBHJESQX-FZFNOLFKSA-N	Mairin	109HMM	55.38	0.78	*Hedysarum Multijugum Maxim.*
MOL000239	BJBUTJQYZDYRMJ-UHFFFAOYSA-N	Jaranol	100HMM	50.83	0.29	*Hedysarum Multijugum Maxim.*
MOL000296	KZJWDPNRJALLNS-CQXWNKEUSA-N	hederagenin	092HMM	36.91	0.75	*Hedysarum Multijugum Maxim.*
MOL000354	IZQSVPBOUDKVDZ-UHFFFAOYSA-N	isorhamnetin	099HMM	49.6	0.31	*Hedysarum Multijugum Maxim.*
MOL000371	RFFNFQZKHNKOPO-BBRMVZONSA-N	3,9-di-O-methylnissolin	023HMM	53.74	0.48	*Hedysarum Multijugum Maxim.*
MOL000378	BLHQCBJSTMDZQA-LBPRGKRZSA-N	7-O-methylisomucronulatol	034HMM	74.69	0.3	*Hedysarum Multijugum Maxim.*
MOL000379	PCIXSTFFMHVOMF-PBGSHFJYSA-N	9,10-dimethoxypterocarpan-3-O-β-D-glucoside	039HMM	36.74	0.92	*Hedysarum Multijugum Maxim.*
MOL000380	UOVGCLXUTLXAEC-WFASDCNBSA-N	(6aR,11aR)-9,10-dimethoxy-6a,11a-dihydro-6H-benzofurano[3,2-c]chromen-3-ol	008HMM	64.26	0.42	*Hedysarum Multijugum Maxim.*
MOL000387	JMZOMFYRADAWOG-UHFFFAOYSA-N	Bifendate	049HMM	31.1	0.67	*Hedysarum Multijugum Maxim.*
MOL000392	HKQYGTCOTHHOMP-UHFFFAOYSA-N	formononetin	077HMM	69.67	0.21	*Hedysarum Multijugum Maxim.*
MOL000417	ZZAJQOPSWWVMBI-UHFFFAOYSA-N	Calycosin	050HMM	47.75	0.24	*Hedysarum Multijugum Maxim.*
MOL000422	IYRMWMYZSQPJKC-UHFFFAOYSA-N	kaempferol	102HMM	41.88	0.24	*Hedysarum Multijugum Maxim.*
MOL000433	OVBPIULPVIDEAO-LBPRGKRZSA-N	FA	076HMM	68.96	0.71	*Hedysarum Multijugum Maxim.*
MOL000439	NHOPAJCVMDIGBN-MEPKZADGSA-N	isomucronulatol-7,2'-di-O-glucosiole	098HMM	49.28	0.62	*Hedysarum Multijugum Maxim.*
MOL000442	RVGZSUMTFIEORY-UHFFFAOYSA-N	1,7-Dihydroxy-3,9-dimethoxy pterocarpene	011HMM	39.05	0.48	*Hedysarum Multijugum Maxim.*
MOL000296	KZJWDPNRJALLNS-CQXWNKEUSA-N	hederagenin	092PS	36.91	0.75	*Persicae Semen*
MOL000358	KZJWDPNRJALLNS-VJSFXXLFSA-N	beta-sitosterol	048PS	36.91	0.75	*Persicae Semen*
MOL000493	SGNBVLSWZMBQTH-XQBXDOQXSA-N	campesterol	052PS	37.58	0.71	*Persicae Semen*

MOL001323	LPZCCMIISIBREI-JXMPMKKESA-N	Sitosterol alpha1	128PS	43.28	0.78	*Persicae Semen*
MOL001328	VEVVCHBAZIRPBB-MTVNJVDJSA-N	2,3-didehydro GA70	017PS	63.29	0.5	*Persicae Semen*
MOL001329	IZLFTLDLFQILLE-OBDJNFEBSA-N	2,3-didehydro GA77	018PS	88.08	0.53	*Persicae Semen*
MOL001340	WKDIZCSRKTURDM-YGNOGLJPSA-N	GA120	079PS	84.85	0.45	*Persicae Semen*
MOL001342	BBUUSKRDUVMSSS-CIPMSPFZSA-N	GA121-isolactone	080PS	72.7	0.54	*Persicae Semen*
MOL001344	BBUUSKRDUVMSSS-LVCOZDEDSA-N	GA122-isolactone	081PS	88.11	0.54	*Persicae Semen*
MOL001349	VNCQCPQAMDQEBY-YTJHIPEWSA-N	4a-formyl-7alpha-hydroxy-1-methyl-8-methylidene-4aalpha,4bbeta-gibbane-1alpha,10beta-dicarboxylic acid	027PS	88.6	0.46	*Persicae Semen*
MOL001351	KSBJAONOPKRVRR-LTTRRGSQSA-N	Gibberellin A44	088PS	101.61	0.54	*Persicae Semen*
MOL001352	ZGHAVKULRAPSKM-LYQBYBLHSA-N	GA54	082PS	64.21	0.53	*Persicae Semen*
MOL001353	XPVLCCOOMVYREG-KDOUOODKSA-N	GA60	083PS	93.17	0.53	*Persicae Semen*
MOL001355	RLZBXKKKLIYURW-ZUJRJSPDSA-N	GA63	084PS	65.54	0.54	*Persicae Semen*
MOL001358	SEEGHKWOBVVBTQ-NFMPGMCNSA-N	gibberellin 7	087PS	73.8	0.5	*Persicae Semen*
MOL001360	BQOOPWCLYXZXSL-OBDJNFEBSA-N	GA77	085PS	87.89	0.53	*Persicae Semen*
MOL001361	DBCALXGMWSCZIP-BPDLVRBNSA-N	GA87	086PS	68.85	0.57	*Persicae Semen*
MOL001368	BMRSEYFENKXDIS-OALXIOMQSA-N	3-O-p-coumaroylquinic acid	025PS	37.63	0.29	*Persicae Semen*
MOL000273	XSLKAKROJKMHIT-WIUKAADNSA-N	(2R)-2-[(3S,5R,10S,13R,14R,16R,17R)-3,16-dihydroxy-4,4,10,13,14-pentamethyl-2,3,5,6,12,15,16,17-octahydro-1H-cyclopenta[a]phenanthren-17-yl]-6-methylhept-5-enoic acid	003PCW	30.93	0.81	*Poria Cocos(Schw.) Wolf.*
MOL000275	NBSBUIQBEPROBM-QPOZJWKMSA-N	trametenolic acid	135PCW	38.71	0.8	*Poria Cocos(Schw.) Wolf.*
MOL000279	ARXHRTZAVQOQEU-BRVLHLJYSA-N	Cerevisterol	054PCW	37.96	0.77	*Poria Cocos(Schw.) Wolf.*
MOL000282	QOXPZVASXWSKKU-UEIWAABPSA-N	ergosta-7,22E-dien-3beta-ol	072PCW	43.51	0.72	*Poria Cocos(Schw.) Wolf.*
MOL000283	PIENIXCJUGJKPI-JYJMTLRPSA-N	Ergosterol peroxide	073PCW	40.36	0.81	*Poria Cocos(Schw.) Wolf.*
MOL000296	KZJWDPNRJALLNS-CQXWNKEUSA-N	hederagenin	092PCW	36.91	0.75	*Poria Cocos(Schw.) Wolf.*
MOL000358	KZJWDPNRJALLNS-VJSFXXLFSA-N	beta-sitosterol	048RPR	36.91	0.75	*Radix Paeoniae Rubra*
MOL000359	KZJWDPNRJALLNS-ZFVHJZABSA-N	sitosterol	127RPR	36.91	0.75	*Radix Paeoniae Rubra*
MOL000449	HCXVJBMSMIARIN-PHZDYDNGSA-N	Stigmasterol	132RPR	43.83	0.76	*Radix Paeoniae Rubra*
MOL000492	PFTAWBLQPZVEMU-DZGCQCFKSA-N	(+)-catechin	002RPR	54.83	0.24	*Radix Paeoniae Rubra*
MOL001002	AFSDNFLWKVMVRB-UHFFFAOYSA-N	ellagic acid	071RPR	43.06	0.43	*Radix Paeoniae Rubra*
MOL001918	BANPEMKDTXIFRE-GHVWTTSJSA-N	paeoniflorgenone	116RPR	87.59	0.37	*Radix Paeoniae Rubra*

MOL001924	YKRGDOXKVOZESV-WRJNSLSBSA-N	paeoniflorin	117RPR	53.87	0.79	*Radix Paeoniae Rubra*
MOL002714	FXNFHKRTJBSTCS-UHFFFAOYSA-N	baicalein	045RPR	33.52	0.21	*Radix Paeoniae Rubra*
MOL002776	IKIIZLYTISPENI-ZFORQUDYSA-N	Baicalin	046RPR	40.12	0.75	*Radix Paeoniae Rubra*
MOL002883	LVGKNOAMLMIIKO-QXMHVHEDSA-N	Ethyl oleate (NF)	075RPR	32.4	0.19	*Radix Paeoniae Rubra*
MOL004355	JZVFJDZBLUFKCA-FXIAWGAOSA-N	Spinasterol	129RPR	42.98	0.76	*Radix Paeoniae Rubra*
MOL005043	SGNBVLSWZMBQTH-PODYLUTMSA-N	campest-5-en-3beta-ol	051RPR	37.58	0.71	*Radix Paeoniae Rubra*
MOL006992	ITWRRUUKFUXICF-CVEARBPZSA-N	(2R,3R)-4-methoxyl-distylin	005RPR	59.98	0.3	*Radix Paeoniae Rubra*
MOL006999	YSKVBPGQYRAUQO-NPAMSQCVSA-N	stigmast-7-en-3-ol	130RPR	37.42	0.75	*Radix Paeoniae Rubra*
MOL000359	KZJWDPNRJALLNS-ZFVHJZABSA-N	sitosterol	127RRP	36.91	0.75	*Rehmanniae Radix Praeparata*
MOL000449	HCXVJBMSMIARIN-PHZDYDNGSA-N	Stigmasterol	132RRP	43.83	0.76	*Rehmanniae Radix Praeparata*
MOL000322	VDYACOATPFOZIO-UBWHGVKJSA-N	Kadsurenone	101RD	54.72	0.38	*Rhizoma Dioscoreae*
MOL000449	HCXVJBMSMIARIN-PHZDYDNGSA-N	Stigmasterol	132RD	43.83	0.76	*Rhizoma Dioscoreae*
MOL000493	SGNBVLSWZMBQTH-XQBXDOQXSA-N	campesterol	052RD	37.58	0.71	*Rhizoma Dioscoreae*
MOL000546	WQLVFSAGQJTQCK-VKROHFNGSA-N	diosgenin	067RD	80.88	0.81	*Rhizoma Dioscoreae*
MOL000953	HVYWMOMLDIMFJA-DPAQBDIFSA-N	CLR	058RD	37.87	0.68	*Rhizoma Dioscoreae*
MOL001559	WHAAPCGHVWVUEX-GGWOSOGESA-N	piperlonguminine	121RD	30.71	0.18	*Rhizoma Dioscoreae*
MOL001736	CXQWRCVTCMQVQX-CABCVRRESA-N	(-)-taxifolin	001RD	60.51	0.27	*Rhizoma Dioscoreae*
MOL005430	LNCSXXRCEFAYFK-VOCPSOEZSA-N	hancinone C	091RD	59.05	0.39	*Rhizoma Dioscoreae*
MOL005435	SGNBVLSWZMBQTH-CVAXJGOOSA-N	24-Methylcholest-5-enyl-3belta-O-glucopyranoside_qt	022RD	37.58	0.72	*Rhizoma Dioscoreae*
MOL005440	OSELKOCHBMDKEJ-WGMIZEQOSA-N	Isofucosterol	096RD	43.78	0.76	*Rhizoma Dioscoreae*
MOL005458	VIDMMZSEYKFBPP-VSDWVOOISA-N	Dioscoreside C_qt	066RD	36.38	0.87	*Rhizoma Dioscoreae*
MOL005465	HVXHJNVYRXRHNX-UHFFFAOYSA-N	AIDS180907	040RD	45.33	0.77	*Rhizoma Dioscoreae*

OB: Oral Bioavailability; DL: Drug-Likeness

Supplementary Table S3A. 128 candidate compounds of Yihuo Huatan Formula

Abbr. of Compounds	Degree
125HMM	148
125EH	148
102HMM	62
102EH	62
107CR	56
107EH	56
034HMM	45
138SR	45
033CR	43
055EH	38
077HMM	38
042EH	37
045ATT	37
045RPR	37
048ATT	37
048RPR	37
048PS	37
048COS	37
048SR	37
099HMM	36
113CR	35
114CR	35
132CR	31
132RD	31
132ATT	31
132RPR	31
132RRP	31
132COS	31
036EH	30
037EH	29
053ATT	28
134COS	28
101RD	27
112CR	25
030SR	24
023HMM	23
089CR	23
008HMM	22
050HMM	22
091RD	22
059ATT	21
092HMM	21
092PCW	21
092PS	21
071RPR	19

004SR	18
056EH	18
024CR	17
026AMK	16
063SR	16
067RD	16
070SR	16
078CR	16
124SR	15
103SR	14
040RD	13
100HMM	13
119SR	12
010EH	11
064EH	11
121RD	11
140EH	11
141EH	11
002RPR	10
028CR	10
057CR	10
068TKM	10
093TKM	10
093COS	10
139EH	9
025PS	8
041SR	8
079PS	8
096RD	8
015SR	7
017PS	7
044SR	7
049HMM	7
087PS	7
031EH	6
052RD	6
052PS	6
082PS	6
005RPR	5
018PS	5
038AMK	5
069SR	5
083PS	5
084PS	5
120SR	5
128PS	5
001RD	4
006ATT	4
011HMM	4

014CR	4
106EH	4
108EH	4
115EH	4
117RPR	4
118CR_DS	4
118CR_CX	4
137CR	4
039HMM	3
058RD	3
060COS	3
065CR	3
065COS	3
076HMM	3
076CR	3
085PS	3
088PS	3
095EH	3
104COS	3
110TKM	3
110CR	3
110COS	3
110SR	3
123CR	3
127CR_CP	3
127RPR	3
127CR_CX	3
127RRP	3
127COS	3
127EH	3
127SR	3
129CR	3
129TKM	3
129RPR	3
142CR	3
003PCW	2
009CR	2
012ATT	2
013TKM	2
020EH	2
021ATT	2
027PS	2
046ATT	2
046RPR	2
047ATT	2
066RD	2
074COS	2
086PS	2
090ATT	2

105TKM	2
122COS	2
122EH	2
126TKM	2
131CR	2
133COS	2
007HMM	1
007AMK	1
016AMK	1
019COS	1
022RD	1
029TKM	1
032CR	1
035TKM	1
043EH	1
051RPR	1
054PCW	1
061ATT	1
062CR	1
072PCW	1
073PCW	1
075RPR	1
075COS	1
080PS	1
081PS	1
094EH	1
097SR	1
098HMM	1
109HMM	1
111CR	1
111SR	1
116RPR	1
130RPR	1
135PCW	1
136TKM	1

Supplementary Table S3B. The corresponding targets of 128 candidate compounds

Target ID	Target Name	Degree
TAR00094	Prostaglandin G/H synthase 2	108
TAR03276	Nuclear receptor coactivator 2	91
TAR00006	Prostaglandin G/H synthase 1	70
TAR00444	Heat shock protein HSP 90-alpha	58
TAR00209	Progesterone receptor	54
TAR00070	Sodium channel protein type 5 subunit alpha	46
TAR00309	Gamma-aminobutyric-acid receptor subunit alpha-1	45
TAR00332	Dipeptidyl peptidase 4	43
TAR00699	cAMP-dependent protein kinase catalytic subunit alpha	42
TAR00261	Beta-2 adrenergic receptor	41
TAR03907	Calmodulin	40
TAR00158	Retinoic acid receptor RXR-alpha	39
TAR00048	Androgen receptor	39
TAR00038	Muscarinic acetylcholine receptor M1	39
TAR02928	Trypsin-1	38
TAR00017	Prothrombin	37
TAR00252	Mineralocorticoid receptor	34
TAR00046	Estrogen receptor	32
TAR00003	Nitric oxide synthase, inducible	32
TAR00216	Alpha-1B adrenergic receptor	32
TAR00016	Muscarinic acetylcholine receptor M3	32
TAR00078	Peroxisome proliferator-activated receptor gamma	31
TAR00172	cGMP-inhibited 3',5'-cyclic phosphodiesterase A	29
TAR00491	Phosphatidylinositol-4,5-bisphosphate 3-kinase catalytic subunit gamma isoform	28
TAR00287	DNA topoisomerase 2-alpha	28
TAR00482	Cell division protein kinase 2	26
TAR03279	Nuclear receptor coactivator 1	24
TAR00079	Coagulation factor X	23
TAR04387	Neuronal acetylcholine receptor subunit alpha-7	23
TAR00165	Acetylcholinesterase	22
TAR00210	Muscarinic acetylcholine receptor M2	22
TAR02966	Proto-oncogene serine/threonine-protein kinase Pim-1	21
TAR00037	Potassium voltage-gated channel subfamily H member 2	20
TAR00422	Glycogen synthase kinase-3 beta	18
TAR00095	Nitric-oxide synthase, endothelial	17
TAR00191	Alpha-1A adrenergic receptor	17
TAR00200	Gamma-aminobutyric-acid receptor subunit alpha-3	17
TAR00299	Mu-type opioid receptor	16
TAR00307	Estrogen receptor beta	16
TAR03025	Cyclin-A2	15
TAR00290	Sodium-dependent serotonin transporter	15
TAR00647	Serine/threonine-protein kinase Chk1	15
TAR00175	5-hydroxytryptamine 2A receptor	15
TAR04087	Caspase-3	15
TAR00123	Coagulation factor VII	14

TAR01201	Ig gamma-1 chain C region	14
TAR00565	Amine oxidase [flavin-containing] B	14
TAR00141	Gamma-aminobutyric-acid receptor subunit alpha-2	14
TAR00086	Apoptosis regulator Bcl-2	14
TAR00414	Transcription factor AP-1	14
TAR00186	Sodium-dependent noradrenaline transporter	13
TAR00239	Sodium-dependent dopamine transporter	13
TAR04565	Transcription factor p65	13
TAR04033	Apoptosis regulator BAX	13
TAR00063	Beta-1 adrenergic receptor	12
TAR00007	D(1A) dopamine receptor	12
TAR00402	Mitogen-activated protein kinase 14	12
TAR00272	Alpha-1D adrenergic receptor	11
TAR00229	Tyrosine-protein phosphatase non-receptor type 1	11
TAR00166	RAC-alpha serine/threonine-protein kinase	11
TAR04090	Caspase-9	11
TAR00727	Alcohol dehydrogenase 1C	11
TAR03727	Calcium-activated potassium channel subunit alpha 1	10
TAR00154	Muscarinic acetylcholine receptor M4	10
TAR03216	Glutamate receptor 2	9
TAR00646	Cellular tumor antigen p53	9
TAR00288	Aldose reductase	9
TAR03284	Gamma-aminobutyric-acid receptor subunit alpha-6	8
TAR00521	Leukotriene A-4 hydrolase	8
TAR00740	Vascular endothelial growth factor A	8
TAR00023	Matrix metalloproteinase-9	8
TAR00181	Gamma-aminobutyric-acid receptor subunit alpha-5	8
TAR00733	Glutathione S-transferase P	8
TAR00265	Tumor necrosis factor	8
TAR00273	Urokinase plasminogen activator surface receptor	8
TAR00087	Muscarinic acetylcholine receptor M5	7
TAR04089	Caspase-8	7
TAR00450	Protein kinase C alpha type	7
TAR00385	Transforming growth factor beta-1	7
TAR00357	Serum paraoxonase/arylesterase 1	7
TAR00105	Alpha-2A adrenergic receptor	7
TAR04141	Cyclin-dependent kinase inhibitor 1	7
TAR00568	Xanthine dehydrogenase/oxidase	7
TAR00353	Interstitial collagenase	7
TAR00139	Vascular endothelial growth factor receptor 2	6
TAR00117	Carbonic anhydrase 2	6
TAR00431	Cell division control protein 2 homolog	6
TAR04219	G2/mitotic-specific cyclin-B1	6
TAR03204	Aryl hydrocarbon receptor	6
TAR00284	Neuronal acetylcholine receptor subunit alpha-2	6
TAR20070	Heme oxygenase 1	6
TAR00432	Intercellular adhesion molecule 1	6
TAR00011	Insulin receptor	6

TAR04535	Solute carrier family 2, facilitated glucose transporter member 4	6
TAR00351	Interleukin-6	6
TAR00566	Amine oxidase [flavin-containing] A	6
TAR01696	Chymotrypsinogen B	6
TAR04262	Hyaluronan synthase 2	5
TAR00163	Delta-type opioid receptor	5
TAR00308	Glucocorticoid receptor	5
TAR00592	Hypoxia-inducible factor 1 alpha	5
TAR02971	Cation-independent mannose-6-phosphate receptor	5
TAR00337	Dual specificity mitogen-activated protein kinase kinase 1	5
TAR00371	Nuclear receptor subfamily 1 group I member 2	5
TAR00238	72 kDa type IV collagenase	5
TAR04394	NF-kappa-B inhibitor alpha	5
TAR02952	Glutathione S-transferase Mu 1	5
TAR03688	Glutathione S-transferase Mu 2	5
TAR04007	Activator of 90 kDa heat shock protein ATPase homolog 1	5
TAR00407	G1/S-specific cyclin-D1	5
TAR00354	Mitogen-activated protein kinase 1	5
TAR00113	5-hydroxytryptamine 3 receptor	4
TAR00126	Alpha-2C adrenergic receptor	4
TAR00448	Proto-oncogene protein c-fos	4
TAR00428	Myeloperoxidase	4
TAR00597	Superoxide dismutase [Cu-Zn]	4
TAR04303	Interleukin-8	4
TAR00726	Alcohol dehydrogenase 1B	4
TAR04525	Signal transducer and activator of transcription 1-alpha/beta	4
TAR00621	Cytochrome P450 3A4	4
TAR00724	Cytochrome P450 1A2	4
TAR20438	Cytochrome P450 1A1	4
TAR00427	E-selectin	4
TAR00440	Vascular cell adhesion protein 1	4
TAR20105	Cytochrome P450 1B1	4
TAR00088	Arachidonate 5-lipoxygenase	4
TAR03993	26S proteasome non-ATPase regulatory subunit 3	4
TAR04589	Type I iodothyronine deiodinase	4
TAR00707	Nuclear receptor subfamily 1 group I member 3	4
TAR00298	Epidermal growth factor receptor	4
TAR00661	Apoptosis regulator Bcl-X	4
TAR04292	Interleukin-10	4
TAR02915	Retinoblastoma-associated protein	4
TAR00380	DNA topoisomerase 1	4
TAR00196	Receptor tyrosine-protein kinase erbB-2	4
TAR04044	Baculoviral IAP repeat-containing protein 5	4
TAR03978	Interleukin-2	4
TAR00365	Interferon gamma	4
TAR00287	DNA topoisomerase 2-alpha	4
TAR04107	CD40 ligand	4
TAR00029	Acetyl-CoA acetyltransferase, mitochondrial	3

TAR03586	cAMP-dependent protein kinase inhibitor alpha	3
TAR00789	Retinoic acid receptor RXR-beta	3
TAR00349	Hepatocyte growth factor receptor	3
TAR00338	Protein kinase C beta type	3
TAR04301	Interleukin-4	3
TAR00648	Amyloid beta A4 protein	3
TAR04391	Neutrophil cytosol factor 1	3
TAR00396	Peroxisome proliferator-activated receptor delta	3
TAR00704	Mitogen-activated protein kinase 8	3
TAR04269	Induced myeloid leukemia cell differentiation protein Mcl-1	3
TAR00030	Peroxisome proliferator-activated receptor alpha	3
TAR00593	Prostaglandin E2 receptor, EP3 subtype	3
TAR00417	Small inducible cytokine A2	3
TAR04197	Eukaryotic translation initiation factor 6	3
TAR00350	Glycogen phosphorylase, muscle form	2
TAR04211	Fos-related antigen 1	2
TAR04212	Fos-related antigen 2	2
TAR03611	Cytochrome c	2
TAR04629	Arachidonate 12-lipoxygenase, 12S-type	2
TAR04399	Nuclear factor of activated T-cells, cytoplasmic 1	2
TAR04582	Tudor domain-containing protein 7	2
TAR03433	Egl nine homolog 1	2
TAR04381	NADPH oxidase 5	2
TAR20227	Fatty acid-binding protein, epidermal	2
TAR04032	Apolipoprotein D	2
TAR00203	5-hydroxytryptamine 2C receptor	2
TAR00374	Fatty acid synthase	2
TAR00246	Cytosolic phospholipase A2	2
TAR00092	4-aminobutyrate aminotransferase, mitochondrial	2
TAR00458	Inhibitor of nuclear factor kappa-B kinase subunit beta	2
TAR04028	Antileukoproteinase	2
TAR00222	Aldo-keto reductase family 1 member C3	2
TAR03971	Serine/threonine-protein phosphatase 2B catalytic subunit alpha isoform	2
TAR00573	Cell division protein kinase 4	2
TAR00395	Ubiquitin-protein ligase E3 Mdm2	2
TAR04450	Proliferating cell nuclear antigen	2
TAR03575	Caspase-7	2
TAR00091	Tyrosinase	2
TAR04456	Prostaglandin E synthase	2
TAR04311	Kinetochore protein Nuf2	2
TAR04015	Adenylate cyclase type 2	2
TAR00528	Poly [ADP-ribose] polymerase 1	2
TAR00470	NAD(P)H dehydrogenase [quinone] 1	2
TAR04398	Nuclear factor erythroid 2-related factor 2	2
TAR20103	ATP-binding cassette sub-family G member 2	2
TAR04297	Interleukin-1 alpha	2
TAR04620	Phosphatidylinositol-3,4,5-trisphosphate 3-phosphatase and dual-specificity protein phosphatase PTEN	2

TAR00731	Collagen alpha-1(I) chain	2
TAR00084	Plasminogen activator inhibitor 1	2
TAR00457	Thrombomodulin	2
TAR00434	Tissue-type plasminogen activator	2
TAR00318	Maltase-glucoamylase, intestinal	2
TAR00781	Estrogen sulfotransferase	2
TAR04247	Heat shock protein beta-1	2
TAR04174	Dual oxidase 2	2
TAR00418	Interleukin-1 beta	2
TAR00436	Gap junction alpha-1 protein	2
TAR00466	Tissue factor	2
TAR20929	Myc proto-oncogene protein	2
TAR04095	Caveolin-1	2
TAR00231	Acetyl-CoA carboxylase 1	2
TAR02963	78 kDa glucose-regulated protein	2
TAR04464	Protein CBFA2T1	2
TAR00153	Ornithine decarboxylase	2
TAR00744	NADPH--cytochrome P450 reductase	2
TAR04194	ETS domain-containing protein Elk-1	2
TAR04144	Cyclin-dependent kinase inhibitor 2A, isoforms 1/2/3	2
TAR00734	Pro-epidermal growth factor	2
TAR00441	Stromelysin-1	2
TAR04493	Ras GTPase-activating protein 1	2
TAR04259	Homeobox protein Nkx-3.1	2
TAR04252	Hexokinase-2	2
TAR04484	Puromycin-sensitive aminopeptidase	2
TAR04443	Procollagen C-endopeptidase enhancer 1	2
TAR04497	Receptor tyrosine-protein kinase erbB-3	2
TAR04290	Interferon regulatory factor 1	2
TAR00379	Insulin-like growth factor-binding protein 3	2
TAR00363	Cathepsin D	2
TAR02998	Prostatic acid phosphatase	2
TAR04561	Transcription factor E2F2	2
TAR04560	Transcription factor E2F1	2
TAR04492	Ras association domain-containing protein 1	2
TAR04505	Runt-related transcription factor 2	2
TAR04411	Osteopontin	2
TAR20873	Inhibitor of nuclear factor kappa-B kinase subunit alpha	2
TAR00342	C-reactive protein	2
TAR04246	Heat shock factor protein 1	2
TAR04125	Claudin-4	2
TAR04513	Serine/threonine-protein kinase Chk2	2
TAR04159	DDB1- and CUL4-associated factor 5	2
TAR02885	Collagen alpha-1(III) chain	2
TAR00012	RAF proto-oncogene serine/threonine-protein kinase	2
TAR03444	Tubulin beta-1 chain	1
TAR00090	Purine nucleoside phosphorylase	1
TAR03868	cAMP and cAMP-inhibited cGMP 3',5'-cyclic phosphodiesterase 10A	1

TAR00056	D(1B) dopamine receptor	1
TAR00106	5-hydroxytryptamine 1A receptor	1
TAR00173	Histamine H1 receptor	1
TAR00214	Alpha-2B adrenergic receptor	1
TAR00292	D(2) dopamine receptor	1
TAR00310	5-hydroxytryptamine 1B receptor	1
TAR03412	Gamma-aminobutyric acid receptor subunit gamma-3	1
TAR03967	Gamma-aminobutyric acid receptor subunit epsilon	1
TAR00424	Canalicular multispecific organic anion transporter 1	1
TAR00595	Serine/threonine-protein kinase mTOR	1
TAR00766	Glutathione S-transferase A1	1
TAR03674	Glutathione S-transferase A2	1
TAR04377	NAD-dependent deacetylase sirtuin-1	1
TAR02916	ATP synthase subunit beta, mitochondrial	1
TAR01245	NADH-ubiquinone oxidoreductase chain 6	1
TAR03154	3 beta-hydroxysteroid dehydrogenase/Delta 5-->4-isomerase type II	1
TAR00020	3 beta-hydroxysteroid dehydrogenase/Delta 5-->4-isomerase type I	1
TAR00377	Collagenase 3	1
TAR00468	Neutrophil collagenase	1
TAR00729	Alcohol dehydrogenase 1A	1
TAR20419	Folate receptor alpha	1
TAR00387	Beta-secretase 1	1
TAR00421	Mitogen-activated protein kinase 3	1
TAR00190	Low-density lipoprotein receptor	1
TAR00082	Microsomal triglyceride transfer protein large subunit	1
TAR04031	Apolipoprotein B-100	1
TAR04428	Phospholipase B1, membrane-associated	1
TAR20051	3-hydroxy-3-methylglutaryl-coenzyme A reductase	1
TAR00544	Cytochrome P450 19A1	1
TAR20436	UDP-glucuronosyltransferase 1-1	1
TAR04543	Sterol regulatory element-binding protein 1	1
TAR00240	Glutathione reductase, mitochondrial	1
TAR00306	Multidrug resistance-associated protein 1	1
TAR04017	Adiponectin	1
TAR20828	Sterol O-acyltransferase 2	1
TAR03236	Aldo-keto reductase family 1 member C1	1
TAR00202	Aspartate aminotransferase, cytoplasmic	1
TAR00627	Liver carboxylesterase 1	1
TAR03202	Sterol O-acyltransferase 1	1
TAR04341	Metalloproteinase inhibitor 1	1
TAR00373	cAMP response element-binding protein	1
TAR04509	Scavenger receptor cysteine-rich type 1 protein M130	1
TAR20780	Ephrin type-B receptor 2	1
TAR04362	Monocyte differentiation antigen CD14	1
TAR04322	Lipopolysaccharide-binding protein	1
TAR02889	Serum amyloid A protein	1
TAR04051	Bcl-2-binding component 3	1

TAR04549	Telomerase protein component 1	1
TAR00607	Protein kinase C delta type	1
TAR20110	Fibronectin	1

Supplementary Table S4A. The potential targets of Yihuo Huatan Formula

Target ID	Target Name	Degree
TAR00094	Prostaglandin G/H synthase 2	29
TAR00261	Beta-2 adrenergic receptor	13
TAR00482	Cell division protein kinase 2	13
TAR00733	Glutathione S-transferase P	12
TAR00402	Mitogen-activated protein kinase 14	12
TAR00265	Tumor necrosis factor	12
TAR00046	Estrogen receptor	11
TAR00175	5-hydroxytryptamine 2A receptor	10
TAR00353	Interstitial collagenase	10
TAR00095	Nitric-oxide synthase, endothelial	9
TAR00210	Muscarinic acetylcholine receptor M2	8
TAR00079	Coagulation factor X	7
TAR00414	Transcription factor AP-1	7
TAR00165	Acetylcholinesterase	6
TAR00048	Androgen receptor	6
TAR00422	Glycogen synthase kinase-3 beta	6
TAR00288	Aldose reductase	5
TAR00332	Dipeptidyl peptidase 4	5
TAR00307	Estrogen receptor beta	5
TAR00181	Gamma-aminobutyric-acid receptor subunit alpha-5	5
TAR00521	Leukotriene A-4 hydrolase	5
TAR00252	Mineralocorticoid receptor	5
TAR00299	Mu-type opioid receptor	5
TAR00491	Phosphatidylinositol-4,5-bisphosphate 3-kinase catalytic subunit gamma isoform	5
TAR00070	Sodium channel protein type 5 subunit alpha	5
TAR00646	Cellular tumor antigen p53	5
TAR00038	Muscarinic acetylcholine receptor M1	4
TAR00154	Muscarinic acetylcholine receptor M4	4
TAR00017	Prothrombin	4
TAR00273	Urokinase plasminogen activator surface receptor	4
TAR00105	Alpha-2A adrenergic receptor	3
TAR00086	Apoptosis regulator Bcl-2	3
TAR00565	Amine oxidase [flavin-containing] B	3
TAR00172	cGMP-inhibited 3',5'-cyclic phosphodiesterase A	3
TAR00191	Alpha-1A adrenergic receptor	2
TAR00016	Muscarinic acetylcholine receptor M3	2
TAR00087	Muscarinic acetylcholine receptor M5	2
TAR00309	Gamma-aminobutyric-acid receptor subunit alpha-1	2
TAR00141	Gamma-aminobutyric-acid receptor subunit alpha-2	2
TAR00200	Gamma-aminobutyric-acid receptor subunit alpha-3	2
TAR00444	Heat shock protein HSP 90-alpha	2
TAR00006	Prostaglandin G/H synthase 1	2
TAR00740	Vascular endothelial growth factor A	2
TAR00727	Alcohol dehydrogenase 1C	1
TAR00216	Alpha-1B adrenergic receptor	1

TAR00272	Alpha-1D adrenergic receptor	1
TAR00647	Serine/threonine-protein kinase Chk1	1
TAR00007	D(1A) dopamine receptor	1
TAR00123	Coagulation factor VII	1
TAR00037	Potassium voltage-gated channel subfamily H member 2	1
TAR00003	Nitric oxide synthase, inducible	1
TAR00209	Progesterone receptor	1
TAR00357	Serum paraoxonase/arylesterase 1	1
TAR00158	Retinoic acid receptor RXR-alpha	1
TAR00186	Sodium-dependent noradrenaline transporter	1
TAR00239	Sodium-dependent dopamine transporter	1
TAR00290	Sodium-dependent serotonin transporter	1
TAR00568	Xanthine dehydrogenase/oxidase	1

Supplementary Table S4B. The corresponding diseases of potential targets

Disease ID	Disease Name	Degree
DIS00130	unspecific cancer	9
DIS00051	Alzheimers Disease	8
DIS00416	Inflammation	8
DIS00144	unspecified cardiovascular disease	8
DIS00533	Myocardial Infarction	7
DIS00570	Non-small Cell Lung Cancer	6
DIS00607	Pain	6
DIS00659	Prostate cancer	6
DIS00711	Schizophrenia	6
DIS00744	Solid tumors	6
DIS00072	unspecified anxiety disorder	6
DIS00117	Breast cancer	5
DIS00397	Hypertension	5
DIS00567	Noninsulin-dependent diabetes mellitus	5
DIS00621	Parkinson Disease	5
DIS00077	Asthma	4
DIS00113	Brain injury	4
DIS00121	Breast Neoplasms	4
DIS00137	Cardiac arrhythmias	4
DIS00196	Cognitive deficits	4
DIS00228	Depression	4
DIS00353	Heart Failure	4
DIS00429	Insomnia	4
DIS00772	Thrombosis	4
DIS00182	Chronic Obstructive Pulmonary Disease	3
DIS00088	Autoimmune Diseases	3
DIS00103	Bladder cancer	3
DIS00176	Chronic lymphocytic leukemia	3
DIS00192	Coagulative disorders	3
DIS00201	Colorectal Neoplasms	3
DIS00243	Diabetic neuropathy	3
DIS00364	Hepatocellular Carcinoma	3
DIS00471	Lung Cancer	3
DIS00517	Migraine	3
DIS00550	Neurodegenerative diseases	3
DIS00586	Osteoarthritis	3
DIS00702	Rheumatoid arthritis	3
DIS00754	Stroke	3
DIS00703	Unspecified Rheumatoid Arthritis	3
DIS00810	Vascular disease	3
DIS00001	Abdominal aortic aneurysm	2
DIS00012	Acute lymphoblastic leukemia	2
DIS00015	Acute myeloid leukemia	2
DIS00022	Acute ureteric colic	2
DIS00024	Adenomatous polyposis	2
DIS00028	Adult respiratory distress syndrome	2

DIS00032	Advanced solid tumors	2
DIS00039	Airway hyperreactivity	2
DIS00041	Alcoholism	2
DIS00043	Allergic airway inflammation	2
DIS01002	Androgen insensitivity	2
DIS00062	Angina	2
DIS00398	Angina Hypertension	2
DIS00064	Angioedema	2
DIS00073	Anxiety Disorders	2
DIS00074	Arterial embolism and thrombosis	2
DIS00075	Arthritis	2
DIS00083	Atrial fibrillation and flutter	2
DIS00086	Autoimmune and sudden sensorineural hearing loss	2
DIS00087	Autoimmune cardiomyopathy	2
DIS00491	B cell malignancies	2
DIS00097	Behcet disease	2
DIS00098	Benign prostate hyperplasia	2
DIS00100	Bipolar Affective Disorder	2
DIS01001	Blood group_Yt system	2
DIS00143	cardiovascular disease	2
DIS00147	Carpal tunnel syndrome	2
DIS00150	Central nervous system diseases	2
DIS00164	Chondrosarcoma	2
DIS00179	Chronic myeloid leukemia	2
DIS00193	Cocaine dependence	2
DIS00124	cold air-induced bronchoconstriction	2
DIS00199	Colon cancer	2
DIS00200	Colorectal cancer	2
DIS00203	Congestive Heart Failure	2
DIS00205	Coronary Artery Disease	2
DIS00206	Coronary atherosclerosis	2
DIS00210	Cough	2
DIS00211	Crescentic glomerulonephritis	2
DIS00212	Crohns Disease	2
DIS00224	Delirium	2
DIS00226	Dementia	2
DIS00234	Diabetes mellitus	2
DIS00239	Diabetic complications	2
DIS00242	Diabetic nephropathy	2
DIS00244	Diabetic retinopathy	2
DIS00246	Diarrhea	2
DIS00265	Dyspnea	2
DIS01019	Dysprothrombinemia	2
DIS00269	Emphysema	2
DIS00270	Endocrine independent cancer	2
DIS00273	Endometriosis	2
DIS00274	Endotoxemia	2
DIS00280	Epileptic seizures	2
DIS00282	ER beta-positive prostate tumors	2

DIS00286	Essential primary hypertension	2
DIS00319	Gastrointestinal Neoplasms	2
DIS00325	Genitourinary tumors	2
DIS00327	Gestational hypertension	2
DIS00330	Glaucoma	2
DIS00336	Gout	2
DIS00347	Head and Neck Neoplasms	2
DIS00356	Helminth infection	2
DIS00125	histamine induced bronchospasm	2
DIS00513	Hormone-refractory Prostate cancer	2
DIS00392	Hyperimmunoglobulinemia D	2
DIS01017	Hyperkalemic distal renal tubular acidosis	2
DIS00402	Hypertrophic vascular disease	2
DIS01020	Hypoprothrombinemia	2
DIS00409	Hypothermia	2
DIS00410	Hypoxic-ischemic encephalopathy	2
DIS00413	Immunodeficiency	2
DIS00430	Insulin resistance	2
DIS00441	Ischemia	2
DIS00442	Ischemia reperfusion injuries	2
DIS00443	Ischemic heart disease	2
DIS00446	Kaposi Sarcoma	2
DIS00447	Kidney Cancer	2
DIS00485	Macular Degeneration	2
DIS00488	Major Depressive Disorder	2
DIS00491	Malignancies	2
DIS00496	Malignant mesothelioma	2
DIS00499	Manic disorder	2
DIS00505	Melanoma	2
DIS00508	Meningioma	2
DIS00525	Motor neurone disease	2
DIS00129	multidrug resistant cancer	2
DIS00527	Multiple Myeloma	2
DIS00529	Multiple Sclerosis	2
DIS00014	Myeloid Leukemia	2
DIS00537	Nasopharyngeal Cancer	2
DIS00542	neoplasm	2
DIS00552	Neurogenic bladder	2
DIS00556	Neurologic and psychiatric diseases	2
DIS00558	Neurological diseases	2
DIS00561	Neuropathic pain	2
DIS00563	Neurotoxicity Syndromes	2
DIS00564	Neutropenia	2
DIS00565	Non-Hodgkin Lymphoma	2
DIS00572	Obesity	2
DIS00574	Obstructive airway disease	2
DIS00577	Oesophageal cancer	2
DIS00578	Opioid dependence	2
DIS00579	Opioid-induced bowel dysfunction	2

DIS00585	Oropharyngeal squamous cell carcinoma	2
DIS00592	Osteoporosis	2
DIS00597	Ovarian Neoplasms	2
DIS00612	Pancreatic Cancer	2
DIS00620	Parkinsonian symptoms	2
DIS00622	Pathological angiogenesis	2
DIS00626	Periodic fever syndrome	2
DIS00632	Peripheral Nervous System Diseases	2
DIS00633	Peutz Jeghers syndrome	2
DIS00647	Postmenopausal symptoms	2
DIS00651	Precursor Cell Lymphoblastic Leukemia	2
DIS01016	Pseudohypoaldosteronism type I	2
DIS00668	Psoriasis	2
DIS00680	Rectal Neoplasms	2
DIS00684	Refractory partial epilepsy	2
DIS00689	Renal Cell Carcinoma	2
DIS00699	Respiratory distress syndrome	2
DIS00701	Rheumatic diseases	2
DIS00720	Sepsis	2
DIS00726	Shy Drager syndrome	2
DIS00734	Skeletal muscle wasting	2
DIS00735	Skeletal muscle weakness	2
DIS00737	Skin diseases	2
DIS00747	Spinal and bulbar muscular atrophy	2
DIS01004	Spinal and bulbar muscular atrophy of Kennedy	2
DIS00757	Sustained ventricular tachycardia	2
DIS00770	Thromboembolic disorders	2
DIS00771	Thromboembolism	2
DIS00773	Thrombotic disease	2
DIS00789	Tremor	2
DIS00213	unspecified crohns disease	2
DIS00264	Unspecified Dysmenorrhea	2
DIS00823	Unspecified Viral infection	2
DIS00802	Urge incontinence	2
DIS00813	Vascular injury response	2
DIS00814	Vascular lesion regression	2
DIS00833	Waldenstrom macroglobulinemia	2
DIS01003	X-linked Hypospadias 1	2
DIS01005	XY disorders of sex development	2
